# Cigar, Pipe, and Smokeless Tobacco Use and Cardiovascular Outcomes From Cross Cohort Collaboration

**DOI:** 10.1001/jamanetworkopen.2024.53987

**Published:** 2025-01-13

**Authors:** Erfan Tasdighi, Zhiqi Yao, Kunal K. Jha, Zeina A. Dardari, Ngozi Osuji, Tanuja Rajan, Ellen Boakye, Carlos J. Rodriguez, Kunihiro Matsushita, Eleanor M. Simonsick, João A. C. Lima, Rachel Widome, Debbie Cohen, Lawrence J. Appel, Amit Khera, Michael E. Hall, Suzanne Judd, Shelley A. Cole, Vasan S. Ramachandran, Emelia J. Benjamin, Aruni Bhatnagar, Andrew P. DeFilippis, Michael J. Blaha

**Affiliations:** 1 Johns Hopkins Ciccarone Center for Prevention of Cardiovascular Disease, Baltimore, Maryland; 2 The American Heart Association Tobacco Regulation and Addiction Center, Dallas, Texas; 3 Division of Cardiovascular Medicine, Albert Einstein College of Medicine, Bronx, New York; 4 Department of Epidemiology, Welch Center for Prevention, Epidemiology, and Clinical Research, Johns Hopkins Bloomberg School of Public Health, Baltimore, Maryland; 5 Department of Epidemiology, Johns Hopkins Bloomberg School of Public Health, Baltimore, Maryland; 6 Division of Cardiology, Department of Medicine, Johns Hopkins University, Baltimore, Maryland; 7 Division of Epidemiology and Community Health, University of Minnesota School of Public Health; 8 Renal, Electrolyte and Hypertension Division, Perelman School of Medicine, University of Pennsylvania, Philadelphia; 9 Division of Cardiology, Department of Internal Medicine, University of Texas Southwestern Medical Center, Dallas; 10 Department of Internal Medicine, Parkland Health and Hospital System, Dallas, Texas; 11 Department of Medicine, University of Mississippi Medical Center, Jackson; 12 Department of Biostatistics, University of Alabama at Birmingham School of Public Health; 13 Population Health Program, Texas Biomedical Research Institute, San Antonio; 14 University of Texas School of Public Health San Antonio; 15 Department of Medicine, University of Texas Health Science Center, San Antonio; 16 Cardiovascular Medicine, Boston Medical Center, Boston University Chobanian and Avedisian School of Medicine, Boston, Massachusetts; 17 Department of Epidemiology, Boston University School of Public Health, Boston, Massachusetts; 18 University of Louisville School of Medicine, Louisville, Kentucky; 19 Department of Medicine, Vanderbilt University Medical Center, Nashville, Tennessee

## Abstract

**Importance:**

Cardiovascular health outcomes associated with noncigarette tobacco products (cigar, pipe, and smokeless tobacco) remain unclear, yet such data are required for evidence-based regulation.

**Objective:**

To investigate the association of noncigarette tobacco products with cardiovascular health outcomes.

**Design, Setting, and Participants:**

This cohort study was conducted within the Cross Cohort Collaboration Tobacco Working Group by harmonizing tobacco-related data and conducting a pooled analysis from 15 US-based prospective cohorts with data on the use of at least 1 noncigarette tobacco product ranging between 1948 and 2015. The analysis for this study was conducted between September 2023 and February 2024. The median (IQR) follow-up time for the all-cause mortality outcome was 13.8 (10.2-19.2) years.

**Exposure:**

Current, sole, and exclusive use of noncigarette tobacco products. Sole use refers to using a noncigarette tobacco product without currently smoking cigarettes. Exclusive use means using only the noncigarette tobacco product and never having smoked cigarettes.

**Main Outcomes and Measures:**

Myocardial infarction, stroke, heart failure, atrial fibrillation, total coronary heart disease, total cardiovascular disease (CVD), coronary heart disease mortality, CVD mortality, and all-cause mortality.

**Results:**

Of 103 642 participants (mean [SD] age, 55.7 [13.2] years; 49 550 female [47.8%] and 54 092 male [52.2%]), current use rates were 26 962 participants (26.3%) for cigarettes, 1147 participants (2.1%) for cigars, 530 participants (1.2%) for pipes, and 1410 participants (2.1%) for smokeless tobacco. Current cigar use was associated with stroke (hazard ratio [HR], 1.25; 95% CI, 1.01-1.55), atrial fibrillation (HR, 1.32; 95% CI, 1.13-1.53), and heart failure (HR, 1.29; 95% CI, 1.10-1.51) compared with never using cigars in the model adjusted for demographic and socioeconomic factors, cardiovascular risk factors, and cohort. Sole (HR, 1.34; 95% CI, 1.12-1.62) and exclusive (HR, 1.53; 95% CI, 1.20-1.96) cigar use was associated with stroke compared with never using cigars or cigarettes. Current pipe use was associated with heart failure (HR, 1.23; 95% CI, 1.01-1.49) compared with never using pipes, and sole pipe use was associated with myocardial infarction (HR, 1.43; 95% CI, 1.17-1.74) compared with never using pipes or cigarettes. Current use of smokeless tobacco was associated with coronary heart disease mortality (HR, 1.31; 95% CI, 1.08-1.59) and myocardial infarction (HR, 1.20; 95% CI, 1.03-1.39) compared with never using smokeless tobacco. Sole and exclusive smokeless tobacco use demonstrated associations with total CVD (HR, 1.34; 95% CI, 1.19-1.50 and HR, 1.34; 955 CI, 1.13-1.59, respectively), total coronary heart disease (HR, 1.41; 95% CI, 1.21-1.64 and HR, 1.36; 95% CI, 1.08-1.70, respectively), heart failure (HR, 1.41; 95% CI, 1.22-1.64 and HR, 1.70; 95% CI, 1.40-2.06, respectively), and cardiovascular (HR, 1.41; 95% CI, 1.20-1.65 and HR, 1.54; 95% CI, 1.24-1.91, respectively) and all-cause (HR, 1.46; 95% CI, 1.34-1.60 and HR, 1.39; 95% CI, 1.22-1.58, respectively) mortality compared with never using smokeless tobacco or cigarettes.

**Conclusions and Relevance:**

In this study, there were distinct risk patterns associated with the use of noncigarette tobacco products. These findings may carry implications for public health and regulation of noncigarette tobacco products.

## Key Points

**Question:**

How are noncigarette tobacco products associated with cardiovascular health?

**Findings:**

This cohort study among 103 642 adults found that current use of cigars was associated with increased risk of stroke, atrial fibrillation, and heart failure, while current pipe use was associated with increased risk of heart failure. Users of smokeless tobacco products had increased risk for myocardial infarction and mortality due to coronary heart disease.

**Meaning:**

This study identified distinctive cardiovascular risks associated with the use of noncigarette tobacco products, underscoring the substantial public health implications and highlighting the need for stringent regulatory measures.

## Teaser

This cohort study investigates the association of noncigarette tobacco products with cardiovascular health outcomes and mortality.

## Introduction

Cardiovascular disease (CVD) is a major cause of morbidity and mortality worldwide, also causing substantial strain on global health care resources.^1,2^ Combustible cigarette smoking is a well-established causal risk factor for CVD and is estimated to be responsible for 20% of global deaths from coronary heart disease (CHD).^2,3,4,5,6,7^ Although combustible cigarette use has decreased, noncigarette tobacco product usage has remained constant or increased in recent decades.^8,9,10^ For example, self-reported survey data indicated that 3.6% of US adults (8.7 million individuals) smoked cigars in 2019, making cigars the second most used combustible tobacco product, behind combustible cigarettes, among US adults.^11,12^ From 2016 to 2020, monthly cigarillo unit sales increased from approximately 131 million to 190 million by a mean of 0.7% per month in the US.^13^ Of note, the inclusion in cigars of flavors (eg, cherry), which are banned in cigarettes, and their frequent sale as individual sticks have intensified concerns that these products may be purposefully marketed to be especially appealing to young individuals.^14^ An estimated 0.9% of US adults were reported to have used pipes, including hookah, in 2021.^15^ Moreover, there was a 23% increase in smokeless tobacco use among US adults between 2000 and 2015.^16^

While the associations of combustible cigarettes with CVD are well known, there remain critical knowledge gaps regarding health associations of noncigarette tobacco products. The lack of a clear assessment of the risk associated with tobacco products other than cigarettes has been a major impediment to evidence-based regulation of these products by the US Food and Drug Administration (FDA) and has perpetuated the belief that these might be reduced-harm tobacco products.^17,18^

One barrier to studying noncigarette tobacco products is their lower use prevalence compared with that of combustible cigarettes in most cohorts. We harmonized tobacco-related data from 15 National Institutes of Health National Heart, Lung, and Blood Institute prospective cohorts as part of the Cross Cohort Collaboration Tobacco Working Group (CCC-Tobacco) study to overcome these limitations and inform the tobacco regulatory science community about the cardiovascular health risk associated with the use of noncigarette tobacco products.^19^ Our primary objectives were to offer estimates of cardiovascular outcomes associated with the use of cigars, pipes, and smokeless tobacco compared with nonuse of tobacco and make comparisons with point estimates of risk associated with combustible cigarettes.

## Methods

This cohort study was approved by the Johns Hopkins School of Medicine Institutional Review Board, which granted a waiver regarding informed consent because this study used only data previously collected under individual cohort–level IRBs. The study adhered to the Strengthening the Reporting of Observational Studies in Epidemiology (STROBE) reporting guideline for observational studies.

### Study Population

CCC-Tobacco, a working group of the CCC, created a harmonized dataset of 23 prospective cohort studies spanning 322 782 participants predominantly from the US. The working group consists of 13 traditional cardiovascular cohorts and 10 noncardiovascular cohorts aimed at studying aging. The rationale and design of the CCC-Tobacco project have been previously described in detail.^19^

This study represents a subset of 15 CCC-Tobacco cohorts that had data on at least 1 noncigarette tobacco product between 1948 and 2015 (see the eMethods in Supplement 1 for each participating cohort's funding statement and more details). Table 1 lists cohorts included in this study. More detailed descriptions of these cohorts are shown in eTable 1 and the eMethods in Supplement 1.

**Table 1.** Participating Cohort Characteristics

Participating cohort	Cohort description	Baseline visit year
Atherosclerosis Risk in Communities Study (ARIC)	15 784 Participants in 4 US communities aged 45-64 y	Visit 1 (1987-1989)
Baltimore Longitudinal Study of Aging (BLSA)	1575 Males and females aged >20 y	Visit 1 (1958)
Chronic Renal Insufficiency Cohort Study (CRIC)	5561 Participants with chronic kidney disease (1560 older adults during third phase)	Visit 3 (2013-2015)
Coronary Artery Risk Development in Young Adults (CARDIA)	2531 Participants at 4 US field centers aged 18-30 y	Year 5 (1990-1991)
Dallas Heart Study (DHS)	3557 Persons of from multiethnic cohort supported by the Hoffman Family Center and National Center for Advancing Translational Sciences, with annual surveys within hospitals in the Dallas Fort Worth metroplex	DHS 1 phase 1 (2003-2007)
Framingham Heart Study (FHS)	3753 Participants in the adult population of Framingham, Massachusetts, aged 30-62 y (original cohort)	Exam 7 (1968-1971)
	Offspring cohort: 4812 adult children of the original cohort and their spouses, aged 30-74 y	Exam 1 (1971-1975)
	FHS third generation: 4063 males and females aged >19 y with ≥1 parent in the offspring study	Exam 1 (2002-2005)
Health, Aging and Body Composition Study (Health ABC)	2958 Community-dwelling participants in Memphis, TN, or Pittsburgh, PA, and aged 70-79 y	Year 1 (1997-1998)
Jackson Heart Study (JHS)	5258 Community-based African American participants from 3 counties in Jackson, MS, aged 35-84 y	Visit 1 (2000-2004)
Multi-Ethnic Study of Atherosclerosis (MESA)	6789 Males and females from 6 multiethnic communities in the US aged 45-84 y	Visit 1 (2000-2002)
The Multiple Risk Factor Intervention Trial (MRFIT)	12 866 Males aged 35-57 y enrolled in coronary heart disease intervention trial	Visit 2 (1975-1976)
Rancho Bernardo Study (RBS) of Healthy Aging	462 Participants in community based cohort of all residents of Rancho Bernardo, San Diego, CA	Visit 4 (1984-1987)
Reasons for Geographic and Racial Differences in Stroke (REGARDS)	30 174 Black and White participants from the continental US	Visit 1 (2003-2007)
Strong Heart Study (SHS)	3485 American Indian tribal members aged 35-74 y	Phase 1 (1989-1991)

### Definitions of Tobacco Use in CCC-Tobacco

Given the high prevalence of patterns of polytobacco use (the use of multiple types of tobacco products concurrently) in the general population,^20,21^ we a priori defined noncigarette tobacco use (cigars, pipes, and smokeless tobacco) in 2 broad categories. For the rationale behind these categories described subsequently, please refer to the eMethods in Supplement 1. First is tobacco product–specific use status, defined as current use, former use, or never use (eFigure 1 in Supplement 1). Given the prevalence of polytobacco use, it is important to note that resultant categories for use status are not designed to be mutually exclusive across all products. For further details, please refer to the eMethods in Supplement 1.

Second, to better distinguish associations of noncigarette tobacco use from those of traditional combustible cigarette use, subgroups of current users were further classified into sole and exclusive use. Sole use was defined as current use of a noncigarette tobacco product, excluding individuals with concurrent combustible cigarette use. Exclusive use was defined as current use of a noncigarette tobacco product, excluding individuals with current or former combustible cigarette use. For the sole and exclusive analysis, the reference group for each type of tobacco product being studied consisted of individuals who had never smoked combustible cigarettes and were not currently using the specific noncigarette tobacco product in question. Please refer to the eMethods in Supplement 1 for further information. See eFigure 2 in Supplement 1 for a conceptual illustration of sole and exclusive cigar use. See eFigure 3 in Supplement 1 for the flowchart showing how we reached the analytic sample size of sole and exclusive users for each noncigarette tobacco product.

All noncigarette tobacco product use data were drawn from cohorts’ detailed questionnaires (eTable 2 in Supplement 1). When the most thorough tobacco questionnaire was not administered at the baseline visit of a cohort, the baseline for CCC-Tobacco was shifted to the earliest cohort visit with the most complete noncigarette tobacco use data. The prevalence of each tobacco product is given in eTable 3 in Supplement 1.

### Cardiovascular and Mortality Outcomes

We selected outcomes that were formally adjudicated across most participating cohorts. Moreover, we included various cardiovascular outcomes to provide comprehensive insights into potential cardiovascular risks associated with noncigarette tobacco products.

A total of 9 outcomes relevant to cardiovascular health were collected and harmonized in CCC-Tobacco: myocardial infarction, stroke, heart failure, atrial fibrillation, total CHD, total cardiovascular disease (CVD), CHD mortality, CVD mortality, and all-cause mortality. Total CHD events were defined as a composite of myocardial infarction, coronary revascularization, or coronary death. Total CVD events were defined as a composite of all atherosclerotic CVD events, including CHD, stroke, or cardiovascular death (coronary death, stroke death, other atherosclerotic death, or other CVD death).

Cardiovascular outcomes in this study were harmonized across the 15 cohorts by using each cohort’s specific definitions of outcomes. In most cases, these were adjudicated by a dedicated adjudication committee. If an individual cohort had some but not all components (eg, angina) of CHD or CVD composite events, components that were present were retained to represent a modified CHD or CVD composite for that cohort.

### Harmonization of Covariates

Definitions of all demographic, anthropomorphic, and traditional risk factors, including hypertension, diabetes, hyperlipidemia, and dyslipidemia, have been previously described.^19^ Harmonization of covariates for race and ethnicity was based on self-reported data and adhered to the data-collection protocols of each cohort. Race and ethnicity categories in the dataset were combined and harmonized for this study as American Indian or Alaska Native, Asian, Black or African American, Hispanic, White and other (participants with missing information on race and ethnicity were categorized as other in this study). For further details, please refer to the eMethods in Supplement 1.

### Statistical Analysis

Baseline characteristics are presented by baseline tobacco use status (combustible smoking: never, former, and current; cigar, pipe, or smokeless tobacco use: current, sole, and exclusive). The primary approach to analysis was pooled, individual-level data modeling. Secondary analysis was conducted using weighted cohort-level random associations meta-analysis. Adjusted Cox proportional hazard models were used to evaluate the association between tobacco use and the 9 study outcomes. The start time of the Cox proportional hazard models was the time of tobacco product assessment at each cohort. Median follow-up times and total number of observations and events for each outcome across cohorts are shown in eTables 4 and 5 in Supplement 1.

The first multivariable model was adjusted for age, sex, and race and ethnicity (American Indian or Alaska Native, Asian, Black or African American, Hispanic, White, and other), education status (did not complete high school, completed high school, and ≥college degree), history of coronary heart disease, cohort, and cigarette smoking status. The specific cohort was included as a covariate to account for the baseline hazard of each cohort. The second multivariable model was additionally adjusted for harmonized covariates, including body mass index (calculated as weight in kilograms divided by height in meters squared), systolic blood pressure, diastolic blood pressure, diabetes, hyperlipidemia, antihypertensive medication use, lipid-lowering medication use, and any alcohol use. Of note, while models for current noncigarette tobacco use were adjusted for combustible cigarette smoking status, this term was not included in models where combustible smoking was the primary exposure.

Given that our dataset comprised pooled data from multiple cohorts, there was a potential for intragroup correlation within cohorts. To see our approach for evaluating intragroup correlation within cohorts, please refer to the eMethods in Supplement 1.

A 2-sided *P* value < .05 was considered statistically significant. All analyses were performed with Stata/SE statistical software version 17.0 (StataCorp). Data were analyzed between September 2023 and February 2024. There was a median (IQR) follow-up of 13.8 (10.2-19.2) years for the all-cause mortality outcome.

## Results

Among 103 642 participants (mean [SD] age, 55.7 [13.2] years; 49 550 female [47.8%] and 54 092 male [52.2%]; 3504 American Indian or Alaska Native [3.4%], 863 Asian [0.8%], 31 850 Black or African American [30.7%], 2821 Hispanic or Latino [2.7%], 63 880 White [61.8%], and 450 other race or ethnicity [0.4%]), 26 962 individuals (26.3%) reported current combustible cigarette smoking, 1147 individuals (2.1%) were current cigar users, 530 individuals (1.2%) were current pipe users, and 1410 individuals (2.1%) were current smokeless tobacco users. In general, compared with current users of combustible cigarette smoking, individuals who were current cigar or pipe users were similar in age but were more likely to be White males and had a higher educational attainment. Smokeless tobacco users had an older mean age, had a balanced racial distribution between White and Black individuals, and showed a more diverse educational background. Smokeless tobacco users demonstrated a more adverse cardiometabolic profile compared with that of their counterparts (Table 2).^22^

**Table 2.** Baseline Characteristics by Tobacco Product Use Status

Characteristic	Participants, No. (%) (N = 103 642)^a^											
	Cigarette			Cigar			Pipe			Smokeless tobacco		
	Never (n = 42 326 [41.3%])	Former (n = 33 124 [32.3%])	Current (n = 26 962 [26.3%])	Current (n = 1147 [2.1%])	Sole (n = 2583 [6.0%])^b^	Exclusive (n = 1122 [2.7%])^c^	Current (n = 523 [1.2%])	Sole (n = 974 [3.8%])	Exclusive (n = 423 [1.7%])	Current (n = 1410 [2.1%])	Sole (n = 1209 [3.9%])	Exclusive (n = 571 [1.9%])
Age, mean (SD), y	56.6 (13.7)	59.3 (12.3)	49.9 (11.6)	52.4 (13.2)	53.4 (12.0)	52.3 (12.2)	50.4 (11.8)	49.2 (10.0)	49.3 (10.4)	59.9 (11.1)	60.0 (11.8)	59.1 (13.2)
Sex												
Female	26130 (61.7)	12880 (38.9)	9977 (37.0)	51 (4.4)	71 (2.7)	27 (2.4)	4 (0.8)	6 (0.6)	1 (0.2)	377 (26.7)	333 (27.5)	251 (44.0)
Male	16196 (38.3)	20 244 (61.1)	16 985 (63.0)	1096 (95.6)	2512 (97.3)	1095 (97.6)	519 (99.2)	968 (99.4)	422 (99.8)	1033 (73.3)	876 (72.5)	320 (56.0)
Race and ethnicity^d^												
American Indian or Alaska Native	874 (2.1)	1181 (3.6)	1341 (4.9)	NA	NA	NA	NA	NA	NA	NA	NA	NA
Asian	653 (1.5)	160 (0.5)	50 (0.2)	NA	NA	NA	NA	NA	NA	NA	NA	NA
Black or African American	14 935 (35.4)	9425 (28.5)	7073 (26.2)	363 (31.7)	469 (18.1)	225 (20.1)	81 (15.3)	89 (9.1)	42 (10.0)	610 (43.3)	511 (42.3)	334 (58.5)
Hispanic or Latino	1496 (3.5)	825 (2.5)	372 (1.4)	47 (4.1)	32 (1.2)	14 (1.2)	NA	NA	NA	NA	NA	NA
White	24 096 (57.1)	21 312 (64.5)	17 914 (66.5)	728 (63.6)	2025 (78.4)	864 (77.1)	445 (83.9)	857 (88.0)	370 (87.4)	792 (56.3)	662 (54.7)	228 (39.9)
Other (missing data)	121 (0.3)	137 (0.4)	192 (0.7)	NA	40 (1.5)	15 (1.3)	NA	16 (1.6)	NA	NA	NA	NA
Education^d^												
High school not completed	6444 (15.2)	5386 (16.3)	5843 (21.7)	205 (17.9)	350 (13.5)	158 (14.1)	69 (13.0)	113 (11.6)	42 (9.9)	544 (38.6)	459 (38.0)	252 (44.1)
High school completed	10 928 (25.8)	8662 (26.1)	8338 (30.9)	299 (26.1)	546 (21.1)	224 (20.0)	150 (28.3)	185 (19.0)	79 (18.7)	444 (31.5)	378 (31.2)	162 (28.4)
≥College degree	24 111 (57.0)	18 585 (56.1)	11 786 (43.7)	574 (50.0)	1632 (63.2)	706 (62.9)	247 (46.6)	628 (64.5)	269 (63.6)	415 (29.4)	366 (30.3)	153 (26.8)
Alcohol use^e^	20 151 (48.6)	19 781 (61.7)	20 050 (76.5)	957 (83.6)	2141 (83.9)	919 (82.6)	417 (78.7)	845 (87.2)	367 (87.0)	598 (42.9)	500 (42.5)	206 (36.7)
BMI, mean (SD)	29.0 (6.3)	29.0 (5.7)	27.2 (5.2)	28.4 (5.0)	28.6 (4.5)	28.9 (4.6)	26.8 (3.7)	27.5 (3.6)	27.7 (3.6)	29.6 (6.0)	29.9 (6.0)	30.7 (6.5)
Hypertension	21 365 (50.5)	19 273 (58.2)	14 101 (52.3)	479 (41.8)	1725 (66.9)	707 (63.1)	156 (29.4)	651 (66.8)	261 (61.7)	789 (56.0)	677 (56.0)	327 (57.4)
SBP, mean (SD), mm Hg	126.8 (19.5)	128.9 (19.6)	130.1 (21.0)	126.2 (19.0)	139.0 (20.2)	135.4 (19.5)	123.6 (18.2)	138.2 (20.9)	136.1 (20.2)	127.4 (18.9)	127.6 (18.4)	128.1 (19.2)
DBP, mean (SD), mm Hg^e^	76.6 (12.0)	77.6 (13.2)	81.6 (14.5)	77.0 (11.9)	87.3 (15.3)	87.3 (15.1)	76.9 (12.0)	90.9 (15.5)	90.3 (15.5)	76.2 (11.1)	76.4 (10.7)	77.4 (11.2)
BP medication^e^	15 916 (38.5)	13 844 (43.1)	6207 (23.8)	335 (29.4)	771 (30.3)	295 (26.7)	77 (14.6)	190 (19.6)	74 (17.6)	708 (51.2)	599 (51.2)	270 (48.6)
Diabetes	6926 (16.7)	6570 (20.2)	3082 (11.7)	184 (16.3)	340 (13.4)	137 (12.5)	36 (6.9)	71 (7.4)	31 (7.5)	674 (48.8)	283 (24.0)	144 (26.1)
Dyslipidemia	23 846 (57.7)	20867 (64.3)	17 349 (65.6)	649 (58.5)	1760 (69.0)	745 (67.2)	289 (55.2)	671 (69.2)	270 (64.4)	873 (64.2)	751 (64.0)	326 (59.4)
HPL^e^	11 246 (27.5)	10152 (32.0)	10 833 (41.3)	324 (30.3)	1187 (46.9)	502 (45.4)	160 (30.6)	530 (54.8)	223 (53.3)	361 (27.3)	308 (26.7)	138 (25.5)
HPL medication^e^	7002 (17.0)	7536 (23.4)	2263 (8.6)	153 (13.6)	341 (13.3)	135 (12.1)	22 (4.2)	23 (2.4)	9 (2.1)	307 (22.0)	260 (21.9)	94 (16.8)
Cholesterol, mean (SD), mg/dL												
Total	201 (43)	201 (44)	212 (46)	197 (39)	216 (45)	214 (45)	201 (37)	226 (40)	225 (39)	195 (41)	195 (41)	196 (43)
LDL^f^	122 (37)	123 (39)	132 (41)	123 (37)	137 (40)	137 (40)	131 (35)	149 (36)	147 (35)	121 (37)	121 (36)	121 (38)
HDL	52 (16)	50 (15)	47 (15)	46 (14)	45 (13)	45 (13)	44 (13)	43 (12)	44 (11)	47 (15)	47 (15)	50.2 (17)
Triglycerides, median (IQR), mg/dL	104 (74-150)	115 (81-168)	122 (84-180)	112 (77-166)	133(91-194)	128 (85-188)	109 (78-158)	142 (96-203)	129 (88-196)	113 (79-165)	112 (80-163)	104 (75-156)

Abbreviations: BMI, body mass index (calculated as weight in kilograms divided by height in meters squared); BP, blood pressure; DBP, diastolic blood pressure; HDL, high-density lipoprotein; HPL, hyperlipidemia; LDL, low-density lipoprotein; NA, not applicable; SBP, systolic blood pressure.

SI conversion factors: To convert HDL, LDL, and total cholesterol to millimoles per liter, multiply by 0.0259; triglycerides to millimoles per liter, multiply by 0.0113.

^a^ The categories of this table are not mutually exclusive.

^b^ Sole use status was defined as current noncigarette tobacco use, excluding patients with a history of current cigarette use.

^c^ Exclusive smoking status was defined as current noncigarette tobacco use, excluding patients with a history of former and current cigarette use.

^d^ For race and ethnicity and education, cells that included fewer than 10 individuals could not be displayed based on National Institutes of Health US Renal Data System^22^ guidelines. For race and ethnicity, these cells are denoted NA. For education, other categories with such cells are omitted.

^e^ Missing values were imputed in the annotated variables.

^f^ Lipid profile was not measured in all cohorts (<10% of the dataset was imputed).

### Current Tobacco Product Use and CVD Outcomes

Table 3 presents hazard ratios (HRs) for the association between current use of each tobacco product and the cardiovascular and mortality outcomes evaluated. Total numbers of observations and events for each analysis are presented in eTable 6 in Supplement 1.

**Table 3.** Current Use vs Nonuse of Tobacco Products and Associated Health Outcomes

Outcome^b^	HR (95% CI)^a^			
	Cigarette (n = 26 962)	Cigar (n = 1147)	Pipe (n = 530)	Smokeless tobacco (n = 1410)
Myocardial infarction				
Participants, No.	3094	154	101	192
Model 1	1.65 (1.56-1.74)	1.05 (0.89-1.24)	1.04 (0.85-1.28)	1.30 (1.12-1.51)
Model 2	1.78 (1.69-1.89)	1.11 (0.94-1.32)	1.20 (0.98-1.48)	1.20 (1.03-1.39)
Stroke				
Participants, No.	1730	97	50	116
Model 1	1.48 (1.39-1.58)	1.24 (1.00-1.52)	1.05 (0.79-1.39)	1.14 (0.95-1.38)
Model 2	1.62 (1.51-1.73)	1.25 (1.01-1.55)	1.09 (0.82-1.46)	1.09 (0.90-1.32)
CHD				
Participants, No.	3998	200	112	254
Model 1	1.62 (1.54-1.69)	1.05 (0.90-1.21)	0.95 (0.78-1.15)	1.30 (1.14-1.47)
Model 2	1.76 (1.68-1.85)	1.11 (0.96-1.29)	1.09 (0.89-1.32)	1.19 (1.04-1.36)
CVD				
Participants, No.	6020	304	170	425
Model 1	1.63 (1.57-1.69)	1.11 (0.98-1.24)	1.00 (0.86-1.17)	1.30 (1.17-1.43)
Model 2	1.79 (1.73-1.87)	1.15 (1.02-1.30)	1.10 (0.94-1.29)	1.19 (1.08-1.32)
Heart failure				
Participants, No.	2822	182	112	286
Model 1	1.67 (1.59-1.76)	1.23 (1.06-1.43)	1.08 (0.89-1.30)	1.33 (1.17-1.50)
Model 2	1.99 (1.89-2.10)	1.29 (1.10-1.51)	1.23 (1.01-1.49)	1.20 (1.06-1.36)
Atrial fibrillation				
Participants, No.	2042	197	115	165
Model 1	1.44 (1.35-1.52)	1.31 (1.13-1.51)	0.97 (0.81-1.17)	1.20 (1.03-1.41)
Model 2	1.61 (1.52-1.71)	1.32 (1.13-1.53)	1.00 (0.82-1.21)	1.13 (0.96-1.33)
CHD mortality				
Participants, No.	1773	86	38	115
Model 1	1.68 (1.57-1.82)	1.09 (0.88-1.36)	0.83 (0.60-1.15)	1.42 (1.17-1.71)
Model 2	1.90 (1.77-2.05)	1.20 (0.96-1.50)	0.99 (0.71-1.38)	1.31 (1.08-1.59)
CVD mortality				
Participants, No.	3063	144	74	233
Model 1	1.67 (1.58-1.76)	1.14 (0.96-1.35)	0.91 (0.72-1.15)	1.36 (1.19-1.56)
Model 2	1.87 (1.77-1.98)	1.18 (0.99-1.40)	1.00 (0.79-1.28)	1.23 (1.07-1.41)
All-cause mortality				
Participants, No.	10 478	503	291	716
Model 1	1.98 (1.93-2.04)	1.14 (1.02-1.22)	1.05 (0.93-1.18)	1.26 (1.17-1.36)
Model 2	2.13 (2.07-2.20)	1.13 (1.03-1.24)	1.08 (0.96-1.22)	1.21 (1.11-1.30)

Abbreviations: CHD, coronary heart disease; CVD, cardiovascular disease; HR, hazard ratio.

^a^ The reference group consists of individuals who had never used the specific tobacco product under consideration.

^b^ Model 1 adjusted for age, sex, race and ethnicity, cigarette smoking status, education status, history of CHD, and cohort. Model 2 adjusted for age, sex, race and ethnicity, cigarette smoking status, education status, history of CHD, cohort, body mass index (calculated as weight in kilograms divided by height in meters squared), hypertension, diabetes, antihypertensive and lipid-lowering medication, systolic blood pressure, diastolic blood pressure, alcohol use, and hyperlipidemia. Model 1 and model 2 were not adjusted for cigarette smoking status for cigarette tobacco analysis.

In the more comprehensive adjusted model 2, current cigar use was positively associated with 6 of 9 outcomes studied compared with never smoking cigars. Cigar use was associated with stroke (HR of 1.25; 95% CI, 1.01-1.55). atrial fibrillation (HR, 1.32; 95% CI, 1.13-1.53), and heart failure (HR, 1.29; 95% CI, 1.10-1.51) compared with never smoking cigars (Table 3).

For individuals currently using pipes, the only association observed was with heart failure, with an HR of 1.23 (95% CI, 1.01-1.49) in model 2 compared with individuals who never smoked pipes. The remaining outcomes were nonsignificant (Table 3).

Smokeless tobacco use was positively associated with 8 of 9 outcomes studied compared with never using smokeless tobacco. CHD mortality had an HR of 1.31 (95% CI, 1.08-1.59). CVD mortality presented with an HR of 1.23 (95% CI, 1.07-1.41). There was also an association with myocardial infarction (HR, 1.20; 95% CI, 1.03-1.39) (Table 3).

The forest plot presented in the Figure illustrates HRs from the second model for the incidence of cardiovascular outcomes and cause-specific and all-cause mortality associated with current use of tobacco products, including cigarettes, cigars, pipes, and smokeless tobacco. The analysis revealed that cigarette smoking was consistently associated with a higher risk across all measured outcomes. In contrast, the use of cigars, pipes, and smokeless tobacco products showed associations with these adverse outcomes with generally lower HRs or showed a lack of association, with narrower CIs overlapping with 1.00 in several categories, indicating no statistical significance. Notably, there was increased heart failure risk across all tobacco product categories. All-cause mortality risk was increased for current use of all tobacco products except pipes.

**Figure.**
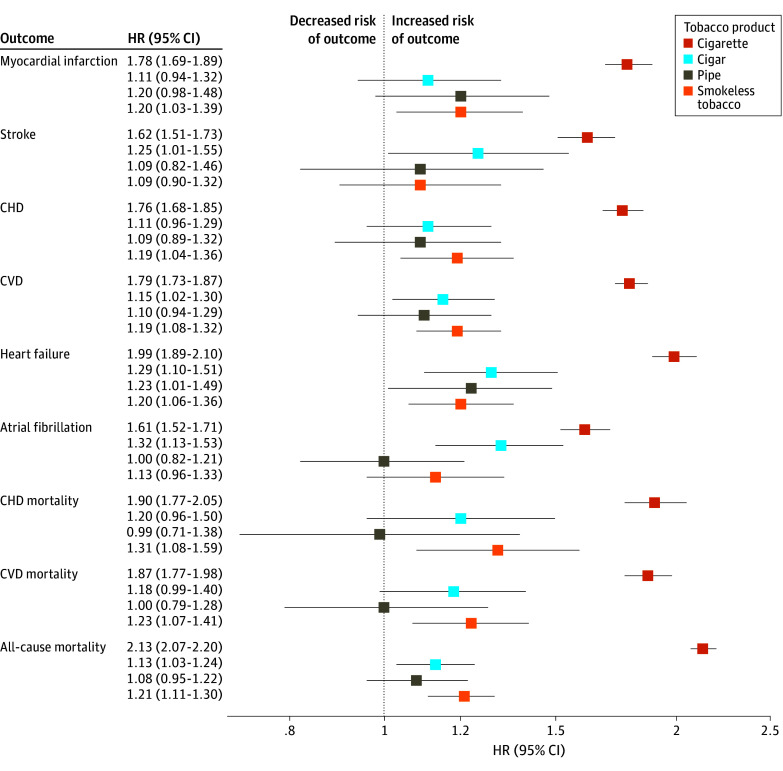
Risk of Cardiovascular and Mortality Outcomes Associated With Current Use of Tobacco Products CHD indicates coronary heart disease; CVD, cardiovascular disease; HR, hazard ratio.

### Sole and Exclusive Noncigarette Tobacco Product Use

For individuals reporting sole use of cigars, there was a positive association with stroke (HR, 1.34; 95% CI, 1.12-1.62), CVD (HR, 1.19; 95% CI, 1.07-1.32), and heart failure (HR, 1.33; 95% CI, 1.14-1.56) compared with those who never smoked cigars or cigarettes. Individuals reporting exclusive cigar use also exhibited increased risks compared with those who never smoked cigars or cigarettes, notably for stroke (HR, 1.53; 95% CI, 1.20-1.96), CVD (HR, 1.27; 95% CI, 1.10-1.46), and CVD mortality (HR, 1.36; 95% CI, 1.12-1.65) (Table 4).

**Table 4.** Sole and Exclusive Use vs Nonuse of Noncigarette Tobacco Products and Associated Health Outcomes

Outcome^b^	HR (95% CI)^a^					
	Cigar use status		Pipe use status		Smokeless use status	
	Sole (n = 2583)^c^	Exclusive (n = 1122)^d^	Sole (n = 974)	Exclusive (n = 423)	Sole (n = 1209)	Exclusive (n = 571)
Myocardial infarction						
Participants, No.	229	105	126	60	163	60
Model 1	1.11 (0.96-1.28)	1.05 (0.85-1.28)	1.31 (1.08-1.59)	1.18 (0.90-1.55)	1.57 (1.33-1.86)	1.34 (1.03-1.74)
Model 2	1.14 (0.98-1.33)	1.13 (0.92-1.39)	1.43 (1.17-1.74)	1.30 (0.99-1.71)	1.43 (1.20-1.70)	1.24 (0.94-1.63)
Stroke						
Participants, No.	142	76	51	26	98	46
Model 1	1.32 (1.10-1.59)	1.50 (1.18-1.91)	1.17 (0.87-1.57)	1.11 (0.75-1.66)	1.19 (0.96-1.47)	1.19 (0.89-1.61)
Model 2	1.34 (1.12-1.62)	1.53 (1.20-1.96)	1.20 (0.89-1.62)	1.12 (0.74-1.69)	1.13 (0.91-1.41)	1.12 (0.82-1.53)
CHD						
Participants, No.	299	137	142	65	220	88
Model 1	1.12 (0.99-1.27)	1.07 (0.89-1.27)	1.16 (0.97-1.39)	1.01 (0.78-1.30)	1.57 (1.36-1.81)	1.43 (1.15-1.79)
Model 2	1.17 (1.03-1.33)	1.16 (0.97-1.38)	1.26 (1.05-1.52)	1.11 (0.86-1.44)	1.41 (1.21-1.64)	1.36 (1.08-1.70)
CVD						
Participants, No.	456	225	198	95	356	161
Model 1	1.16 (1.04-1.28)	1.21 (1.05-1.39)	1.16 (0.99-1.35)	1.06 (0.86-1.30)	1.47 (1.31-1.64)	1.44 (1.22-1.69)
Model 2	1.19 (1.07-1.32)	1.27 (1.10-1.46)	1.23 (1.05-1.43)	1.12 (0.91-1.39)	1.34 (1.19-1.50)	1.34 (1.13-1.59)
Heart failure						
Participants, No.	196	97	90	33	230	123
Model 1	1.32 (1.13-1.54)	1.35 (1.09-1.66)	1.28 (1.02-1.59)	0.88 (0.62-1.25)	1.55 (1.35-1.79)	1.78 (1.48-2.14)
Model 2	1.33 (1.14-1.56)	1.37 (1.11-1.71)	1.42 (1.13-1.77)	0.96 (0.67-1.37)	1.41 (1.22-1.64)	1.70 (1.40-2.06)
Atrial fibrillation						
Participants, No.	190	104	100	45	134	46
Model 1	1.32 (1.13-1.54)	1.39 (1.14-1.71)	1.20 (0.97-1.48)	1.02 (0.75-1.38)	1.45 (1.21-1.75)	1.23 (0.92-1.66)
Model 2	1.27 (1.08-1.49)	1.34 (1.08-1.65)	1.23 (0.99-1.52)	1.03 (0.75-1.41)	1.31 (1.08-1.58)	1.07 (0.78-1.45)
CHD mortality						
Participants, No.	135	60	59	20	101	44
Model 1	1.22 (1.01-1.48)	1.10 (0.84-1.43)	1.09 (0.82-1.43)	0.68 (0.43-1.07)	1.66 (1.33-2.05)	1.65 (1.21-2.25)
Model 2	1.28 (1.05-1.55)	1.19 (0.91-1.56)	1.15 (0.87-1.52)	0.74 (0.47-1.17)	1.54 (1.24-1.93)	1.66 (1.21-2.27)
CVD mortality						
Participants, No.	237	125	94	45	199	100
Model 1	1.21 (1.05-1.40)	1.29 (1.07-1.55)	1.05 (0.84-1.31)	0.94 (0.69-1.27)	1.54 (1.33-1.80)	1.64 (1.34-2.02)
Model 2	1.25 (1.08-1.44)	1.36 (1.12-1.65)	1.09 (0.87-1.36)	0.99 (0.72-1.34)	1.41 (1.20-1.65)	1.54 (1.24-1.91)
All-cause mortality						
Participants, No.	705	325	299	142	603	271
Model 1	1.27 (1.17-1.37)	1.19 (1.06-1.34)	1.27 (1.12-1.43)	1.11 (0.93-1.32)	1.52 (1.39-1.65)	1.43 (1.26-1.62)
Model 2	1.28 (1.18-1.39)	1.21 (1.08-1.36)	1.29 (1.13-1.46)	1.13 (0.94-1.34)	1.46 (1.34-1.60)	1.39 (1.22-1.58)

Abbreviations: CHD, coronary heart disease; CVD, cardiovascular disease; HR, hazard ratio.

^a^ The reference group includes only participants who never used cigarettes and had no reported noncigarette tobacco product use for that column for both sole and exclusive analysis.

^b^ Model 1 adjusted for age, sex, race and ethnicity, education status, history of CHD, and cohort. Model 2 adjusted for age, sex, race and ethnicity, education status, history of CHD, cohort, body mass index (calculated as weight in kilograms divided by height in meters squared), hypertension, diabetes, antihypertensive medication, lipid-lowering medication, systolic blood pressure, diastolic blood pressure, alcohol use, and hyperlipidemia.

^c^ Sole use was defined as current noncigarette tobacco use without current cigarette use.

^d^ Exclusive use was defined as current noncigarette tobacco use without any history of cigarette use.

For sole pipe users, our findings indicated a significant increase in risk for myocardial infarction (HR, 1.43; 95% CI, 1.17-1.74), CHD (HR, 1.26; 95% CI, 1.05-1.52), and heart failure (HR, 1.42; 95% CI, 1.13-1.77) compared with those who never smoked pipes or cigarettes. However, exclusive pipe use demonstrated positive associations only with myocardial infarction compared with those who never smoked pipes or cigarettes (HR, 1.30; 95% CI, 0.99-1.70) (Table 4).

Lastly, sole use of smokeless tobacco was associated with CVD (HR, 1.34; 95% CI, 1.19-1.50), myocardial infarction (HR, 1.43; 95% CI, 1.20-1.70), CHD (HR, 1.41; 95% CI, 1.21-1.64), heart failure (HR, 1.41; 95% CI, 1.22-1.64), cardiovascular mortality (HR, 1.41; 95% CI, 1.20-1.65), and all-cause mortality (HR, 1.46; 95% CI, 1.34-1.60) compared with never using smokeless tobacco or cigarettes. For exclusive smokeless tobacco users, models revealed the highest increase in risks for CHD mortality (HR, 1.66; 95% CI, 1.21-2.27), CVD mortality (HR, 1.54; 95% CI, 1.24-1.91), heart failure (HR, 1.70; 95% CI, 1.40-2.06), and CVD (HR, 1.34; 95% CI, 1.13-1.59) compared with those who never used smokeless tobacco or cigarettes; there were also associations for CHD (HR, 1.36; 95% CI, 1.08-1.70) and all-cause mortality (HR, 1.39; 95% CI, 1.22-1.58) (Table 4).

In our sensitivity analysis, the incorporation of a shared frailty component for individual cohorts within our Cox model yielded results that were consistent with our primary findings (eTables 7 and 8 in Supplement 1). Moreover, repeating the analysis using participant age as the time scale revealed generally similar results (eTables 9 and 10 in Supplement 1). We further clarified our findings through a random-effects meta-analysis at the cohort level. This more detailed analysis largely confirmed results of our main study, revealing modest variability in outcomes across individual cohorts (eAppendix in Supplement 1). All results were similar in the sensitivity analysis of the nonimputed dataset (eTables 11 and 12 in Supplement 1).

## Discussion

In the most comprehensive study of this topic to date to our knowledge, this cohort study examined the association between cigar, pipe, and smokeless tobacco use and cardiovascular outcomes. Cigar use, including sole and exclusive use, was associated with multiple study outcomes, with a particularly large increase in risk for stroke. For overall pipe use, an association was found only for heart failure, while sole pipe use was associated with several study outcomes and exclusive pipe use was associated only with myocardial infarction. Smokeless tobacco use displayed positive associations with almost all study outcomes, a finding that persisted in the evaluation of sole and exclusive users. Taken together, these results are valuable in evaluating the risk continuum across tobacco products and may have implications for the regulation of noncigarette tobacco products by federal and international regulatory authorities.

The largest study to date on risks associated with exclusive cigar and pipe use was conducted by Christensen et al^23^ as part of the National Longitudinal Mortality Study. Importantly, their research spanned a vast sample of 357 420 individuals between 1985 and 2011, with total and cause-specific mortality as the study outcomes. Associations between exclusive cigar and pipe use and all-cause mortality compared with never using tobacco in our study align closely with findings of Christensen et al.^23^ For exclusive cigar use, our study reported an HR of 1.21 with a 95% CI of 1.08 to 1.36, which is comparable to the reported HR of 1.20 and 95% CI of 1.03 to 1.38 in Christensen et al.^23^ For pipe use, our study and that of Christensen et al^23^ found that all-cause mortality risk was increased but that this increase was not significant. Results of both studies collectively support the notion that current use of certain noncigarette tobacco products is associated with an increased risk of all-cause mortality. Our study adds to these data by additionally evaluating smokeless tobacco and including multiple nonfatal cardiovascular outcomes. Additionally, in contrast to the study by Christensen et al,^23^ our study was able to account for the confounding association of multiple known cardiovascular risk factors in observed associations and therefore arrive at more independent associations of noncigarette tobacco products with cardiovascular outcomes.

In comparison with the Christensen et al study,^23^ which examined only exclusive use, we considered 3 definitions of noncigarette tobacco product use: current, sole, and exclusive use. The first reason for this was practical: analyses of exclusive use have the least statistical power, while broad consideration of current noncigarette tobacco product use (adjusted for combustible cigarette status) preserves maximal statistical power. Second, we submit that consideration of each definition better places noncigarette tobacco product use in the general population context of well-known polyuse patterns of tobacco products. While the exclusive use analysis can be conducted to demonstrate a true causal point of view, for the most comprehensive understanding of cardiovascular risks associated with noncigarette tobacco product use, we have also accounted for incremental risks associated with potential dual use (current use analysis adjusted for combustible cigarette smoking), as well as noncigarette tobacco product use in the context of potential former combustible cigarette smoking (sole use analysis).

Although our analysis should not be considered a strict comparative risk assessment across tobacco products, it does facilitate broad comparisons between risks associated with combustible cigarettes vs noncigarette tobacco products. Overall, these findings indicate more pronounced increases in risk and more consistent associations of combustible cigarettes across all 9 outcomes, which is consistent with previous literature.^24,25,26,27^ In contrast, associations of noncigarette tobacco products were more variable and had generally lower increases in risk. However, an important limitation is that we were not able to account for the intensity of use across all product types. It is possible that the higher risks of adverse outcomes associated with combustible cigarette use may be due to higher risk intensity or differences in other patterns of use.^11^ Thus, our analysis should not be viewed as a comparison of use-adjusted toxic effects across tobacco product types but rather as cardiovascular risks within the context of how these products were most typically used in the population.

It should be noted that while we observed generally consistent results across all cohorts for combustible cigarette smoking, we observed considerable variability in individual point estimates of risk across individual cohorts for noncigarette tobacco products. We submit that this may be attributed mainly to the small sample sizes of users of noncigarette tobacco products in any 1 individual cohort. However, we cannot exclude other explanations, including cohort associations or differences in the type or brand of products used given that the CCC-Tobacco dataset incorporates data over many decades, from 1948 to 2015.

We believe that our data have distinct value for the regulation of tobacco products. The US FDA and other regulatory agencies have placed high importance on scientific evidence in the health associations of tobacco products. Findings of this study may support evidence-based regulatory actions that rely on or are predicated on a quantitative understanding of long-term cardiovascular harms associated with the relevant tobacco products. Combustible cigarettes and other tobacco products that were on the market before the 2016 Deeming Rule do not require FDA permission for marketing. However, the FDA has the ability to regulate new cigars, pipes, and smokeless tobacco products. There are limited health data, however, particularly related to cardiovascular disease risk to inform the regulation of different tobacco products and their specific constituents.^28^ Our study therefore provides new evidence to assess the continuum of risk across different tobacco products, as well as an epidemiologic analytic framework to support comparative studies of new and emerging tobacco products and their constituents and ingredients.

### Strengths and Limitations

Our study has several strengths. The CCC-Tobacco study represents a unique harmonization of the most thoroughly phenotyped, primarily US-based cardiovascular and aging cohorts ever conducted, to our knowledge. The extended follow-up time of up to 74 years significantly enhanced our ability to reveal even small associations. We are among the first studies to investigate the association of noncigarette tobacco products with heart failure and atrial fibrillation. Furthermore, the study population is diverse, with females constituting approximately half of participants. Notably, the sample included 3.4% American Indian or Alaska Native, 30.8% Black or African American, and 2.7% Hispanic or Latino participants. Finally, given the detailed risk factor data available in nearly all CCC-Tobacco cohorts, we were able to consider most confounders, enhancing our ability to provide adjusted risk.

There are also several limitations to consider. Despite large sample sizes in CCC-Tobacco, we still had limited statistical power for several noncigarette tobacco products, most prominently with pipe use. Given the observational study design, we cannot exclude residual confounding and can make no causal conclusions regarding these data. We did not have the power to specifically study less common use patterns, for example, dedicated analyses restricted to dual use or polyuse categories. Given the modest numbers of individuals using noncigarette tobacco products, we also lacked the power to examine subgroups by age, sex, or race and ethnicity. Additionally, we considered only baseline tobacco use data, and these habits may have changed during follow-up. In this study, we did not have information on specific subtypes of noncigarette tobacco products, which may have resulted in variations in outcomes that could not be accounted for in the analysis. Definitions of sole and exclusive use for noncigarette tobacco products did not account for the concurrent use of other noncigarette tobacco products, which may affect the interpretation of the results. Considering the higher prevalence of cigarette smoking (26.3%) in this sample compared with the current national estimate (11.5%),^29^ we recognize that this discrepancy may limit the generalizability of our findings. However, noncigarette tobacco use in our study was aligned with current national estimates. We examined data collected over decades but were unable to examine secular trends in the association between tobacco products and CVD outcomes. Future work in CCC-Tobacco will seek to harmonize tobacco use data across all visits for all cohorts, which will enable the study of product transitions, including cessation. We conducted analyses with multiple exposures and 9 cardiovascular and mortality outcomes, and we did not account for multiple statistical testing.

## Conclusions

In this harmonized multicohort study, we reported the largest study to date, to our knowledge, of cigars, pipes, and smokeless tobacco vs combustible cigarettes and the association with incident cardiovascular disease. Findings of our study support the view that the use of noncigarette tobacco products is associated with substantial harm, particularly from a cardiovascular point of view, although associations across specific products and specific outcomes were variable, with the smallest risk increases observed for pipes. Epidemiological insights from this study may support regulatory action relevant to new tobacco products or product standards and provide a framework for the future study of new and emerging tobacco products.

## Notes

Supplement 1.
eMethods.
**eTable 1. **Baseline characteristics across 14 cohorts in the Cross-Cohort Collaboration-Tobacco dataset with data on at least 1 noncigarette tobacco product **eTable 2.** Tobacco questionnaire form across cohorts **eTable 3. **Prevalence of different tobacco use products in each participating cohort **eTable 4. **Events number/total observation of each outcome across cohorts **eTable 5.** Outcome follow-up time in each cohort **eTable 6. **Total number of individuals and events included in each analysis **eTable 7.** Association between current use of traditional and noncigarette tobacco products and health outcomes compared with nonuse of given tobacco product accounting for heterogeneity between cohorts **eTable 8. **Association between sole and exclusive use of noncigarette tobacco products and health outcomes compared with nonuse of given tobacco product accounting for heterogeneity between cohorts **eTable 9.** Association between current use of traditional and noncigarette tobacco products and health outcomes compared with nonuse of given tobacco product considering participant age as time scale in survival analysis **eTable 10. **Association between sole and exclusive use of noncigarette tobacco products and health outcomes compared with nonuse of given tobacco product considering participant age as time scale in survival analysis **eTable 11.** Association between current use of traditional and noncigarette tobacco products and health outcomes compared with nonuse of given tobacco product based on nonimputed data in survival analysis models **eTable 12. **Association between sole and exclusive use of noncigarette tobacco products and health outcomes compared with nonuse of given tobacco product including frailty term in survival analysis models **eFigure 1. **Prevalence of cigarette and noncigarette tobacco use status **eFigure 2. **Conceptual illustration of patterns of cigar use based on use of combustible cigarette **eFigure 3. **Flowchart for sole and exclusive use of noncigarette tobacco products **eAppendix. **Meta-analysis of each association based on individual cohorts

Supplement 2.
Data Sharing Statement
