# Supplementary material for: Cigar, Pipe, and Smokeless Tobacco Use and Cardiovascular Outcomes From Cross Cohort Collaboration
Source: JAMA Netw Open. 2025 Jan 13;8(1):e2453987. doi: 10.1001/jamanetworkopen.2024.53987 (PMC11731180; doi:10.1001/jamanetworkopen.2024.53987)
Supplement: Supplement 1. — eMethods. eTable 1. Baseline characteristics across 14 cohorts in the Cross-Cohort Collaboration-Tobacco dataset with data on at least 1 noncigarette tobacco product eTable 2. Tobacco questionnaire form across cohorts eTable 3. Prevalence of different tobacco use products in each participating cohort eTable 4. Events number/total observation of each outcome across cohorts eTable 5. Outcome follow-up time in each cohort eTable 6. Total number of individuals and events included in each analysis eTable 7. Association between current use of traditional and noncigarette tobacco products and health outcomes compared with nonuse of given tobacco product accounting for heterogeneity between cohorts eTable 8. Association between sole and exclusive use of noncigarette tobacco products and health outcomes compared with nonuse of given tobacco product accounting for heterogeneity between cohorts eTable 9. Association between current use of traditional and noncigarette tobacco products and health outcomes compared with nonuse of given tobacco product considering participant age as time scale in survival analysis eTable 10. Association between sole and exclusive use of noncigarette tobacco products and health outcomes compared with nonuse of given tobacco product considering participant age as time scale in survival analysis eTable 11. Association between current use of traditional and noncigarette tobacco products and health outcomes compared with nonuse of given tobacco product based on nonimputed data in survival analysis models eTable 12. Association between sole and exclusive use of noncigarette tobacco products and health outcomes compared with nonuse of given tobacco product including frailty term in survival analysis models eFigure 1. Prevalence of cigarette and noncigarette tobacco use status eFigure 2. Conceptual illustration of patterns of cigar use based on use of combustible cigarette eFigure 3. Flowchart for sole and exclusive use of noncigarette tobacco products eA [file jamanetwopen-e2453987-s001.pdf]

Supplemental Online Content

Tasdighi E, Yao Z, Jha KK, et al. Cigar, pipe, and smokeless tobacco use and cardiovascular outcomes from Cross Cohort Collaboration. *JAMA Netw Open*. 2025;8(1):e2453987. doi:10.1001/jamanetworkopen.2024.53987

eMethods.

eTable 1. Baseline characteristics across 14 cohorts in the Cross-Cohort Collaboration-Tobacco dataset with data on at least 1 noncigarette tobacco product

eTable 2. Tobacco questionnaire form across cohorts

eTable 3. Prevalence of different tobacco use products in each participating cohort

eTable 4. Events number/total observation of each outcome across cohorts

eTable 5. Outcome follow-up time in each cohort

eTable 6. Total number of individuals and events included in each analysis

eTable 7. Association between current use of traditional and noncigarette tobacco products and health outcomes compared with nonuse of given tobacco product accounting for heterogeneity between cohorts

eTable 8. Association between sole and exclusive use of noncigarette tobacco products and health outcomes compared with nonuse of given tobacco product accounting for heterogeneity between cohorts

eTable 9. Association between current use of traditional and noncigarette tobacco products and health outcomes compared with nonuse of given tobacco product considering participant age as time scale in survival analysis

eTable 10. Association between sole and exclusive use of noncigarette tobacco products and health outcomes compared with nonuse of given tobacco product considering participant age as time scale in survival analysis

eTable 11. Association between current use of traditional and noncigarette tobacco products and health outcomes compared with nonuse of given tobacco product based on nonimputed data in survival analysis models

© 2025 Tasdighi E et al. *JAMA Network Open*.

eTable 12. Association between sole and exclusive use of noncigarette tobacco products and health outcomes compared with nonuse of given tobacco product including frailty term in survival analysis models

eFigure 1. Prevalence of cigarette and noncigarette tobacco use status

eFigure 2. Conceptual illustration of patterns of cigar use based on use of combustible cigarette

eFigure 3. Flowchart for sole and exclusive use of noncigarette tobacco products

eAppendix. Meta-analysis of each association based on individual cohorts

This supplemental material has been provided by the authors to give readers additional information about their work.

© 2025 Tasdighi E et al. *JAMA Network Open*.

Supplementary methods:

Section 1. Any cohort with data on either cigar, pipe, or smokeless tobacco use was considered as a participating cohort in this study. Data on electronic cigarettes and other tobacco products beyond cigarettes, cigars, pipes, and smokeless tobacco were unavailable in most cohorts and the inclusion and exclusion of cohorts selected were restricted to just the tobacco products named, and other tobacco products were not considered.

Section 2. The following 15 cohorts contributed to this report: 1) Atherosclerosis Risk in Communities (ARIC) Study, 2) Baltimore Longitudinal Study of Aging (BLSA), 3) Coronary Artery Risk Development in Young Adults (CARDIA) Study, 4) Chronic Renal Insufficiency Cohort (CRIC) 5) Dallas Heart Study (DHS) 6-8) Framingham Heart Study (FHS), original, offspring and third generation 9) Jackson Heart Study (JHS) 11) the Health, Aging and Body Composition Study (Health ABC) 11) Multi-Ethnic Study of Atherosclerosis (MESA), 12) Multiple Risk Factor Intervention Trial (MRFIT) 13) the Reasons for Geographic and Racial Differences in Stroke Study (REGARDS) 14) Rancho Bernardo Study (RBS) and 15) the Strong Heart Study (SHS). The ARIC study included 15,784 participants with a mean age of 54.1 years at Visit 1 (1987-1989). The BLSA followed 1,575 participants with a mean age of 64.6 years, starting at Visit 1 (1958). CARDIA enrolled 2,531 participants with a mean age of 30.1 years at Year 5 (1990-1991). CRIC had 5,561 participants with a mean age of 59.5 years at Visit 3 (2013-2015). DHS followed 3,557 participants with a mean age of 43.9 years, starting at DHS 1 Phase 1 (2003-2007). The FHS original cohort consisted of 3,753 participants with a mean age of 55.4 years at Exam 7 (1968-1971), while the FHS offspring cohort included 4,812 participants with a mean age of 36.8 years at Exam 1 (1971-1975), and the FHS third generation cohort had 4,063 participants with a mean age of 40.1 years at Exam 1 (2002-2005). Health ABC included 2,958 participants with a mean age of 73.6 years at year 1 (1997-1998). JHS followed 5,258 participants with a mean age of 54.8 years at Visit 1 (2000-2004). MESA enrolled 6,789 participants with a mean age of 62.1 years at Visit 1 (2000-2002). MRFIT included 12,866 participants with a mean age of 46.2 years at Visit 2 (1975-1976), while REGARDS had 30,174 participants with a mean age of 64.8 years at Visit 1 (2003-2007). Finally, RBS had 462 participants at Visit 4, and SHS included 3,485 participants at Phase 1 (1989-1991).

Funding statement for the participating cohorts:

The ARIC study (Atherosclerosis Risk in Communities) has been funded in whole or in part with federal funds from the National Heart, Lung, and Blood Institute (NHLBI), National Institutes of Health (NIH), Department of Health

and Human Services, under contract numbers HHSN268201700001I, HHSN268201700002I, HHSN268201700003I, HHSN268201700005I, and HHSN268201700004I.

BLSA was supported in part by the Intramural Research Program of the National Institute on Aging (BLSA protocol number: 03-AG-0325.)

The CARDIA study is supported by contracts HHSN268201800003I, HHSN268201800004I, HHSN268201800005I, HHSN268201800006I, and HHSN268201800007I from the National Heart, Lung, and Blood Institute (NHLBI).

Funding for the CRIC Study was obtained under a cooperative agreement from National Institute of Diabetes and Digestive and Kidney Diseases (U01DK060990, U01DK060984, U01DK061022, U01DK061021, U01DK061028, U01DK060980, U01DK060963, U01DK060902 and U24DK060990). In addition, this work was supported in part by: the Perelman School of Medicine at the University of Pennsylvania Clinical and Translational Science Award NIH/NCATS UL1TR000003, Johns Hopkins University UL1 TR-000424, University of Maryland GCRC M01 RR-16500, Clinical and Translational Science Collaborative of Cleveland, UL1TR000439from the National Center for Advancing Translational Sciences (NCATS) component of the National Institutes of Health and NIH roadmap for Medical Research, Michigan Institute for Clinical and Health Research (MICHR) UL1TR000433, University of Illinois at Chicago CTSA UL1RR029879, Tulane COBRE for Clinical and Translational Research in Cardiometabolic Diseases P20 GM109036, Kaiser Permanente NIH/NCRR UCSF-CTSI UL1 RR-024131, Department of Internal Medicine, University of New Mexico School of Medicine Albuquerque, NM R01DK119199. A portion of the data reported here have been supplied by the United States Renal Data System (USRDS). The interpretation and reporting of these data are the responsibility of the author(s) and in no way should be seen as an official policy or interpretation of the U.S. government.

The DHS was supported by grants from the Donald W. Reynolds Foundation and the National Center for Advancing Translational Sciences (UL1TR001105).

The Framingham Heart Study is supported by contracts NO1-HC-25195, HHSN268201500001I, and 75N92019D00031 from the National Heart, Lung and Blood Institute.

© 2025 Tasdighi E et al. *JAMA Network Open*.

The Jackson Heart Study (JHS) is supported and conducted in collaboration with Jackson State University (HHSN268201800013I), Tougaloo College (HHSN268201800014I), the Mississippi State Department of Health (HHSN268201800015I) and the University of Mississippi Medical Center (HHSN268201800010I, HHSN268201800011I and HHSN268201800012I) contracts from the National Heart, Lung, and Blood Institute (NHLBI) and the National Institute on Minority Health and Health Disparities (NIMHD). The authors also wish to thank the staffs and participants of the JHS.

The MESA study was supported by contracts 75N92020D00001, HHSN268201500003I, N01-HC-95159, 75N92020D00005, N01-HC-95160, 75N92020D00002, N01-HC-95161, 75N92020D00003, N01-HC-95162, 75N92020D00006, N01-HC-95163, 75N92020D00004, N01-HC-95164, 75N92020D00007, N01-HC-95165, N01-HC-95166, N01-HC-95167, N01-HC-95168 and N01-HC-95169 from the National Heart, Lung, and Blood Institute, and by grants UL1-TR-000040, UL1-TR-001079, and UL1-TR-001420 from the National Center for Advancing Translational Sciences (NCATS). The authors thank the other investigators, the staff, and the participants of the MESA study for their valuable contributions. A full list of participating MESA investigators and institutions can be found at http://www.mesa-nhlbi.org. This paper has been reviewed and approved by the MESA Publications and Presentations Committee.

The Multiple Risk Factor Intervention Trial was contracted by the National Heart, Lung, and Blood Institute (NHLBI), National Institutes of Health (NIH), Bethesda, MD. Follow-up after the end of the trial was supported with NIH/NHLBI grants R01-HL-43232 and R01-HL-68140.

This research project is supported by cooperative agreement U01 NS041588 co-funded by the National Institute of Neurological Disorders and Stroke (NINDS) and the National Institute on Aging (NIA), National Institutes of Health, Department of Health and Human Service. The content is solely the responsibility of the authors and does not necessarily represent the official views of the NINDS or the NIA. Representatives of the NINDS were involved in the review of the manuscript but were not directly involved in the collection, management, analysis or interpretation of the data. The authors thank the other investigators, the staff, and the participants of the REGARDS study for their valuable contributions. A full list of participating REGARDS investigators and institutions can be found at: https://www.uab.edu/soph/regardsstudy/

© 2025 Tasdighi E et al. *JAMA Network Open*.

The Rancho Bernardo Study was funded by research grants AG028507 and AG07181 from the National Institute on Aging and grant DK31801 from the National Institute of Diabetes and Digestive and Kidney Diseases.

The Strong Heart Study has been funded in whole or in part with federal funds from the National Heart, Lung, and Blood Institute, National Institute of Health, Department of Health and Human Services, under contract numbers 75N92019D00027, 75N92019D00028, 75N92019D00029, & 75N92019D00030. The study was previously supported by research grants: R01HL109315, R01HL109301, R01HL109284, R01HL109282, and R01HL109319 and by cooperative agreements: U01HL41642,U01HL41652, U01HL41654, U01HL65520, and U01HL65521.

Section 3. The "stats use" categories (i.e. current/former/never), including poly-tobacco users, reflect real-world usage patterns, which are important for public health and regulatory purposes. This breakdown aligns with the FDA's focus on understanding tobacco use as it occurs in practice. On the other hand, the "sole" and "exclusive" categories are more relevant for etiologic research, where the goal is to isolate the effects of individual tobacco products.

Section 4. Tobacco use status was defined separately for each product (cigarettes, cigars, pipes, and smokeless tobacco) as 'never,' 'former,' or 'current.' Due to the prevalence of poly-tobacco use, where individuals use more than one type of tobacco product simultaneously, the categories for use status are not mutually exclusive across products. For example, an individual who currently smokes cigarettes and uses smokeless tobacco would be categorized as a 'current user' for both products. Therefore, individuals may be counted in multiple 'current use' categories if they use more than one tobacco product type. This approach was taken to reflect the complexity of poly-tobacco use behaviors. As such, the denominators used in the calculation of these proportions are specific to each product, and users may appear in more than one category depending on their tobacco use patterns.

Section 5. To clarify, due to limited data on all tobacco products, the definitions of "sole" and "exclusive" use for each non-cigarette tobacco product were determined independently of the use of other non-cigarette tobacco products. In other words, "sole" and "exclusive" use were defined without accounting for whether individuals used additional non-cigarette tobacco products. For example, a sole cigar user might also be a current, former, or never user of pipe or smokeless tobacco products.

© 2025 Tasdighi E et al. *JAMA Network Open*.

Section 6. Data harmonization in CCC-Tobacco followed published best practices in the field and has been coded in a master file to enable replication. In the case of missing risk factor data in <10% of total participants, multiple imputation was conducted using the remaining non-missing risk factors. Multiple imputation was conducted using a multivariable model considering various factors.

Section 7. To evaluate the intra-group correlation within cohorts, we incorporated a shared frailty component into our Cox model, represented by the variable "cohort", which includes 15 unique cohort identifiers as a sensitivity analysis. This approach enhances the robustness of our inference by acknowledging and adjusting for the non-independence of survival times within cohorts, thereby providing a more accurate estimation of the association of covariates on survival. Moreover, a sensitivity analysis was conducted using the participant's age as the time scale. In this sensitivity analysis, Cox proportional hazards models were recalculated with age at baseline as Time 0 and age at event or censoring as the endpoint. This analysis was intended to confirm the consistency of risk estimates across different methodological frameworks and cohort characteristics.

© 2025 Tasdighi E et al. *JAMA Network Open*.

Table e1 supplementary. Baseline Characteristics Across the 14 Cohorts in the Cross-Cohort Collaboration-Tobacco Dataset with data on at least one non-cigarette tobacco products

	ARIC	BLSA	CARDIA	CRIC	DHS	FHS original	FHS off	FHS gen	Health ABC	JHS	MESA	MRIFT	REGARDS	RBS	SHS
**Sample size N (%)**	15784 (15.2)	1575 (1.5)	2531 (2.4)	5561 (5.4)	3557 (3.4)	3753 (3.6)	4812 (4.6)	4063 (3.9)	2958 (2.8)	5258 (5.1)	6789 (6.5)	12866 (12.4)	30174 (29.1)	462 (0.4)	3485 (3.4)
**Age**	54.1 ± 5.8	64.6 ± 14.8	30.1 ± 3.6	59.5 ± 10.8	43.9 ± 10.1	55.4 ± 8.4	36.8 ± 9.9	40.1 ± 8.8	73.6 ± 2.9	54.8 ± 12.8	62.1 ± 10.2	46.2 ± 6.0	64.8 ± 9.4	72.4 ± 8.7	56.4 ± 8.1
**Female N (%)**	8704 (55.1)	808 (51.3)	1335 (52.7)	2424 (43.6)	1986 (55.8)	2185 (58.2)	2499 (51.9)	2168 (53.4)	1580 (53.4)	3339 (63.5)	3588 (52.8)	0 (00.0)	16627 (55.1)	256 (56.1)	2039 (58.6)
**Race and ethnicity**															
**White**	11473 (72.7)	1088 (72.6)	1395 (55.1)	2249 (40.4)	1047 (29.4)	3753 (100)	4812 (100)	4063 (100)	1692 (57.2)	0	2615 (38.5)	11559 (89.8)	17666 (58.5)	462 (100)	0
**African American**	4263 (27.0)	378 (25.2)	1136 (44.9)	2391 (43.3)	1834 (51.6)	0	0	0	1266 (42.8)	5258 (100)	1877 (27.6)	931 (7.2)	12508 (41.4)	0	0
**Asian**	34 (0.2)	27 (1.8)	0	0	0	0	0	0	0	0	802 (11.8)	0	0	0	0
**Hispanic**	0	0	0	742 (13.0)	602 (16.9)	0	0	0	0	0	1496 (22.0)	0	0	0	0
**American Indian or Alaskan**	14 (0.1)	5 (0.3)	0	0	0	0	0	0	0	0	0	0	0	0	3485 (100)
**Other**	0	0	0	197(3.5)	74 (2.1)	0	0	0	0	0	0	376 (2.9)	0	0	0
**Education**															
**High School**	3767 (23.9)	14 (0.9)	155 (6.1)	1127 (20.3)	726 (20.4)	1509 (41.1)	303 (8.9)	22 (0.7)	756 (25.6)	958 (18.3)	1225 (18.0)	2083 (16.3)	3790 (12.6)	23 (5.0)	1469 (42.2)
**High school completed**	6412 (40.7)	112 (7.2)	1357 (53.7)	1025 (18.4)	1106 (31.1)	1136 (31.0)	1710 (50.3)	465 (13.7)	968 (32.8)	1056 (20.1)	1234 (18.2)	2685 (21.0)	7803 (25.9)	118 (25.8)	979 (28.1)
**College degree**	5586 (35.4)	1438 (91.9)	1014 (40.1)	3407 (61.3)	1724 (48.5)	1023 (27.9)	1384 (40.7)	2897 (85.6)	1226 (51.6)	3231 (61.6)	4329 (63.8)	8035 (62.8)	18557 (61.5)	316 (69.2)	1035 (29.7)
**Alcohol use N (%)**	8765 (55.8)	1320 (84.0)	2239 (88.9)	3426 (61.6)	2434 (68.6)	2660 (71.1)	4130 (86.6)	3366 (82.9)	1443 (48.8)	2401 (45.8)	3747 (55.4)	11897 (92.5)	11039 (37.3)	408 (90.5)	1389 (90.5)
**BMI kg/mm2**	27.7 ± 5.4	26.9 ± 4.7	25.9 ± 5.7	32.3 ± 7.6	29.6 ± 7.0	25.8 ± 4.2	25.2 ± 4.3	26.9 ± 5.6	27.4 ± 4.8	31.7 ± 7.2	28.3 ± 5.5	27.7 ± 3.5	29.3 ± 6.2	25.1 ± 3.8	30.5 ± 6.0
**Hypertension N (%)**	5508 (34.9)	579 (36.9)	115 (4.5)	5168 (92.9)	1166 (32.8)	1822 (48.6)	946 (19.7)	673 (16.6)	2031 (68.7)	3107 (59.2)	3283 (48.4)	11788 (91.6)	17851 (59.2)	288 (62.5)	1308 (37.8)
**Systolic BP mmHg**	121.3 ± 19.0	117.5 ± 15.5	107.7 ± 12.0	128.6 ± 21.4	124.9 ± 18.9	138.0 ± 22.9	122.1 ± 16.5	116.7 ± 14.1	135.9 ± 21.0	127.4 ± 16.9	126.6 ± 21.6	147.6 ± 15.3	127.6 ± 16.7	140.5 ± 22.2	126.6 ± 19.1
**Diastolic BP mmHg**	73.7 ± 11.3	66.2 ± 8.8	68.8 ± 10.5	71 .1± 12.6	78.3 ± 10.3	85.0 ± 11.6	78.7 ± 10.9	75.3 ± 9.6	71.3 ± 11.8	75.7 ± 8.7	71.9 ± 10.2	99.2 ± 7.7	76.5 ± 9.7	75.4 ± 9.3	76.4 ±10.2
**BP medication N (%)**	4002 (25.5)	529 (33.9)	36 (1.4)	5117 (92.1)	767 (22.1)	350 (9.4)	160 (3.4)	343 (8.5)	1609 (54.6)	2731 (52.4)	2523 (37.2)	2488 (19.3)	15548 (53.6)	151 (38.7)	384 (18.2)

© 2025 Tasdighi E et al. *JAMA Network Open*.

**Diabetes N (%)**	1561 (9.9)	238 (15.1)	47 (1.9)	2823 (51.4)	374 (10.6)	157 (4.2)	90 (2.0)	123 (3.0)	977 (33.0)	1228 (23.6)	859 (12.6)	711 (5.6)	6393 (22.0)	52 (11.3)	1439 (41.6)
**Dyslipidemia N (%)**	8983 (57.8)	755 (48.1)	797 (32.3)	4326 (86.5)	1846 (52.0)	2171 (60.0)	2206 (46.6)	1557 (38.4)	1559 (53.3)	2804 (57.7)	3778 (55.8)	10391 (80.8)	19162 (65.4)	268 (58.3)	2176 (63.5)
**HPL N (%)**	5769 (37.1)	232 (14.7)	256 (10.5)	1081 (27.9)	654 (18.4)	2163 (59.8)	1125 (23.8)	671 (16.5)	824 (28.2)	1111 (23.3)	1556 (23.0)	8970 (69.7)	6823 (23.6)	249 (54.1)	900 (26.3)
**HTG N (%)**	4268 (27.5)	225 (14.4)	224 (9.1)	1471 (37.9)	850 (23.9)	607 (37.7)	676 (14.3)	846 (20.8)	889 (30.4)	776 (16.1)	1984 (29.3)	6903 (53.7)	8026 (27.8)	160 (34.8)	1133 (33.1)
**HPL Medication N (%)**	448 (2.9)	445 (56.8)	7 (0.3)	3419 (61.9)	238 (6.9)	39 (1.0)	27 (0.6)	273 (6.7)	416 (14.1)	717 (13.8)	1098 (16.2)	159 (1.2)	10008 (33.5)	6 (1.5)	11 (0.52)
**LDL-C mg/dL**	137.6 ± 39.3	109.7 ± 32.2	107.6 ± 32.4	102.8 ± 35.6	106.3 ± 35.4	-	128.6 ± 37.2	111.7 ± 31.4	121.6 ± 34.8	126.6 ± 36.5	117.2 ± 31.4	160.0 ± 36.0	113.9 ± 34.8	149.3 ± 41.2	110.4 ± 31.9
**Total Cholesterol mg/dL**	214.9 ± 42.0	189.9 ± 36.4	178.0 ± 34.7	183.7 ± 45.6	180.3 ± 39.6	253.2 ± 48.7	200.2 ± 40.0	188.8 ± 35.5	202.9 ± 38.4	199.3 ± 40.0	194.2 ± 35.7	240.4 ± 36.8	192.0 ± 40.1	237.2 ± 44.2	195.4 ± 39.5
**HDL-C mg/dL**	51.6 ± 17.1	59.7 ± 17.0	53.1 ± 14.4	47.5 ± 15.5	49.8 ± 14.8	-	51.8 ± 16.2	54.3 ± 16.1	54.3 ± 17.0	51.8 ± 14.6	50.9 ± 14.8	42.1 ± 11.8	51.8 ± 16.1	60.8 ± 19.7	46.1 ± 13.8
**Triglyceride mg/dL**	110 (79 – 157)	88.5 (66 – 122)	64 (46 – 93.5)	129 (89 – 186)	96 (67 – 147)	132 (98 – 178)	81 (56 – 120)	92 (65 – 138)	118 (88 – 163.5)	90 (65 – 126)	111 (78 – 161)	158 (113 – 228)	111 (81 – 158)	99 (69 – 145)	119 (82 – 171)

Atherosclerosis Risk in Communities (ARIC) Study, 2) Baltimore Longitudinal Study of Aging (BLSA), 3) Coronary Artery Risk Development in Young Adults (CARDIA) Study, 4) Chronic Renal Insufficiency Cohort (CRIC) 5) Dallas Heart Study (DHS) 6-8) Framingham Heart Study (FHS), original, offspring and third generation 9) the Health, Aging and Body Composition Study (Health ABC) 10) Jackson Heart Study (JHS) 11) Multi-Ethnic Study of Atherosclerosis (MESA), 12) Multiple Risk Factor Intervention Trial (MRFIT) 13) the Reasons for Geographic and Racial Differences in Stroke Study (REGARDS) 14) Rancho Bernardo Study (RBS) 15) the Strong Heart Study (SHS) 15)

BMI: body mass index; BP: blood pressure; HPL: hyperlipidemia; HTG: hypertriglyceridemia; LDL-C: low density lipoprotein cholesterol; HDL-C: high density lipoprotein cholesterol

© 2025 Tasdighi E et al. *JAMA Network Open*.

Table e2 supplementary. Tobacco questionnaire form across the cohorts				
	Combustible cigarettes	Cigars	Pipe	Smokeless tobacco
ARIC	- Have you ever smoked cigarettes? (Code “No” if less than 400 cigarettes in a lifetime)<br>- Do you currently smoke cigarettes?	- Have you ever smoked cigars or cigarillos regularly?<br>- Do you now smoke cigars/cigarillos?	- Have you ever smoked a pipe regularly?<br>- Do you now smoke a pipe?	NA
BLSA	- Ever smoked 100 cigarettes<br>- Smokes cigarettes now	- Ever smoked 50 cigars<br>- Age began smoking cigars<br>- Smokes cigars now	- Ever smoke 3 packages pipe tobacco<br>- Smokes pipe now	NA
CARDIA	- Have you ever smoked cigarettes regularly for at least three months? By "regularly" we mean at least 5 cigarettes per week almost every week.<br>- Do you still smoke cigarettes regularly? By "regularly" we mean at least 5 cigarettes per week almost every week.	- Have you ever smoked cigars regularly for at least three months? By "regularly" we mean at least two cigars per week almost every week.	- Have you ever smoked a tobacco pipe regularly for at least three months? By "regularly" we mean at least two pipes full of tobacco a week almost every week.	- Have you regularly used smokeless tobacco such as snuff, chewing tobacco or other products such as "Skoal's Bandits" for at least three months? By "regularly" we mean at least once a week almost every week.
CRIC	- Have you smoked at least 100 cigarettes during your entire life?<br>- Do you smoke cigarettes now?	- Have you ever smoked at least 20 cigars in your entire life?<br>- Do you currently smoke cigars	NA	- Have you ever used chewing tobacco, snuff, or other smokeless tobacco products?<br>- Since your last CRIC clinic visit, did you use chewing tobacco, snuff, or other smokeless tobacco products?,Y/N
DHS	- Smoked at least 100 cigarettes in your lifetime<br>- Last 30 days, often have smoked cigarettes:	- Have you ever smoked cigars regularly<br>- Do you still smoke cigars regularly<br>- Do you still smoke cigars occasionally	- Have you ever smoked pipes regularly<br>- Do you still smoke pipes regularly<br>- Do you still smoke pipes occasionally	- Ever used any other tobacco products regularly<br>- Do you still use smokeless tobacco regularly<br>- Do you still use smokeless tobacco occasionally

© 2025 Tasdighi E et al. *JAMA Network Open*.

FHS (original, offspring and third generation)	- CIGARETTES REG LAST EXAM<br>- DO YOU NOW SMOKE	CIGARS:<br>- EVER SMOKED REGULARLY<br>- SMOKED REG IN LAST YEAR<br>- DO YOU SMOKE NOW	PIPES:<br>- EVER SMOKED REGULARLY<br>- SMOKED REG IN LAST YEAR	NA
JHS	- Have you smoked at least 400 cigarettes in your lifetime?<br>- Do you now smoke cigarettes?<br>- How long has it been since you last smoked cigarettes?<br>- Since you began smoking, for how many years were you off cigarettes?	- Have you ever used any other tobacco products regularly, that is cigars or cigarillos, pipes, chewing tobacco, or snuff/dip?<br>- Do you currently smoke cigars or cigarillos?	- Have you ever used any other tobacco products regularly, that is cigars or cigarillos, pipes, chewing tobacco, or snuff/dip?<br>- Currently smoke a pipe?	- Have you ever used any other tobacco products regularly, that is cigars or cigarillos, pipes, chewing tobacco, or snuff/dip?<br>- Currently use chewing Tobacco?<br>- Currently use dip or snuff?
Health ABC	- Have you ever smoked at least 100 cigarettes?<br>- Do you currently smoke cigarettes?	- Have you ever smoke a pipe/cigars?<br>- Do you smoke pipe/cigar now?	- Have you ever smoke a pipe/cigars?<br>- Do you smoke pipe/cigar now?	NA
MESA	- Have you ever smoked at least 100 cigarettes in your lifetime?<br>- How old were you when you first started smoking cigarettes?<br>- Have you smoked cigarettes during the last 30 days?	- Have you smoked more than 20 cigars in your life?<br>- Have you smoked cigars during the last 30 days?	- Have you smoked at least 20 pipeful of tobacco in your life?<br>- Have you smoked a pipe during the last 30 days?	- Have you used chewing tobacco, such as Redman, Levi Garret, or Beechnut, at least 20 times?<br>- Have you used chewing tobacco during the last 30 days?
MRFIT	-	- Have you ever smoke a pipe/cigars?<br>- Do you smoke pipe/cigar now?	- Have you ever smoke a pipe/cigars?<br>- Do you smoke pipe/cigar now?	NA
REGARDS	- Have you smoked at least 100 cigarettes in lifetime?<br>- Do you smoke cigarettes now, even occasionally?	- Currently smoke cigars, cigarillos or a pipe, even occasionally?	- Currently smoke cigars, cigarillos or a pipe, even occasionally?	- Ever used or tried any smokeless tobacco products such as chewing tobacco or snuff?<br>- Currently use chewing tobacco or snuff?

© 2025 Tasdighi E et al. *JAMA Network Open*.

RBS V4	- Have you ever smoked cigarettes?<br>- Do you smoke cigarettes currently?	- EVER SMOKED PIPE OR CIGAR MORE THAN ONE YEAR?<br><br>- PIPE/CIGAR SMOKER	- EVER SMOKED PIPE OR CIGAR MORE THAN ONE YEAR?<br><br>- PIPE/CIGAR SMOKER	NA
SHS	- Have you smoked at least 100 cigarettes in your entire life?<br>How old were you when you first started smoking cigarettes fairly regularly?<br>- Do you smoke cigarettes now?	- Do you smoke cigars NOW?	- Do you smoke a pipe NOW?	- Do you use chewing tobacco/snuff NOW ?
Atherosclerosis Risk in Communities (ARIC) Study, 2) Baltimore Longitudinal Study of Aging (BLSA), 3) Coronary Artery Risk Development in Young Adults (CARDIA) Study, 4) Chronic Renal Insufficiency Cohort (CRIC) 5) Dallas Heart Study (DHS) 6-8) Framingham Heart Study (FHS), original, offspring and third generation 9) the Health, Aging and Body Composition Study (Health ABC) 10) Jackson Heart Study (JHS) 11) Multi-Ethnic Study of Atherosclerosis (MESA), 12) Multiple Risk Factor Intervention Trial (MRFIT) 13) the Reasons for Geographic and Racial Differences in Stroke Study (REGARDS) 14) Rancho Bernardo Study (RBS) 15) the Strong Heart Study (SHS)				

© 2025 Tasdighi E et al. *JAMA Network Open*.

Table e3 supplementary. Prevalence of different tobacco use products in each participating cohort

Cohorts	Cigarette use	Cigar Use	Pipe Use	Smokeless Use
ARIC	4132 (26.18%)	290 (1.84%)	271 (1.72%)	532 (3.37%)
BLSA	42 (2.67%)	30 (1.90%)	5 (0.32%)	0 (0.00%)
CARDIA	1243 (48.98%)	16 (0.63%)	5 (0.20%)	118 (4.65%)
CRIC	707 (12.71%)	145 (2.61%)	0 (0.00%)	35 (0.63%)
DHS	1036 (29.13%)	123 (3.46%)	15 (0.42%)	60 (1.69%)
FHS, original	1741 (46.39%)	190 (5.06%)	152 (4.05%)	0 (0.00%)
FHS, offspring	2175 (45.20%)	211 (4.38%)	174 (3.62%)	0 (0.00%)
FHS, 3rd Generation	629 (15.48%)	46 (1.13%)	0 (0.00%)	0 (0.00%)
Health ABC	311 (10.51%)	76 (2.57%)	0 (0.00%)	0 (0.00%)
JHS	685 (13.03%)	63 (1.20%)	18 (0.34%)	88 (1.67%)
MESA	886 (13.05%)	131 (1.93%)	42 (0.62%)	44 (0.65%)
MRFIT	7608 (59.13%)	2813 (21.86%)	1573 (12.23%)	0 (0.00%)
REGARDS	41 (8.87%)	16 (3.46%)	13 (2.81%)	0 (0.00%)
RBS	4395 (14.56%)	1051 (3.48%)	0 (0.00%)	640 (2.12%)
SHS	1331 (38.19%)	51 (1.46%)	49 (1.41%)	52 (1.49%)

Atherosclerosis Risk in Communities (ARIC) Study, 2) Baltimore Longitudinal Study of Aging (BLSA), 3) Coronary Artery Risk Development in Young Adults (CARDIA) Study, 4) Chronic Renal Insufficiency Cohort (CRIC) 5) Dallas Heart Study (DHS) 6-8) Framingham Heart Study (FHS), original, offspring and third generation 9) the Health, Aging and Body Composition Study (Health ABC) 10) Jackson Heart Study (JHS) 11) Multi-Ethnic Study of Atherosclerosis (MESA), 12) Multiple Risk Factor Intervention Trial (MRFIT) 13) the Reasons for Geographic and Racial Differences in Stroke Study (REGARDS) 14) Rancho Bernardo Study (RBS) 15) the Strong Heart Study (SHS)

© 2025 Tasdighi E et al. *JAMA Network Open*.

Table 4 supplementary. Evens number/total observation of each outcome across cohorts									
**Cohorts (Events/observation)**	**MI**	**Stroke**	**Heart failure**	**Atrial fibrillation**	**CVD**	**CHD**	**CHD mortality**	**CVD mortality**	**All-cause mortality**
**ARIC**	2187/15784	1615/15784	2777/15784	4846/15784	4160/15784	3253/15784	1151/15784	2513/15784	8033/15784
**BLSA**	45/1575	55/1575	45/1575	91/1575	NA	NA	NA	NA	376/1575
**CARDIA**	67/2538	66/2538	77/2538	171/2538	47/2538	25/2538	13/2538	64/2538	259/2538
**CRIC**	647/5561	315/5561	935/5561	1182/5561	1122/5561	906/5561	402/5561	469/5561	1871/5561
**DHS**	76/3557	74/3557	96/3557	186/3557	71/3557	47/3557	35/3557	77/3557	239/3557
**FHS, original**	1148/3753	699/3753	1435/3753	2081/3753	876/3753	915/3753	724/3753	1314/3753	3741/3753
**FHS, offspring**	932/4812	419/4812	1008/4812	1365/4812	561/4812	970/4812	256/4812	516/4812	2165/4812
**FHS, 3rd Generation**	104/4063	35/4063	81/4063	117/4063	21/4063	79/4063	11/4063	16/4063	97/4063
**Health ABC**	263/2958	331/2958	623/2958	951/2958	NA	NA	431/2958	503/2958	1905/2958
**JHS**	286/5258	231/5258	344/5258	482/5258	366/5258	NA	134/5258	NA	1358/5258
**MESA**	359/6789	356/6789	510/6789	935/6789	414/6789	988/6789	199/6789	411/6789	1702/6789
**MRFIT**	681/12866	108/12866	841/12866	1023/12866	NA	NA	428/12866	556/12866	1033/12866
**REGARDS**	22/462	21/462	92/462	203/462	15/462	17/462	72/462	176/462	421/462
**RBS**	2140/30181	1744/30181	2922/30181	5597/30181	2256/30181	1121/30181	1124/30181	2913/30181	9826/30181
**SHS**	515/3485	361/3485	942/3485	1261/3485	550/3485	0/3485	609/3485	801/3485	2454/3485

Atherosclerosis Risk in Communities (ARIC) Study, 2) Baltimore Longitudinal Study of Aging (BLSA), 3) Coronary Artery Risk Development in Young Adults (CARDIA) Study, 4) Chronic Renal Insufficiency Cohort (CRIC) 5) Dallas Heart Study (DHS) 6-8) Framingham Heart Study (FHS), original, offspring and third generation 9) the Health, Aging and Body Composition Study (Health ABC) 10) Jackson Heart Study (JHS) 11) Multi-Ethnic Study of Atherosclerosis (MESA), 12) Multiple Risk Factor Intervention Trial (MRFIT) 13) the Reasons for Geographic and Racial Differences in Stroke Study (REGARDS) 14) Rancho Bernardo Study (RBS) 15) the Strong Heart Study (SHS)
MI: myocardial infarction; CHD: coronary heart disease; CVD: cardiovascular disease

© 2025 Tasdighi E et al. *JAMA Network Open*.

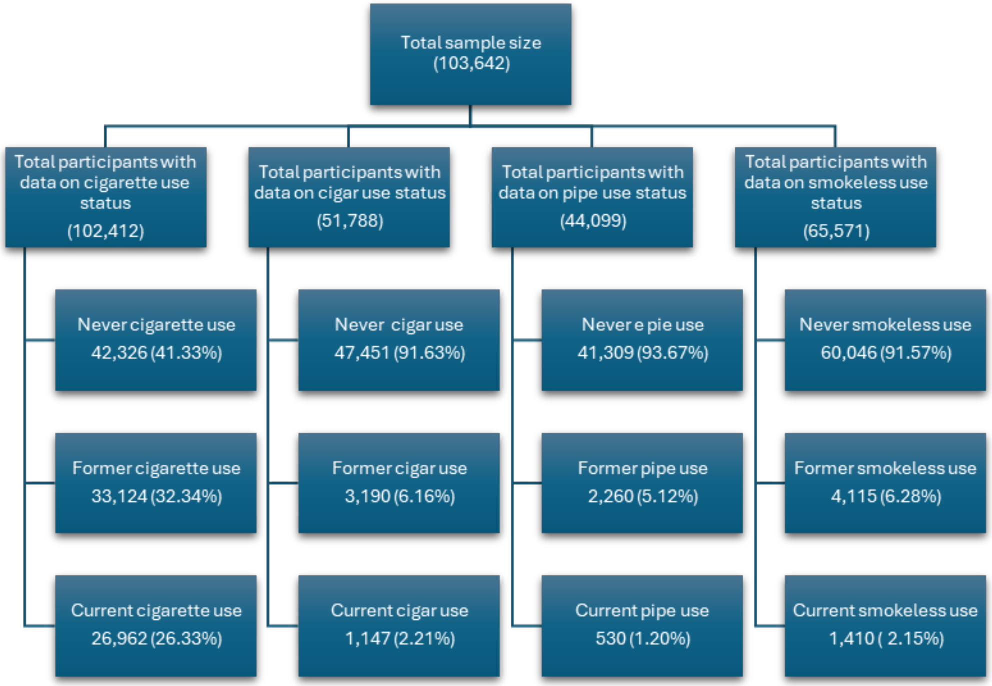

Figure 1 supplementary. The prevalence of cigarette and non-cigarette tobacco use status.

© 2025 Tasdighi E et al. *JAMA Network Open*.

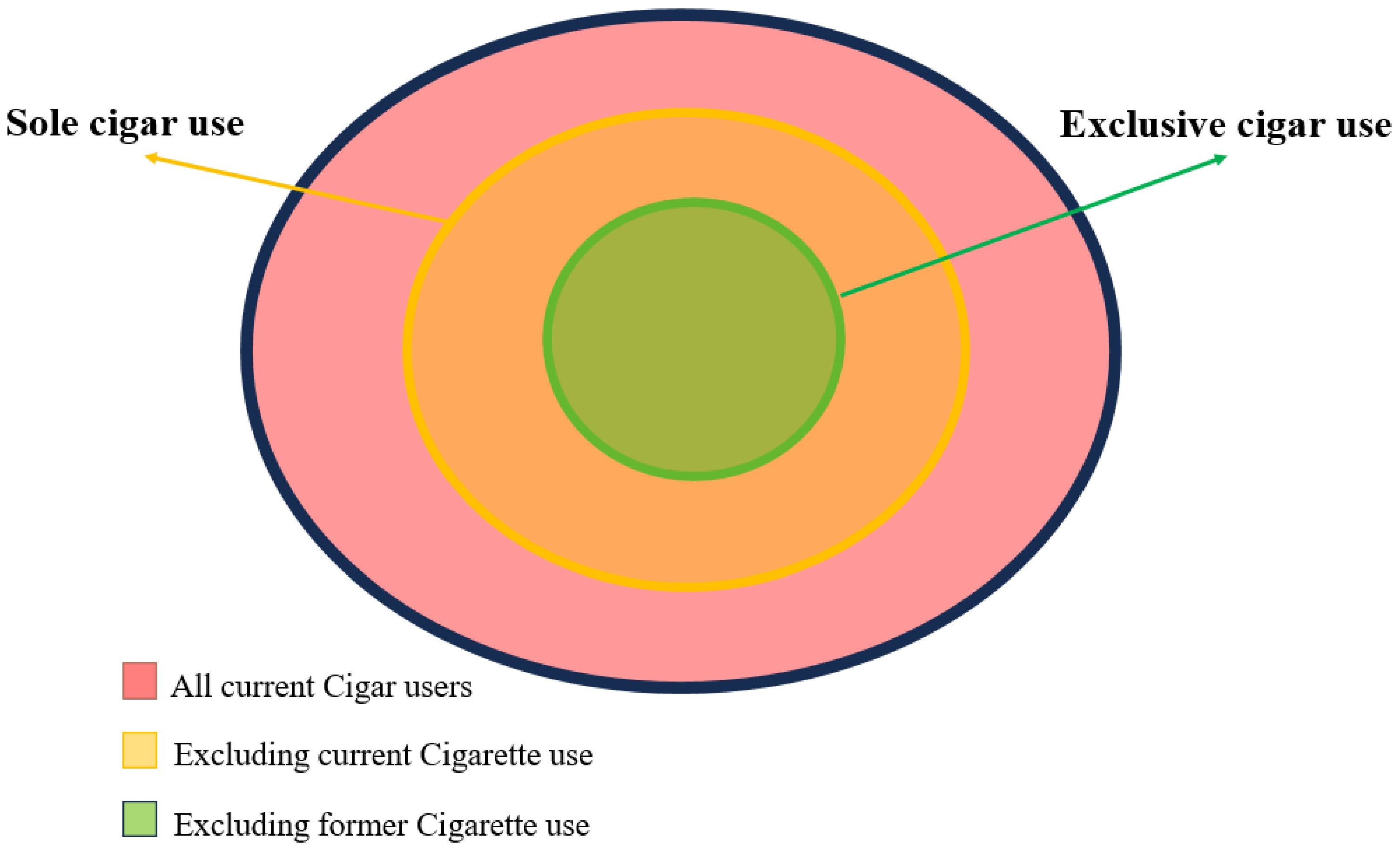

figure e2 supplementary. Conceptual illustration of the different patterns of cigar use based on the use of combustible cigarette

© 2025 Tasdighi E et al. *JAMA Network Open*.

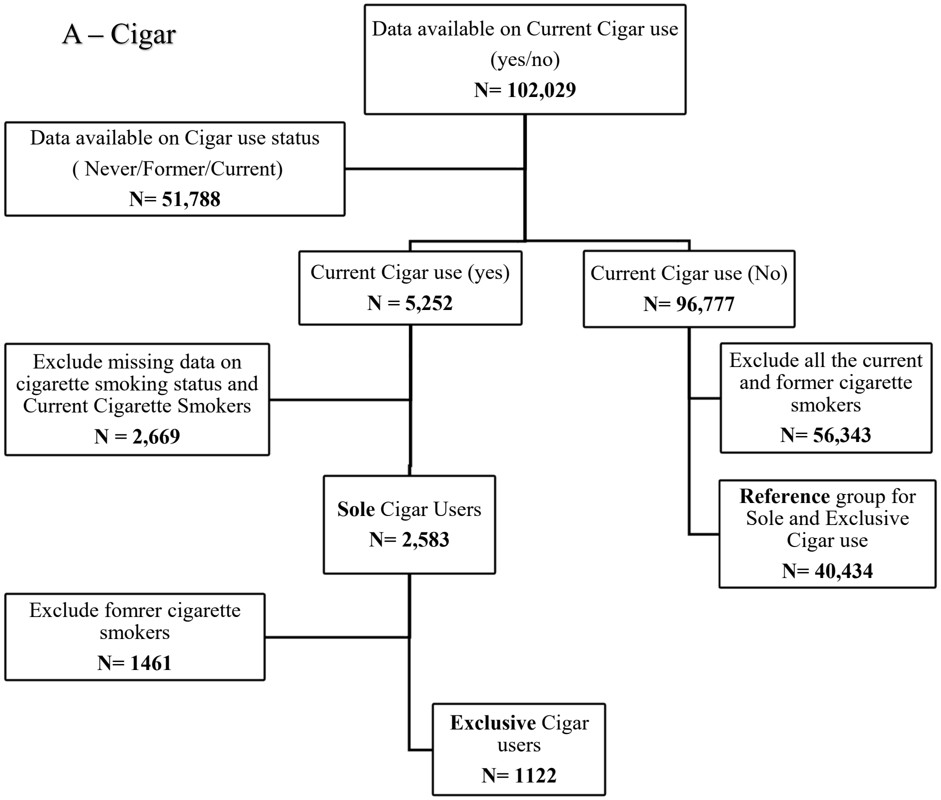

© 2025 Tasdighi E et al. *JAMA Network Open*.

B – Pipe

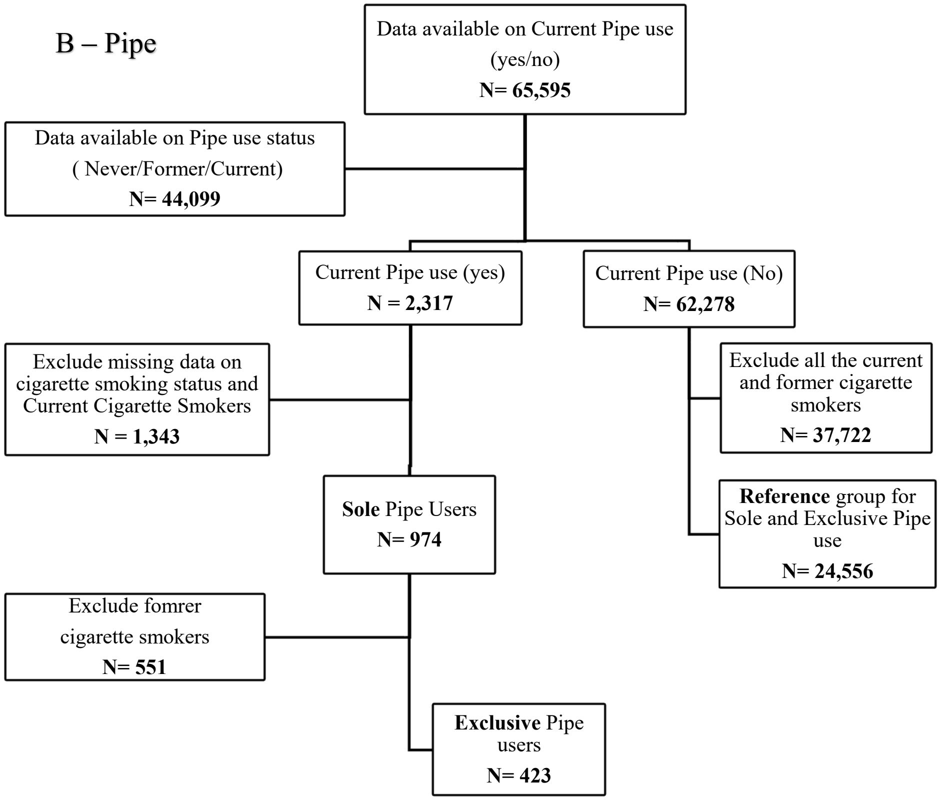

© 2025 Tasdighi E et al. *JAMA Network Open*.

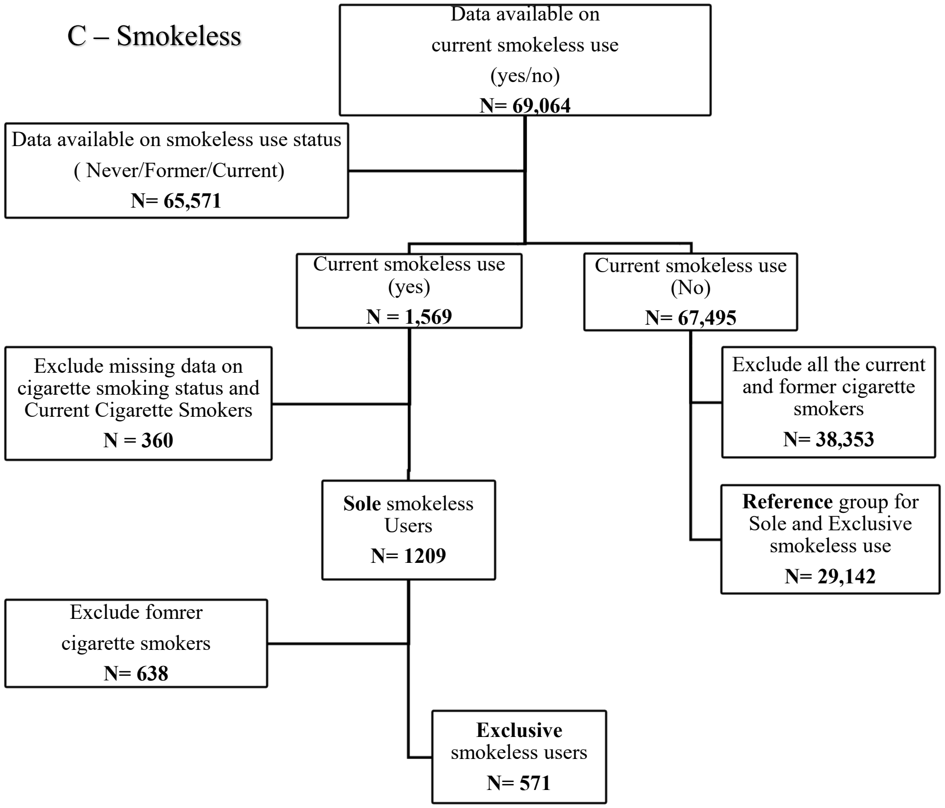

Figure e3 supplementary. Flow chart for the sole and exclusive use of non-cigarette tobacco products.

© 2025 Tasdighi E et al. *JAMA Network Open*.

Table e5 supplement. Outcomes follow up time in each cohort.

			Follow Up time in CCC-Tobacco								
Cohorts	**Baseline visit for the CCC- Tobacco**	**Baseline date for the Cox models**	**MI**	**Stroke**	**Heart failure**	**Atrial fibrillation**	**CVD**	**CHD**	**CHD mortality**	**CVD mortality**	**All-cause mortality**
ARIC	Visit 1	26 (16 - 30)	27 (17 - 31)	26 (16 - 30)	25 (15 - 30)	26 (16 - 30)	25 (16 - 29)	28 (18 - 31)	28 (18 - 31)	28 (18 - 31)	26 (16 - 30)
BLSA	Visit 1	9 (5 - 12)	9 (5 - 12)	9 (5 - 12)	9 (5 - 12)	NA	NA	NA	NA	10 (6 - 13)	9 (5 - 12)
CARDIA	Year 5	27 (27 - 27)	27 (27 - 27)	27 (27 - 27)	27 (27 - 27)	27 (27 - 27)	25 (20 - 25)	27 (27 - 27)	27 (27 - 27)	27 (27 - 27)	27 (27 - 27)
CRIC	Visit 3	7 (5 - 13)	7 (5 - 13)	7 (5 - 14)	7 (5 - 14)	6 (4 - 13)	6 (3 - 11)	8 (5 - 14)	8 (5 - 14)	8 (5 - 14)	7 (5 - 13)
DHS	DHS 1 Phase 1	15 (14 - 15)	15 (14 - 15)	20 (19 - 20)	20 (19 - 20)	15 (14 - 15)	15 (14 - 15)	20 (20 - 20)	20 (20 - 20)	20 (19 - 20)	15 (14 - 15)
FHS, original	Exam 7	22 (12 - 32)	24 (14 - 34)	21 (11 - 31)	20 (10 - 30)	24 (14 - 34)	23 (14 - 33)	24 (15 - 34)	24 (15 - 34)	24 (15 - 34)	22 (12 - 32)
FHS, offspring	Exam 1	42 (28 - 45)	42 (32 - 45)	42 (28 - 45)	41 (28 - 45)	42 (32 - 45)	40 (26 - 44)	44 (34 - 46)	44 (34 - 46)	44 (34 - 46)	42 (28 - 45)
FHS, 3rd Generation	Exam 1	15 (14 - 16)	15 (13 - 16)	15 (14 - 16)	15 (14 - 16)	15 (13 - 16)	14 (13 - 15)	15 (14 - 16)	15 (14 - 16)	15 (14 - 16)	15 (14 - 16)
Health ABC	Year 1	13 (8 - 13)	13 (8 - 13)	13 (8 - 13)	12 (7 - 13)	NA	NA	13 (8 - 13)	13 (8 - 13)	13 (8 - 13)	13 (8 - 13)
JHS	Visit 1	14 (13 - 15)	14 (12 - 15)	14 (13 - 15)	14 (11 - 15)	12 (11 - 12)	NA	17 (16 - 18)	NA	17 (16 - 18)	14 (13 - 15)
MESA	Visit 1	17 (12 - 17)	17 (12 - 17)	17 (14 - 18)	17 (13 - 18)	17 (12 - 17)	14 (10 - 15)	17 (15 - 18)	17 (15 - 18)	17 (15 - 18)	17 (12 - 17)
MRFIT	Visit 2	6 (6 - 6)	6 (6 - 6)	11 (10 - 11)	11 (10 - 11)	NA	NA	11 (10 - 11)	11 (10 - 11)	11 (10 - 11)	6 (6 - 6)
REGARDS	Visit 1	8 (4 - 12)	8 (4 - 13)	13 (7 - 19)	12 (7 - 19)	8 (4 - 12)	13 (12 - 22)	13 (7 - 20)	13 (7 - 20)	13 (7 - 20)	8 (4 - 12)
RBS	Visit 4	11 (6 - 14)	12 (6 - 15)	11 (6 - 14)	11 (6 - 15)	12 (7 - 15)	9 (8 - 10)	11 (7 - 14)	11 (7 - 14)	11 (7 - 14)	11 (6 - 14)
SHS	Phase 1	30 (29 - 31)	30 (29 - 31)	30 (26 - 31)	29 (17 - 30)	30 (29 - 31)	NA	20 (10 - 29)	20 (10 - 29)	20 (10 - 29)	30 (29 - 31)

NA; Not available
Atherosclerosis Risk in Communities (ARIC) Study, 2) Baltimore Longitudinal Study of Aging (BLSA), 3) Coronary Artery Risk Development in Young Adults (CARDIA) Study, 4) Chronic Renal Insufficiency Cohort (CRIC) 5) Dallas Heart Study (DHS) 6-8) Framingham Heart Study (FHS), original, offspring and third generation 9) the Health, Aging and Body Composition Study (Health ABC) 10) Jackson Heart Study (JHS) 11) Multi-Ethnic Study of Atherosclerosis (MESA), 12) Multiple Risk Factor Intervention Trial (MRFIT) 13) the Reasons for Geographic and Racial Differences in Stroke Study (REGARDS) 14) Rancho Bernardo Study (RBS) 15) the Strong Heart Study (SHS)
MI: myocardial infarction; CHD: coronary heart disease; CVD: cardiovascular disease

© 2025 Tasdighi E et al. *JAMA Network Open*.

Table e6 supplementary. The total number of individuals and events included in each analysis				
	**Cigarette**	**Cigar**	**Pipe**	**Smokeless**
**MI, No.**				
Number of events	**9,103**	**4,469**	**3,935**	**5,231**
Total observation	97,417	45,901	41,845	63,395
**Stroke, No.**				
Number of events	**6,090**	**3,085**	**2,828**	**4,145**
Total observation	98,234	46,142	42,077	63,954
**CHD, No.**				
Number of events	**11,969**	**5,599**	**4,799**	**6,800**
Total observation	97,985	45,949	41,867	63,411
**CVD, No.**				
Number of events	**19,448**	**9,092**	**8,098**	**12,403**
Total observation	98,752	46,301	42,210	64,131
**Heart failure, No.**				
Number of events	**10,447**	**6,530**	**5,616**	**7,516**
Total observation	83,597	39,780	40,530	63,179
**Atrial fibrillation, No.**				
Number of events	**8,237**	**6,070**	**5,345**	**5,562**
Total observation	57,617	39,780	35,782	41,091
**CHD Mortality, No.**				
Number of events	**5,149**	**2,161**	**1,790**	**2,680**
Total observation	97,249	45,242	41,142	63,756
**CVD Mortality, No.**				
Number of events	**9,819**	**4,034**	**3,599**	**6,007**
Total observation	92,037	40,194	36,166	58,691
**All-cause mortality, No.**				
Number of events	**33,549**	**15,799**	**14,146**	**21,608**
Total observation	98,790	46,725	42,624	63,977
MI: myocardial infarction; CHD: coronary heart disease; CVD: cardiovascular disease				

© 2025 Tasdighi E et al. *JAMA Network Open*.

Table e7 Supplementary. Association between current use of traditional and non-cigarette tobacco products and health outcomes compared with non-use of the given tobacco product accounting for the heterogeneity between cohorts.

	Cigarette	Cigar	Pipe	Smokeless
**MI**				
Model 1	**1.65 (1.56-1.74)**	1.05 (0.89-1.24)	1.05 (0.85-1.29)	**1.30 (1.12-1.51)**
Model 2	**1.79 (1.69-1.89)**	1.12 (0.94-1.32)	1.21 (0.98-1.49)	**1.20 (1.03-1.39)**
**Stroke**				
Model 1	**1.48 (1.39-1.58)**	**1.24 (1.01-1.53)**	1.05 (0.79-1.40)	**1.14 (0.95-1.38)**
Model 2	**1.61 (1.51-1.73)**	**1.25 (1.01-1.55)**	1.09 (0.82-1.46)	1.09 (0.90-1.32)
**CHD**				
Model 1	**1.62 (1.54-1.69)**	1.05 (0.90-1.21)	0.95 (0.79-1.16)	**1.30 (1.14-1.48)**
Model 2	**1.76 (1.68-1.85)**	**1.12 (0.96-1.30)**	1.09 (0.90-1.33)	**1.19 (1.04-1.36)**
**CVD**				
Model 1	**1.63 (1.57-1.69)**	1.11 (0.98-1.24)	1.01 (0.86-1.18)	**1.30 (1.17-1.43)**
Model 2	**1.80 (1.73-1.87)**	**1.15 (1.02-1.30)**	1.10 (0.94-1.29)	**1.19 (1.08-1.32)**
**Heart failure**				
Model 1	**1.67 (1.59-1.76)**	**1.23 (1.06-1.43)**	1.08 (0.89-1.30)	**1.33 (1.18-1.50)**
Model 2	**1.99 (1.89-2.10)**	**1.29 (1.10-1.51)**	**1.23 (1.01-1.49)**	**1.20 (1.06-1.36)**
**Atrial fibrillation**				
Model 1	**1.44 (1.35-1.52)**	**1.31 (1.13-1.51)**	0.97 (0.81-1.18)	**1.21 (1.03-1.41)**
Model 2	**1.61 (1.52-1.71)**	**1.32 (1.13-1.53)**	1.00 (0.82-1.21)	1.13 (0.96-1.33)
**CHD Mortality**				
Model 1	**1.69 (1.58-1.82)**	1.09 (0.88-1.36)	0.83 (0.60-1.16)	**1.42 (1.17-1.72)**
Model 2	**1.91 (1.77-2.06)**	1.20 (0.96-1.50)	1.00 (0.72-1.39)	**1.31 (1.08-1.59)**
**CVD Mortality**				
Model 1	**1.67 (1.59-1.76)**	1.14 (0.96-1.35)	0.91 (0.72-1.15)	**1.36 (1.19-1.56)**
Model 2	**1.87 (1.77-1.98)**	1.18 (0.99-1.40)	1.00 (0.79-1.28)	1.23 (1.07-1.41)
**All-cause mortality**				
Model 1	**1.99 (1.93-2.04)**	**1.12 (1.02-1.22)**	1.05 (0.93-1.18)	**1.26 (1.17-1.36)**
Model 2	**2.13 (2.07-2.20)**	**1.13 (1.03-1.24)**	1.08 (0.96-1.22)	**1.21 (1.11-1.30)**

*Reference group consists of individuals who have never smoked the specific tobacco product under consideration.
The heterogeneity between cohorts was accounted for by including the frailty term in survival analysis models.
Model 1 adjusted for age, sex, race and ethnicity, former/cigarette smoking status, education status, and history of coronary heart disease cohort.
Model 2 adjusted for age, sex, race and ethnicity, former/cigarette smoking status, education status, history of coronary heart disease, cohort, body mass index, hypertension, diabetes, antihypertensive and lipid-lowering
*Both model 1 and model 2 were not adjusted for former/cigarette smoking status for cigarette tobacco analysis
ψ P-Value=0.063
MI: myocardial infarction; CHD: coronary heart disease; CVD: cardiovascular disease

© 2025 Tasdighi E et al. *JAMA Network Open*.

Table e8 supplementary. Association between Sole and Exclusive use of non-cigarette tobacco products and health outcomes compared with non-use of the given tobacco product accounting for the heterogeneity between cohorts.

	Cigar use status		Pipe use status		Smokeless use status	
	Sole[1]	Exclusive[2]	Sole	Exclusive	Sole	Exclusive
**MI**						
Model 1	1.12 (0.97-1.29)	1.05 (0.86-1.29)	**1.33 (1.09-1.61)**	**1.34 (1.03-1.74)**	**1.58 (1.33-1.87)**	**1.34(1.03-1.74)**
Model 2	1.15 (0.99-1.34)	1.20 (0.91-1.56)	**1.45 (1.19-1.76)**	**1.32 (1.01-1.74)**	**1.43 (1.20-1.70)**	1.24 (0.94-1.63)
**Stroke**						
Model 1	**1.33 (1.10-1.60)**	**1.51 (1.19-1.92)**	1.16 (0.85-1.57)	1.11 (0.75-1.66)	1.20 (0.95-1.45)	1.19 (0.89-1.61)
Model 2	**1.35 (1.13-1.62)**	**1.52 (1.21-1.98)**	1.21 (0.91-1.63)	1.12 (0.74-1.69)	1.14 (0.92-1.43)	1.12 (0.82-1.53)
**CHD**						
Model 1	1.12 (0.99-1.28)	1.07 (0.90-1.28)	1.17 (0.98-1.40)	**1.44 (1.15-1.79)**	**1.57 (1.36-1.82)**	**1.44(1.15-1.79)**
Model 2	**1.17 (1.03-1.34)**	1.16 (0.97-1.39)	**1.27 (1.06-1.53)**	1.12 (0.86-1.45)	**1.42 (1.22-1.64)**	**1.36 (1.09-1.71)**
**CVD**						
Model 1	**1.16 (1.05-1.28)**	**1.21 (1.05-1.39)**	**1.16 (1.00-1.35)**	**1.44 (1.22-1.69)**	**1.47 (1.31-1.64)**	**1.44(1.22-1.69)**
Model 2	**1.19 (1.07-1.32)**	**1.27 (1.11-1.47)**	**1.23 (1.06-1.44)**	1.13 (0.91-1.40)	**1.34 (1.19-1.50)**	**1.34 (1.14-1.59)**
**Heart failure**						
Model 1	**1.32 (1.13-1.54)**	**1.35 (1.09-1.66)**	**1.28 (1.03-1.60)**	**1.78 (1.47-2.14)**	**1.56 (1.35-1.80)**	**1.78(1.47-2.14)**
Model 2	**1.33 (1.14-1.56)**	**1.37 (1.10-1.70)**	**1.42 (1.13-1.78)**	0.96 (0.67-1.38)	**1.42 (1.22-1.64)**	**1.70 (1.40-2.06)**
**Atrial fibrillation**						
Model 1	**1.31 (1.12-1.54)**	**1.39 (1.14-1.71)**	1.20 (0.97-1.48)	1.23 (0.91-1.66)	**1.45 (1.20-1.75)**	1.23(0.91-1.66)
Model 2	**1.27 (1.08-1.49)**	**1.34 (1.08-1.65)**	1.23 (0.99-1.52)	1.03 (0.75-1.41)	**1.31 (1.08-1.58)**	1.06 (0.78-1.45)
**CHD Mortality**						
Model 1	**1.23 (1.02-1.49)**	1.11 (0.85-1.45)	1.10 (0.83-1.45)	**1.66 (1.22-2.26)**	**1.67 (1.34-2.07)**	**1.66(1.22-2.26)**
Model 2	**1.29 (1.06-1.56)**	1.20 (0.91-1.57)	1.17 (0.88-1.54)	0.75 (0.48-1.18)	**1.55 (1.24-1.94)**	**1.67 (1.22-2.28)**
**CVD Mortality**						
Model 1	**1.22 (1.06-1.41)**	**1.30 (1.08-1.57)**	1.06 (0.85-1.32)	**1.65 (1.34-2.03)**	**1.55 (1.33-1.80)**	**1.65(1.34-2.03)**
Model 2	**1.25 (1.08-1.45)**	**1.37 (1.13-1.66)**	1.10 (0.88-1.37)	0.99 (0.73-1.35)	**1.41 (1.20-1.65)**	**1.55 (1.25-1.92)**
**All-cause mortality**						
Model 1	**1.27 (1.17-1.38)**	**1.19 (1.07-1.34)**	**1.27 (1.13-1.44)**	1.43 (1.27-1.62)	**1.52 (1.39-1.66)**	**1.43(1.27-1.62)**
Model 2	**1.28 (1.18-1.39)**	**1.21 (1.08-1.36)**	**1.29 (1.14-1.46)**	1.13 (0.95-1.35)	**1.46 (1.34-1.60)**	**1.39 (1.22-1.58)**

The reference group includes only participants with never cigarette use and no reported non-cigarette products for both sole and exclusive analysis.
The heterogeneity between cohorts was accounted for by including the frailty term in survival analysis models.
1 Sole use is defined as current non-cigarette tobacco use without current cigarette use.
2 Exclusive use is defined as current non-cigarette tobacco without any history of cigarette use.
Model 1 adjusted for age, sex, race and ethnicity, former/cigarette smoking status, education status, and history of coronary heart disease cohort.
Model 2 adjusted for age, sex, race and ethnicity, former/cigarette smoking status, education status, history of coronary heart disease, cohort, body mass index, hypertension, diabetes, antihypertensive and lipid-lowering
¶ P-Value=0.058
Ψ P-Value=0.060
MI: myocardial infarction; CHD: coronary heart disease; CVD: cardiovascular disease

© 2025 Tasdighi E et al. *JAMA Network Open*.

Table e9 Supplementary. Association between current use of traditional and non-cigarette tobacco products and health outcomes compared with non-use of the given tobacco product considering participants' age as the time scale in the survival analysis				
	**Cigarette**	**Cigar**	**Pipe**	**Smokeless**
**MI**				
Model 1	**1.65 (1.57-1.74)**	1.05 (0.89-1.24)	1.03 (0.84-1.25)	**1.32 (1.14-1.53)**
Model 2	**1.80 (1.71-1.91)**	1.10 (0.93-1.31)	1.19 (0.97-1.45)	**1.21 (1.04-1.41)**
**Stroke**				
Model 1	**1.49 (1.39-1.59)**	**1.25 (1.01-1.54)**	1.05 (0.79-1.40)	1.17 (0.97-1.41)
Model 2	**1.63 (1.52-1.74)**	**1.27 (1.02-1.57)**	1.10 (0.82-1.47)	1.11 (0.91-1.35)
**CHD**				
Model 1	**1.63 (1.56-1.71)**	1.05 (0.91-1.21)	0.94 (0.78-1.14)	**1.32 (1.16-1.51)**
Model 2	**1.79 (1.71-1.88)**	1.12 (0.96-1.29)	1.08 (0.89-1.32)	**1.21 (1.06-1.38)**
**CVD**				
Model 1	**1.66 (1.60-1.72)**	1.12 (0.99-1.25)	1.00 (0.86-1.17)	**1.33 (1.20-1.46)**
Model 2	**1.84 (1.77-1.91)**	**1.17 (1.04-1.31)**	1.11 (0.95-1.30)	**1.22 (1.10-1.35)**
**Heart failure**				
Model 1	**1.68 (1.60-1.77)**	**1.25 (1.07-1.45)**	1.09 (0.90-1.31)	**1.35 (1.20-1.52)**
Model 2	**2.02 (1.92-2.14)**	**1.31 (1.12-1.52)**	**1.24 (1.02-1.51)**	**1.22 (1.07-1.38)**
**Atrial fibrillation**				
Model 1	**1.44 (1.36-1.53)**	**1.31 (1.13-1.52)**	0.97 (0.80-1.17)	1.23 (1.05-1.44)
Model 2	**1.63 (1.53-1.73)**	**1.32 (1.13-1.53)**	1.00 (0.82-1.21)	1.14 (0.97-1.35)
**CHD Mortality**				
Model 1	**1.71 (1.59-1.83)**	1.10 (0.89-1.38)	0.84 (0.61-1.16)	**1.45 (1.20-1.75)**
Model 2	**1.94 (1.80-2.09)**	1.21 (0.97-1.51)	1.00 (0.72-1.40)	**1.33 (1.09-1.62)**
**CVD Mortality**				
Model 1	**1.70 (1.61-1.79)**	1.15 (0.97-1.37)	0.92 (0.73-1.16)	**1.39 (1.22-1.59)**
Model 2	**1.92 (1.82-2.03)**	**1.19 (1.00-1.42)**	1.02 (0.80-1.29)	**1.25 (1.09-1.44)**
**All-cause mortality**				
Model 1	**2.02 (1.96-2.08)**	**1.13 (1.03-1.24)**	1.06 (0.94-1.19)	**1.28 (1.19-1.38)**
Model 2	**2.18 (2.12-2.25)**	**1.15 (1.05-1.26)**	1.09 (0.97-1.24)	**1.23 (1.13-1.32)**

*Reference group consists of individuals who have never smoked the specific tobacco product under consideration.
Model 1 adjusted for age, sex, race and ethnicity, former/cigarette smoking status, education status, and history of coronary heart disease cohort.
Model 2 adjusted for age, sex, race and ethnicity, former/cigarette smoking status, education status, history of coronary heart disease, cohort, body mass index, hypertension, diabetes, antihypertensive and lipid-lowering
*Both model 1 and model 2 were not adjusted for former/cigarette smoking status for cigarette tobacco analysis
ψ P-Value=0.063
MI: myocardial infarction; CHD: coronary heart disease; CVD: cardiovascular disease

© 2025 Tasdighi E et al. *JAMA Network Open*.

Table e10 supplementary. Association between Sole and Exclusive use of non-cigarette tobacco products and health outcomes compared with non-use of the given tobacco product considering participants' age as the time scale in the survival analysis

	Cigar use status		Pipe use status		Smokeless use status	
	**Sole[1]**	**Exclusive[2]**	**Sole**	**Exclusive**	**Sole**	**Exclusive**
**MI**						
Model 1	**1.16 (1.00-1.34)**	1.11 (0.91-1.36)	**1.35 (1.12-1.64)**	1.19 (0.91-1.55)	**1.61 (1.36-1.91)**	1.39 (1.07-1.81)
Model 2	**1.19 (1.03-1.38)**	1.19 (0.97-1.46)	**1.48 (1.22-1.80)**	**1.32 (1.01-1.73)**	**1.45 (1.22-1.73)**	1.27 (0.97-1.68)
**Stroke**						
Model 1	**1.36 (1.13-1.63)**	**1.56 (1.23-1.98)**	1.18 (0.88-1.58)	1.11 (0.75-1.66)	1.22 (0.99-1.50)	1.24 (0.92-1.67)
Model 2	**1.37 (1.14-1.65)**	**1.57 (1.23-2.01)**	1.21 (0.89-1.63)	1.11 (0.73-1.69)	1.15 (0.93-1.44)	1.15 (0.84-1.58)
**CHD**						
Model 1	**1.17 (1.03-1.33)**	1.13 (0.95-1.35)	**1.20 (1.01-1.44)**	1.03 (0.80-1.33)	**1.61 (1.39-1.86)**	**1.50 (1.20-1.87)**
Model 2	**1.22 (1.07-1.38)**	**1.22 (1.02-1.45)**	**1.31 (1.09-1.58)**	1.14 (0.88-1.48)	**1.44 (1.24-1.67)**	**1.40 (1.12-1.75)**
**CVD**						
Model 1	**1.20 (1.09-1.33)**	**1.27 (1.10-1.46)**	**1.19 (1.02-1.39)**	1.08 (0.88-1.34)	**1.51 (1.35-1.69)**	**1.50 (1.28-1.76)**
Model 2	**1.23 (1.11-1.37)**	**1.32 (1.15-1.52)**	**1.27 (1.09-1.48)**	1.15 (0.93-1.42)	**1.37 (1.22-1.54)**	**1.39 (1.18-1.65)**
**Heart failure**						
Model 1	**1.35 (1.16-1.58)**	**1.40 (1.13-1.72)**	**1.27 (1.02-1.58)**	0.88 (0.62-1.25)	**1.59 (1.38-1.84)**	**1.84 (1.52-2.21)**
Model 2	**1.36 (1.17-1.59)**	**1.41 (1.13-1.74)**	**1.41 (1.12-1.77)**	0.95 (0.66-1.36)	**1.44 (1.24-1.67)**	**1.74 (1.43-2.11)**
**Atrial fibrillation**						
Model 1	**1.33 (1.14-1.56)**	**1.42 (1.16-1.74)**	1.19 (0.97-1.47)	1.01 (0.74-1.37)	**1.47 (1.22-1.77)**	1.27 (0.94-1.71)
Model 2	**1.28 (1.09-1.51)**	**1.36 (1.10-1.67)**	1.23 (0.99-1.53)	1.02 (0.74-1.40)	**1.32 (1.09-1.60)**	1.09 (0.80-1.49)
**CHD Mortality**						
Model 1	**1.28 (1.06-1.55)**	1.16 (0.89-1.51)	1.10 (0.84-1.46)	0.68 (0.44-1.08)	**1.71 (1.38-2.12)**	**1.68 (1.24-2.29)**
Model 2	**1.33 (1.10-1.61)**	1.23 (0.94-1.62)	1.17 (0.89-1.56)	0.75 (0.48-1.18)	**1.58 (1.27-1.97)**	**1.67 (1.22-2.28)**
**CVD Mortality**						
Model 1	**1.27 (1.10-1.46)**	**1.35 (1.12-1.62)**	1.06 (0.85-1.32)	0.94 (0.69-1.28)	**1.59 (1.37-1.85)**	**1.67 (1.36-2.05)**
Model 2	**1.29 (1.11-1.49)**	**1.39 (1.15-1.69)**	1.10 (0.88-1.37)	0.99 (0.73-1.35)	**1.45 (1.24-1.70)**	**1.56 (1.26-1.94)**
**All-cause mortality**						
Model 1	**1.32 (1.22-1.43)**	**1.24 (1.11-1.39)**	**1.29 (1.14-1.46)**	1.12 (0.95-1.34)	**1.56 (1.43-1.70)**	**1.45 (1.28-1.64)**
Model 2	**1.32 (1.22-1.44)**	**1.25 (1.11-1.41)**	**1.31 (1.15-1.49)**	1.14 (0.96-1.36)	**1.50 (1.38-1.64)**	**1.41 (1.24-1.60)**

The reference group includes only participants with never cigarette use and no reported non-cigarette products for both sole and exclusive analysis.
the participants' age was considered the time variable in this analysis
1 Sole use is defined as current non-cigarette tobacco use without current cigarette use.
2 Exclusive use is defined as current non-cigarette tobacco without any history of cigarette use.
Model 1 adjusted for age, sex, race and ethnicity, former/cigarette smoking status, education status, and history of coronary heart disease cohort.
Model 2 adjusted for age, sex, race and ethnicity, former/cigarette smoking status, education status, history of coronary heart disease, cohort, body mass index, hypertension, diabetes, antihypertensive and lipid-lowering
¶ P-Value=0.058
Ψ P-Value=0.060
MI: myocardial infarction; CHD: coronary heart disease; CVD: cardiovascular disease

© 2025 Tasdighi E et al. *JAMA Network Open*.

eAppendix. Meta-analysis of each association based on individual cohorts.

Cigar status and health outcomes in each cohort.

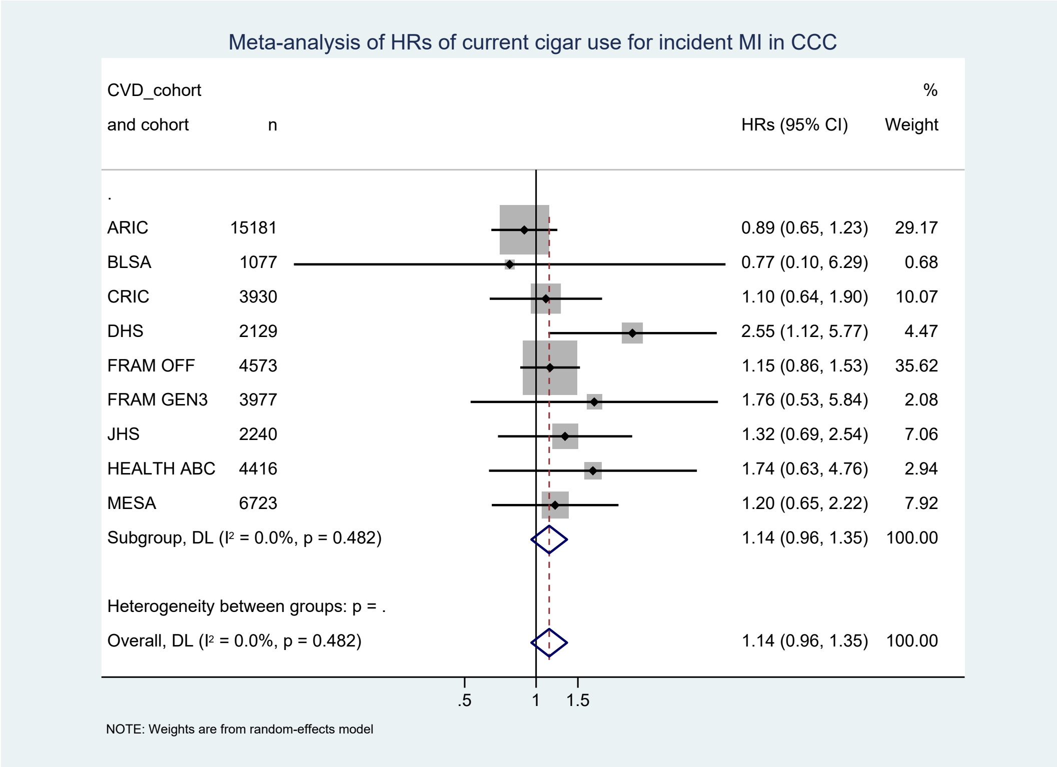

© 2025 Tasdighi E et al. *JAMA Network Open*.

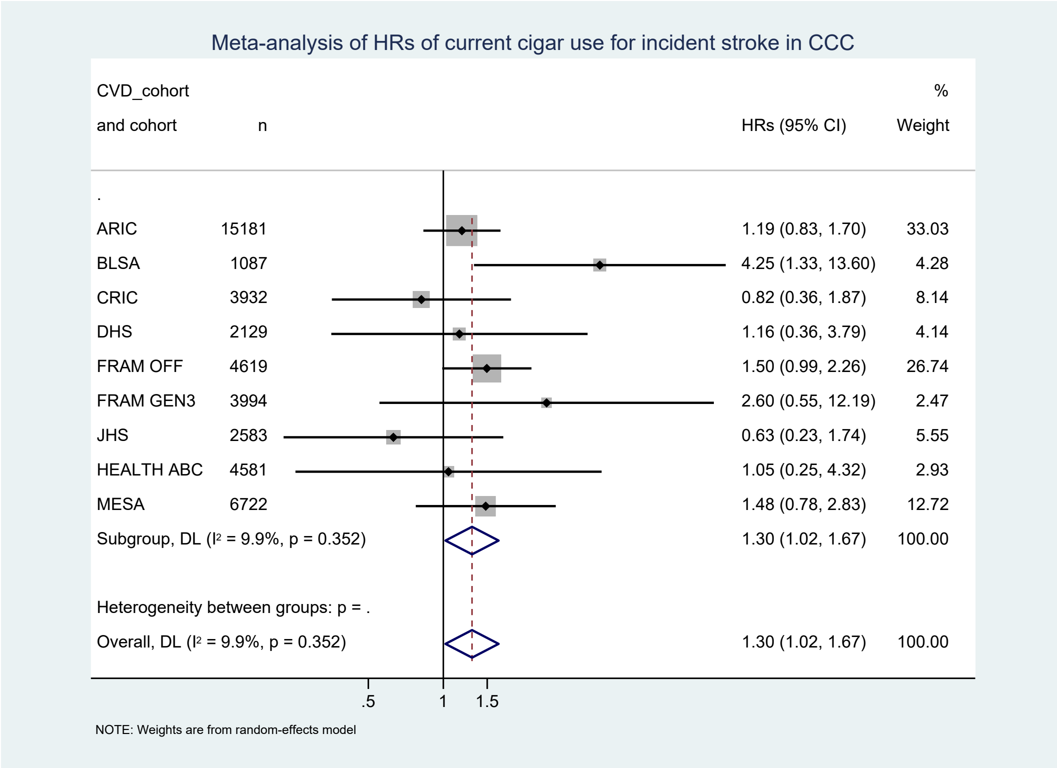

© 2025 Tasdighi E et al. *JAMA Network Open*.

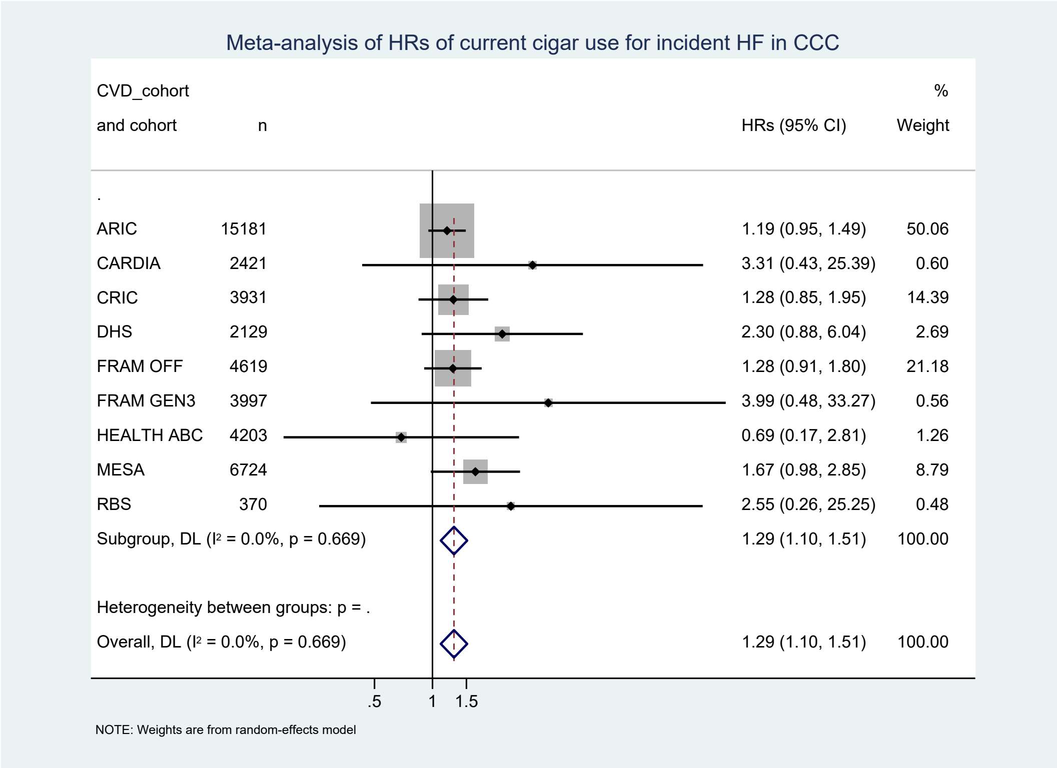

© 2025 Tasdighi E et al. *JAMA Network Open*.

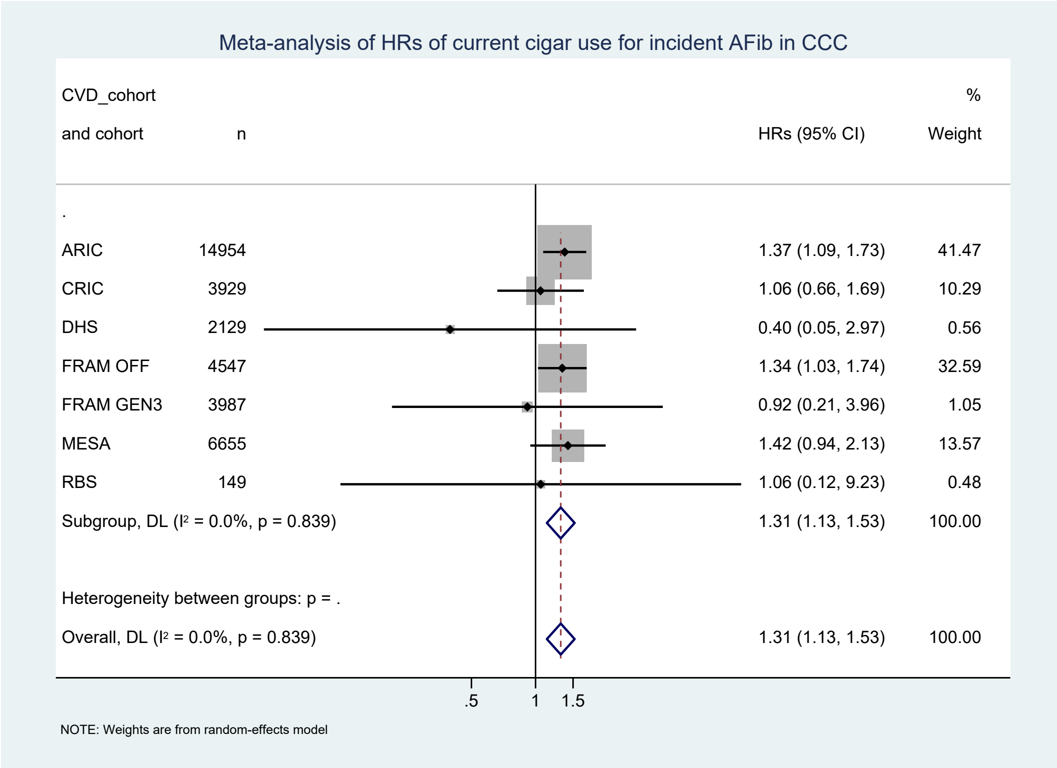

© 2025 Tasdighi E et al. *JAMA Network Open*.

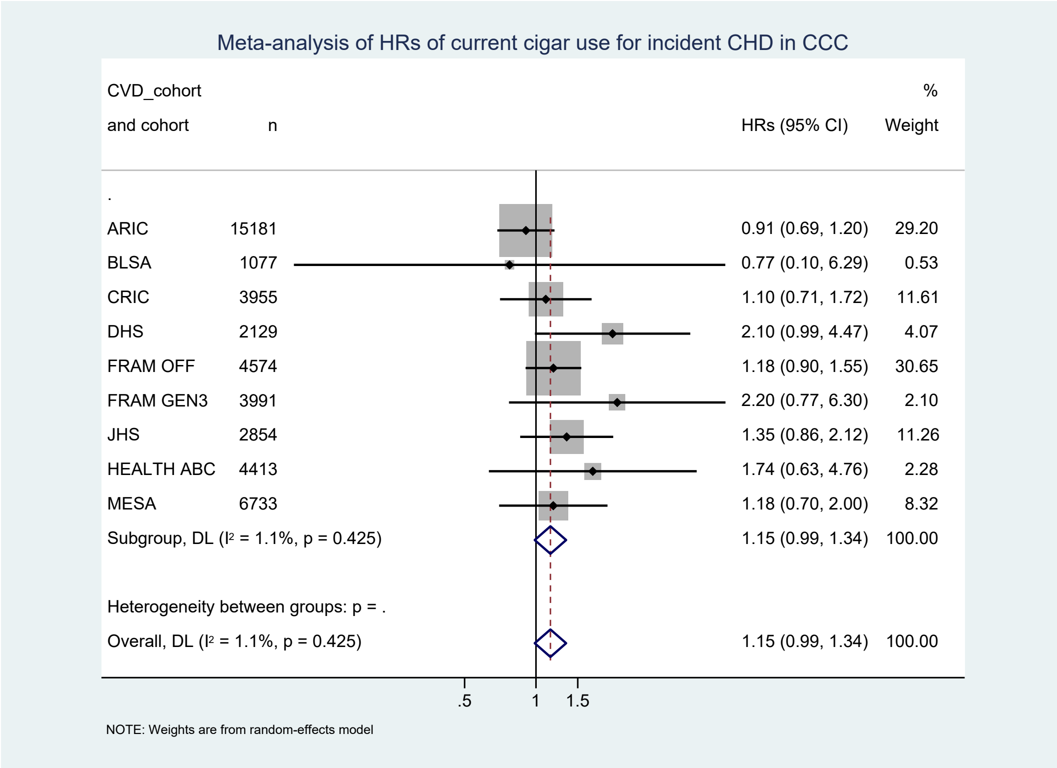

© 2025 Tasdighi E et al. *JAMA Network Open*.

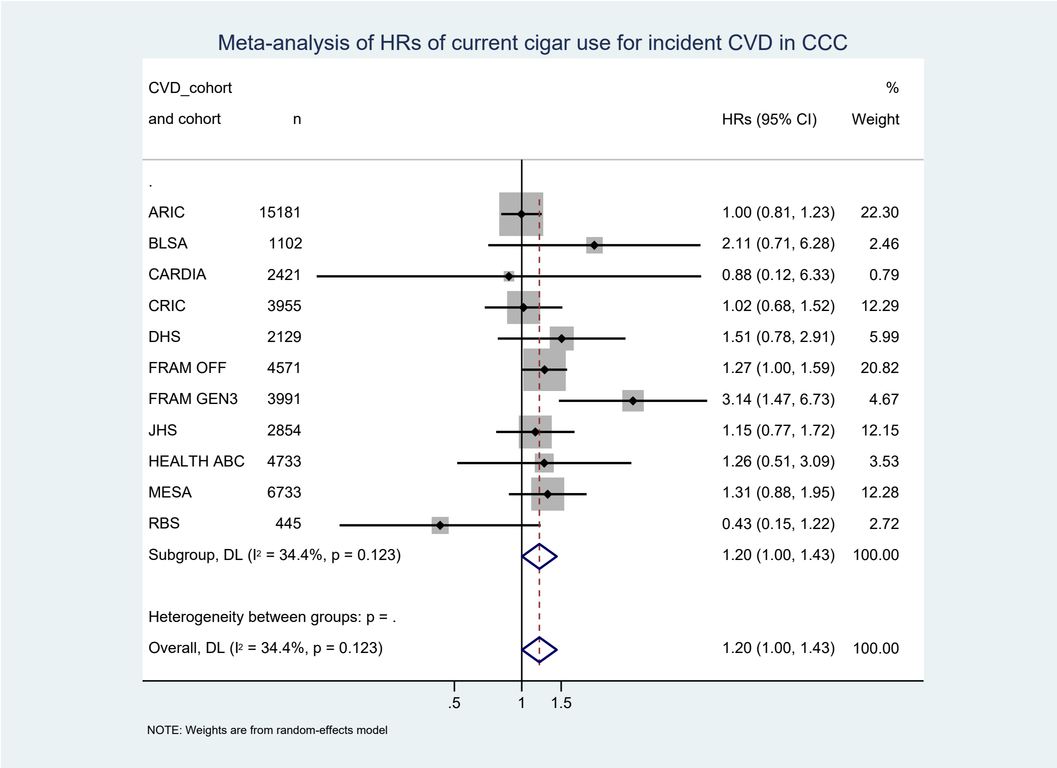

© 2025 Tasdighi E et al. *JAMA Network Open*.

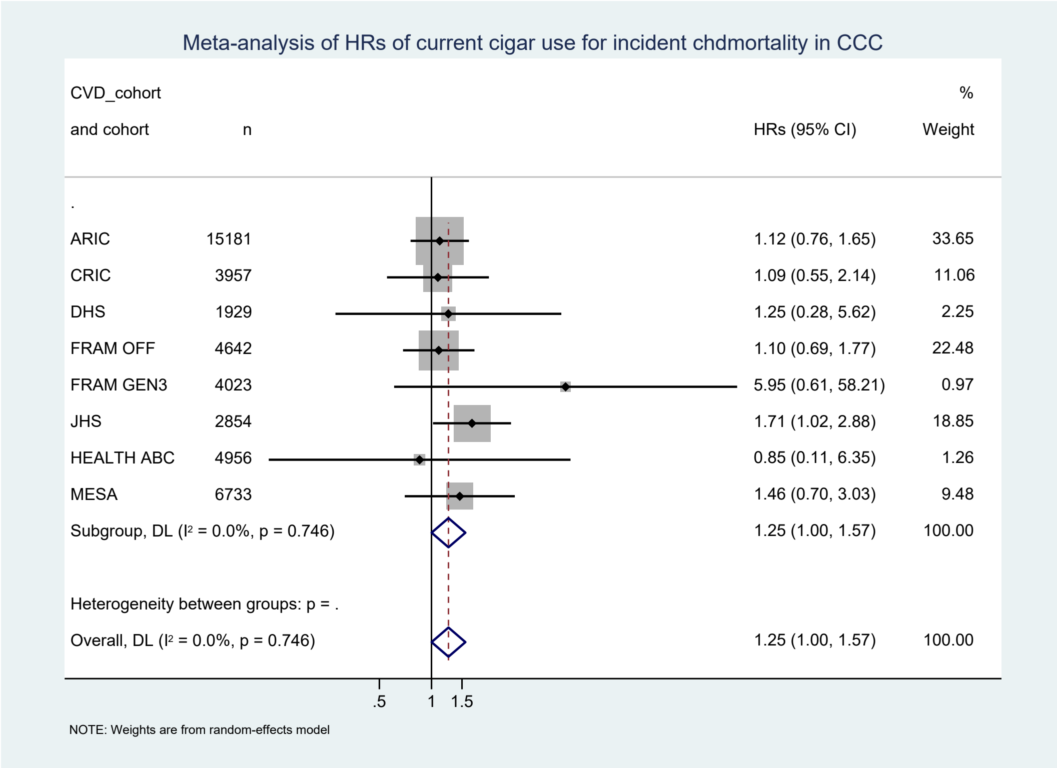

© 2025 Tasdighi E et al. *JAMA Network Open*.

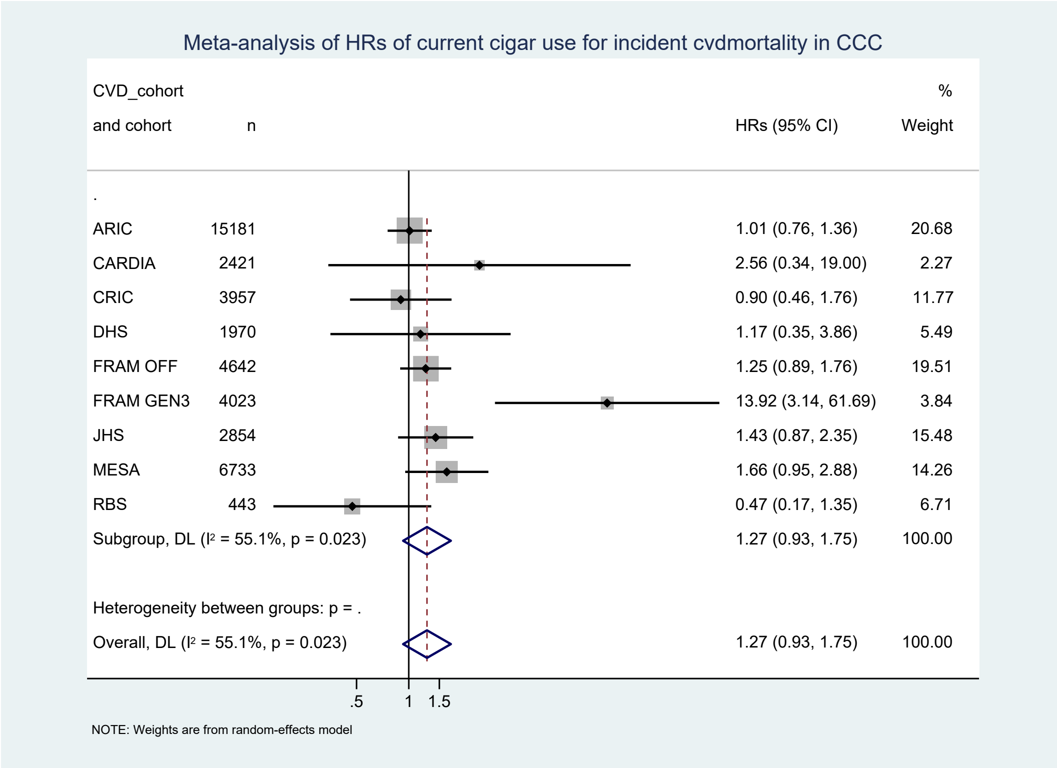

© 2025 Tasdighi E et al. *JAMA Network Open*.

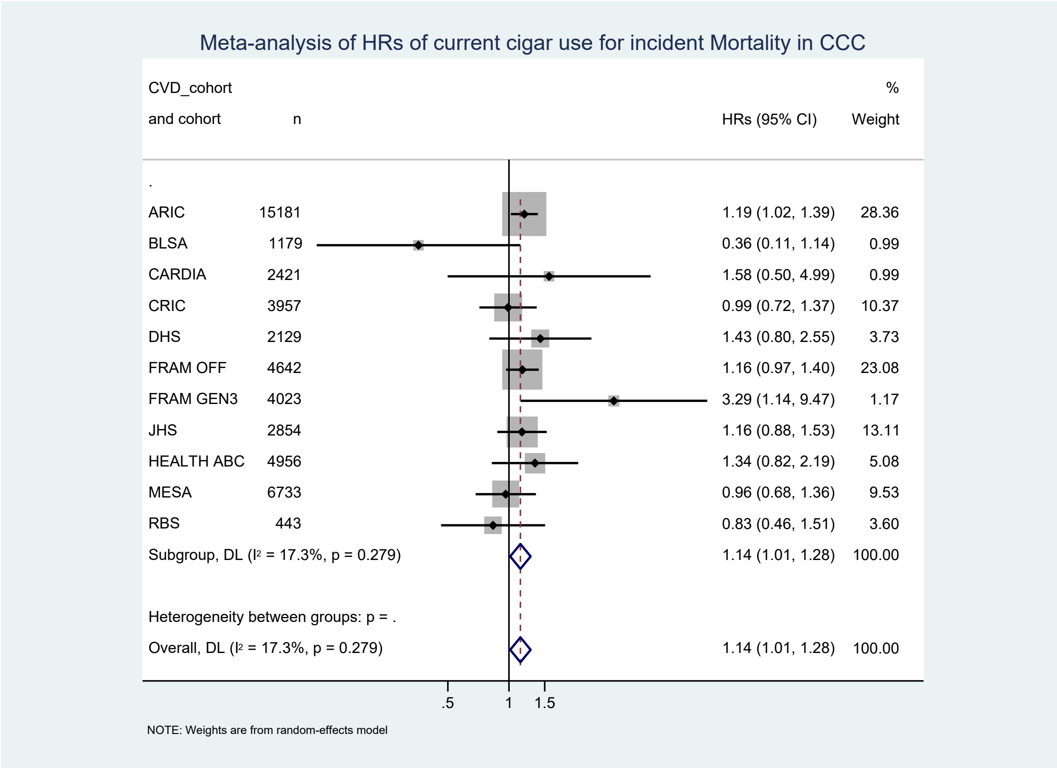

Pipe Status and health outcomes in each cohort

© 2025 Tasdighi E et al. *JAMA Network Open*.

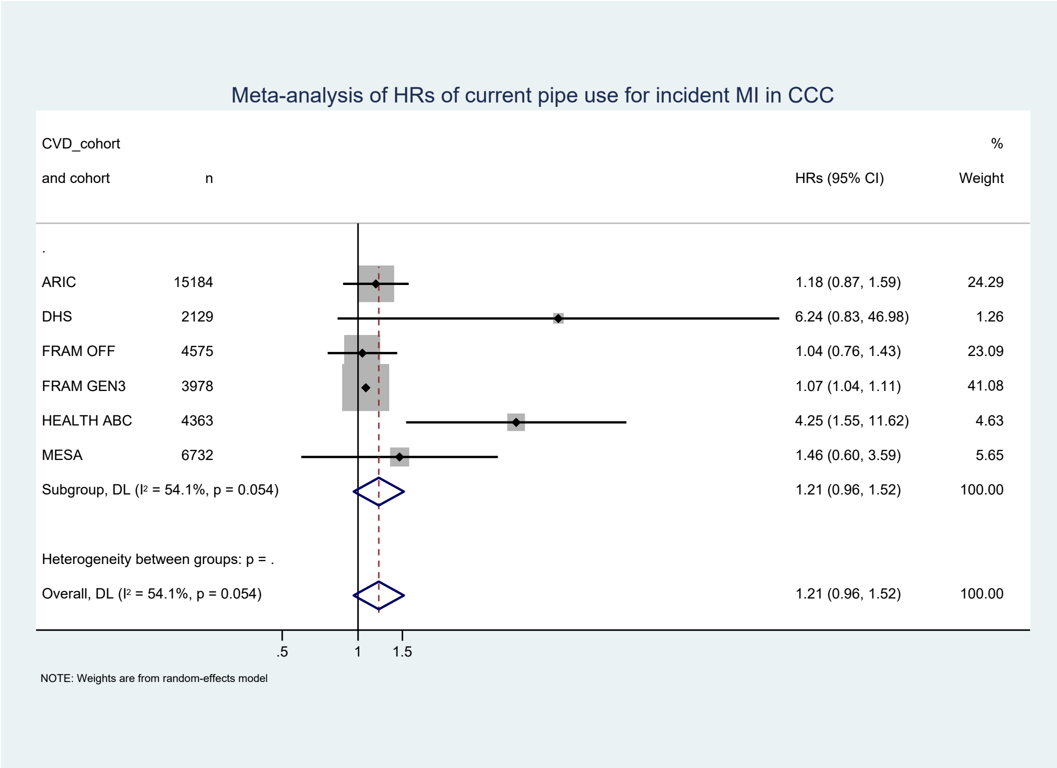

© 2025 Tasdighi E et al. *JAMA Network Open*.

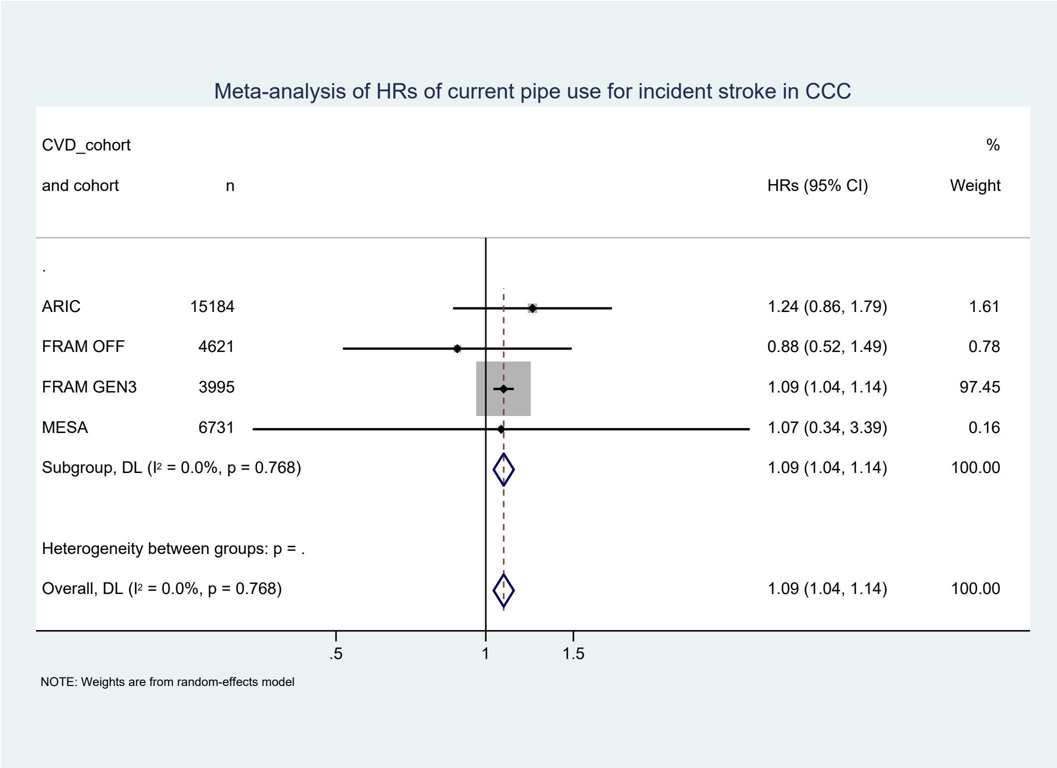

© 2025 Tasdighi E et al. *JAMA Network Open*.

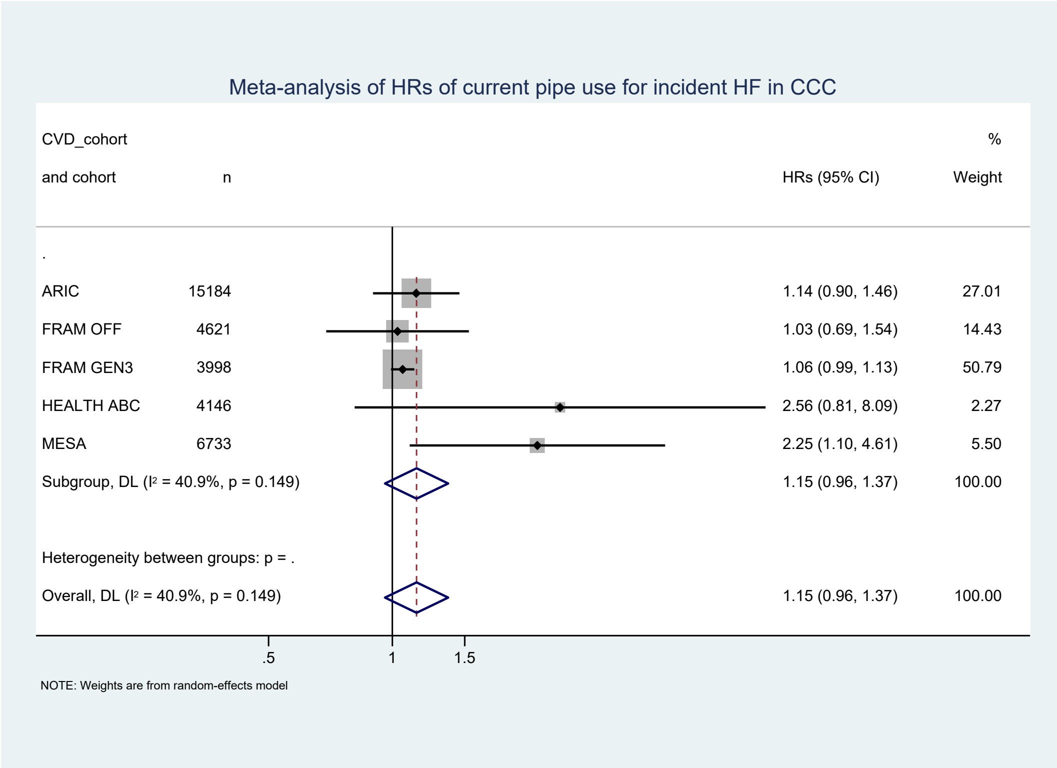

© 2025 Tasdighi E et al. *JAMA Network Open*.

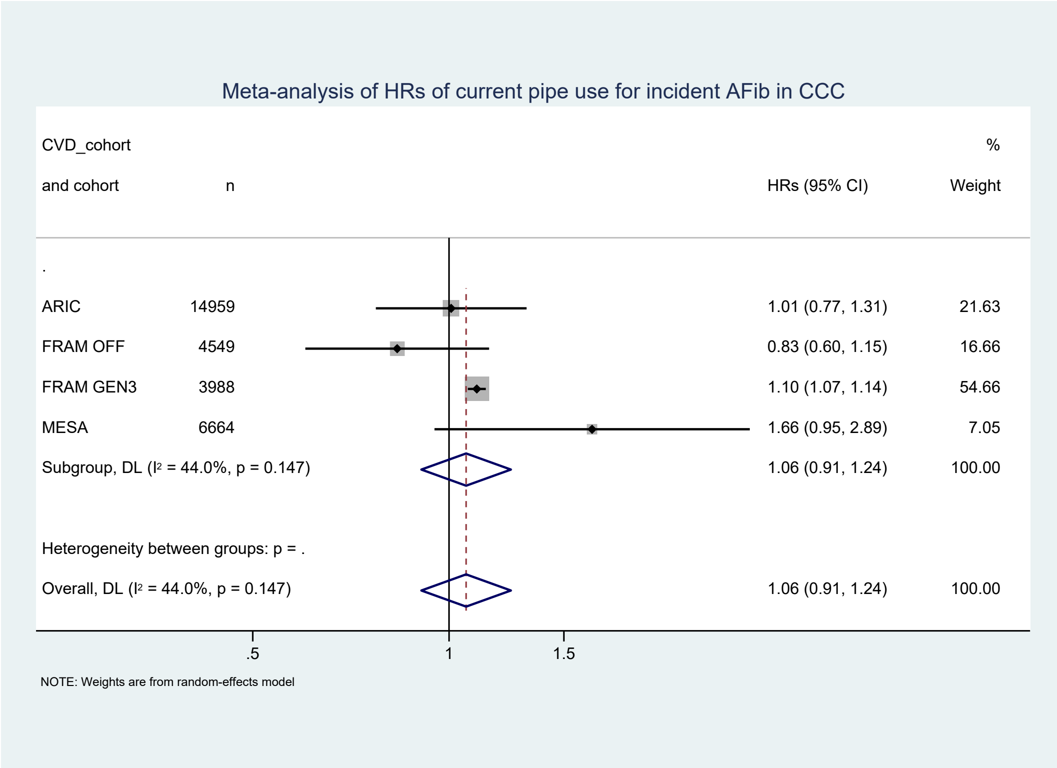

© 2025 Tasdighi E et al. *JAMA Network Open*.

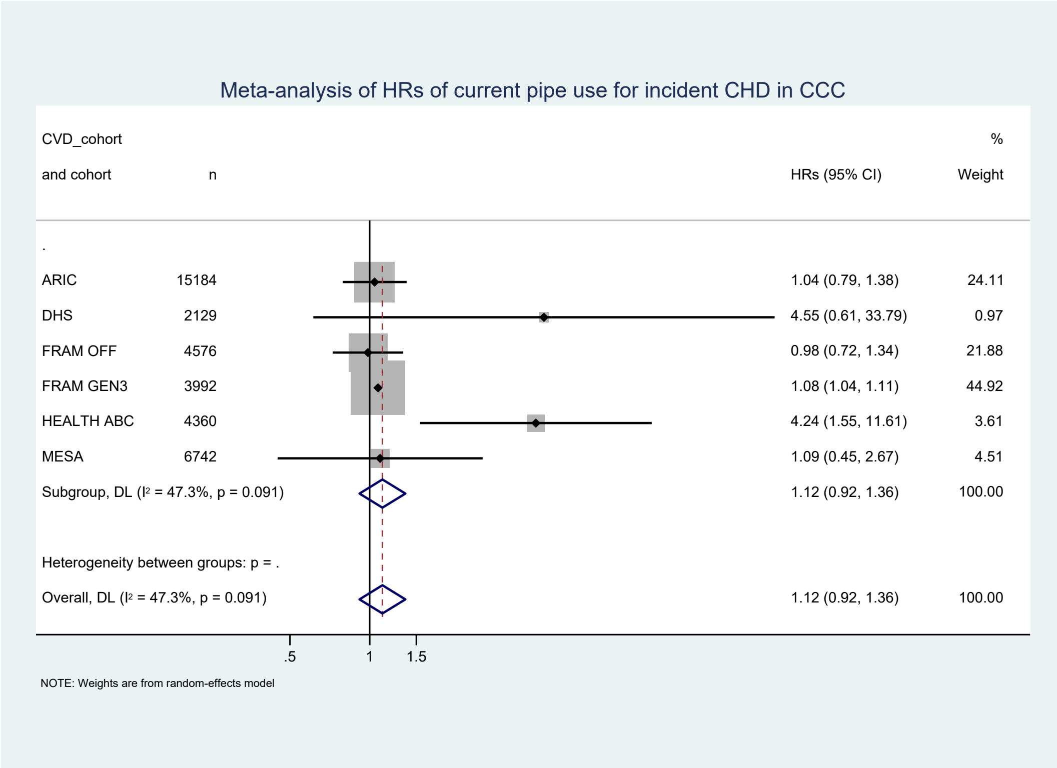

© 2025 Tasdighi E et al. *JAMA Network Open*.

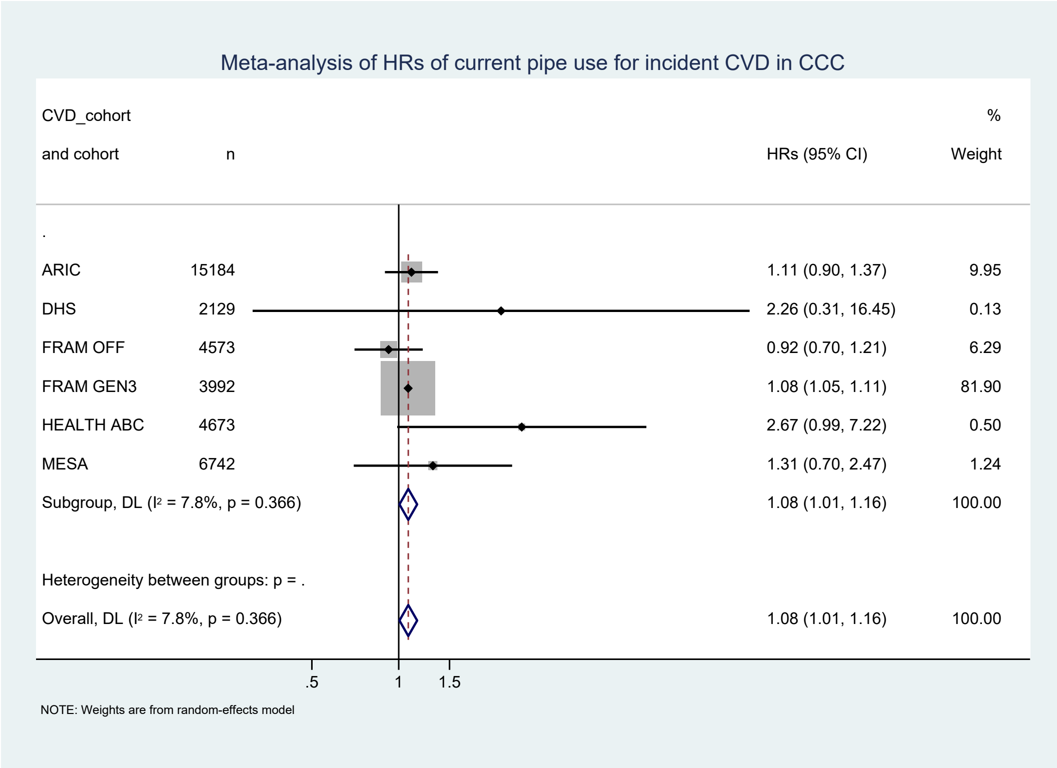

© 2025 Tasdighi E et al. *JAMA Network Open*.

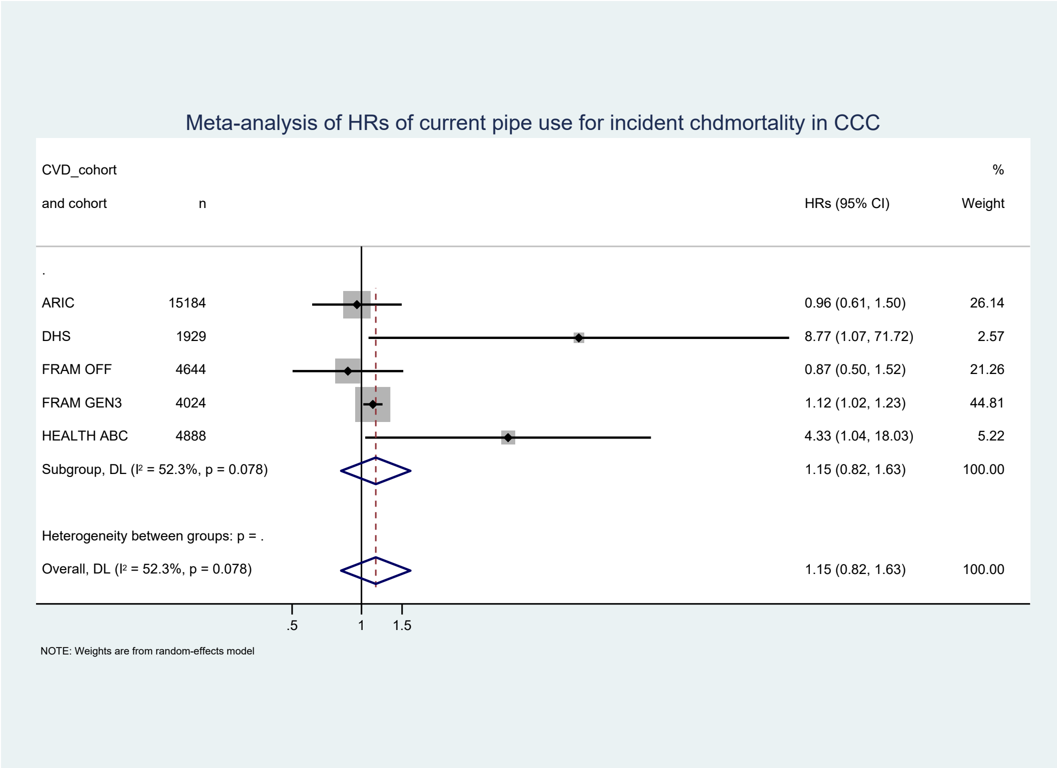

© 2025 Tasdighi E et al. *JAMA Network Open*.

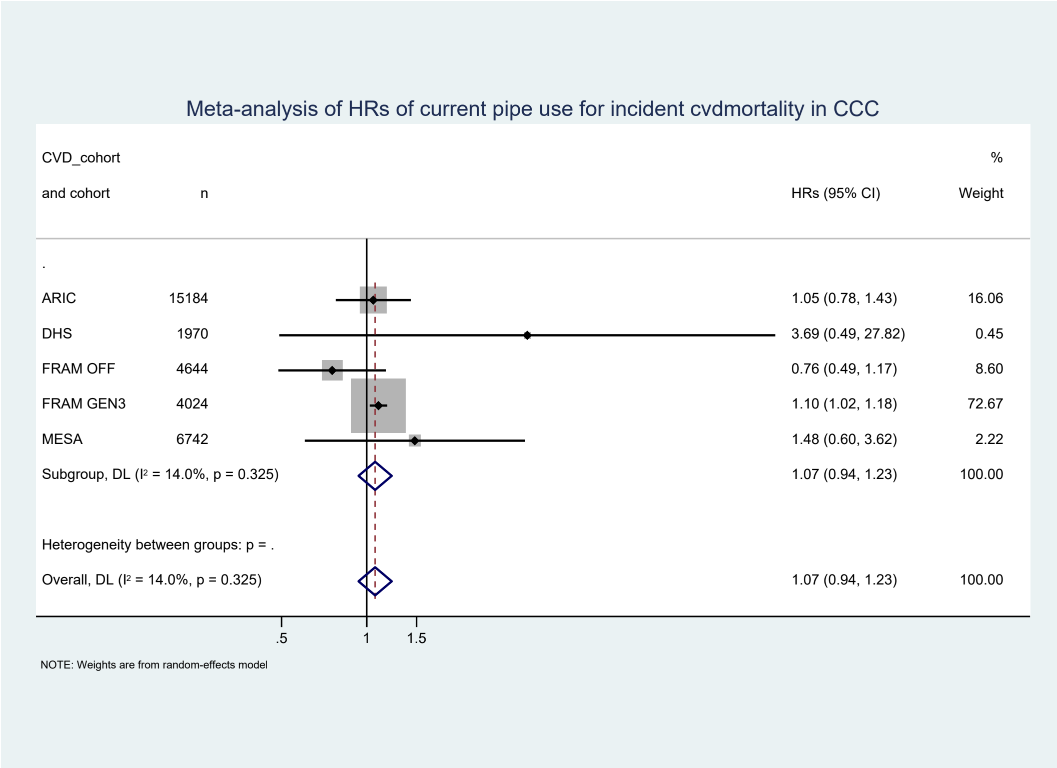

© 2025 Tasdighi E et al. *JAMA Network Open*.

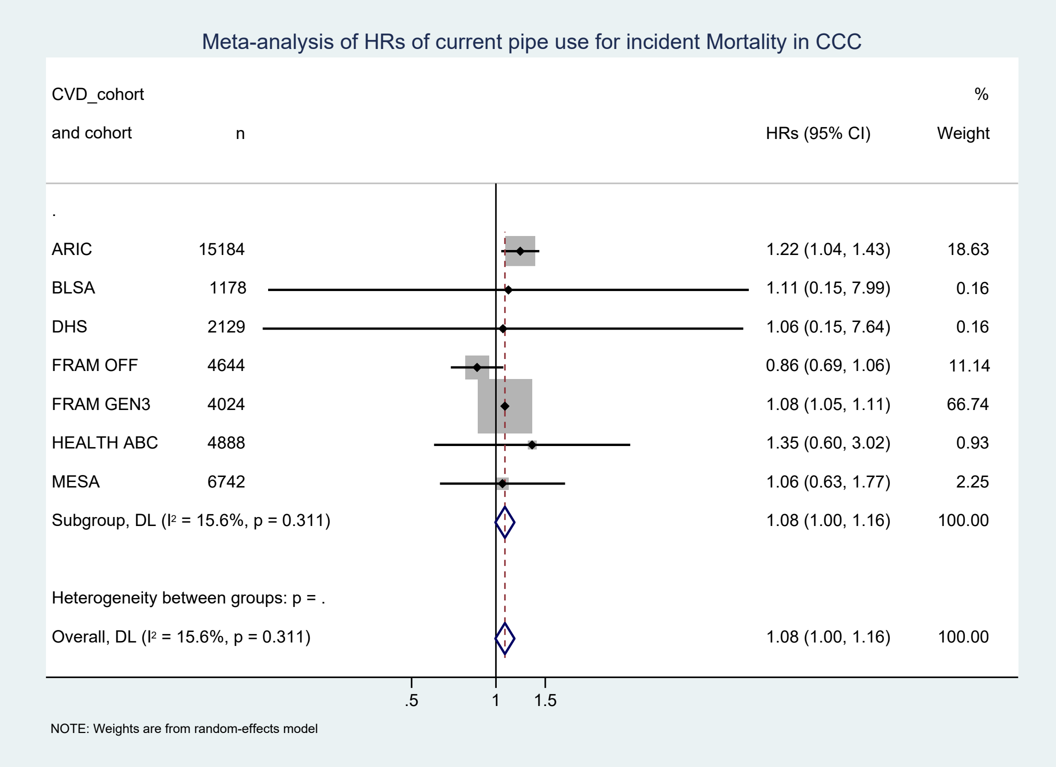

Smokeless status and health outcomes in each cohort.

© 2025 Tasdighi E et al. *JAMA Network Open*.

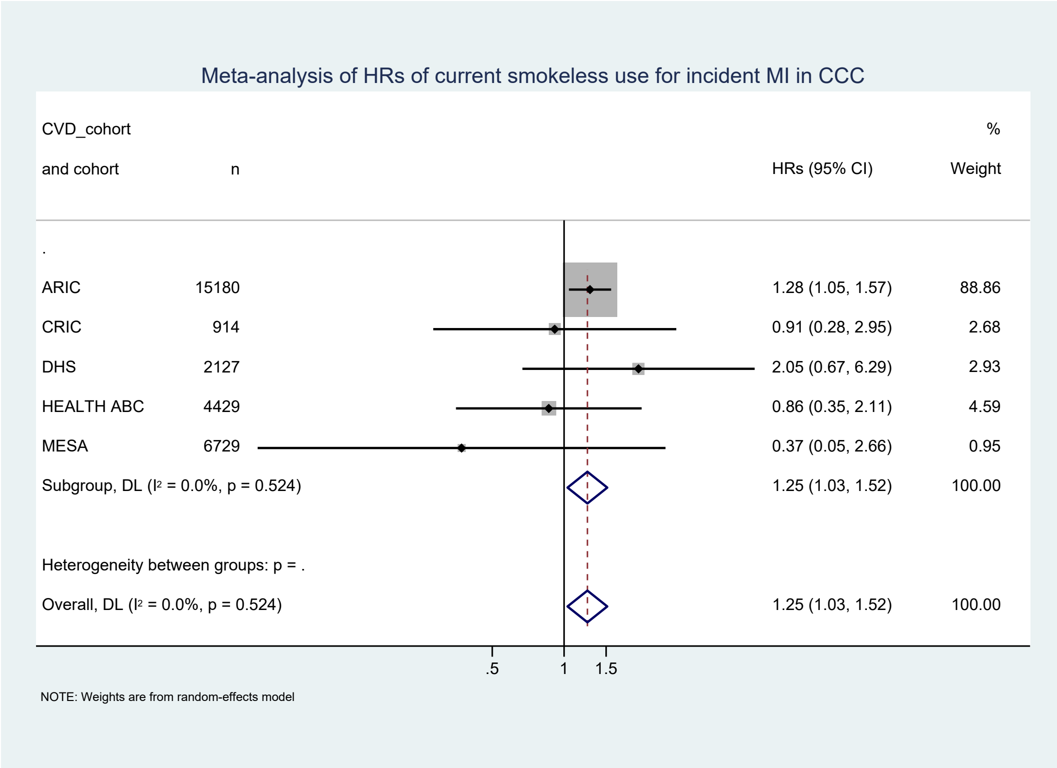

© 2025 Tasdighi E et al. *JAMA Network Open*.

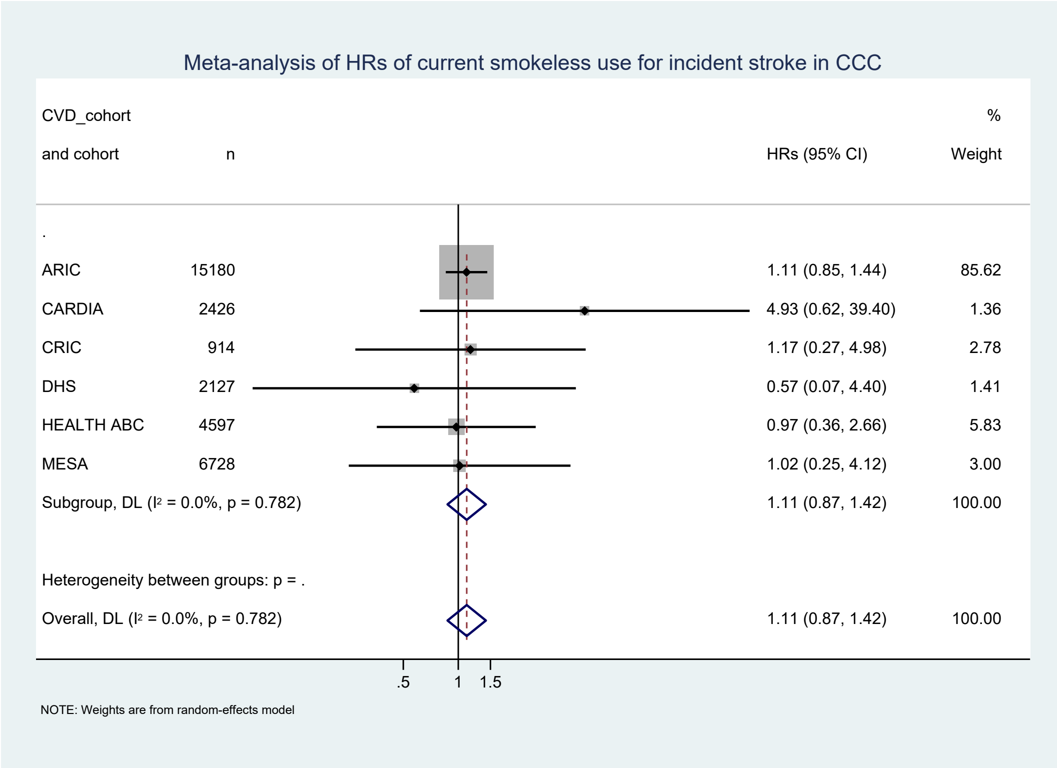

© 2025 Tasdighi E et al. *JAMA Network Open*.

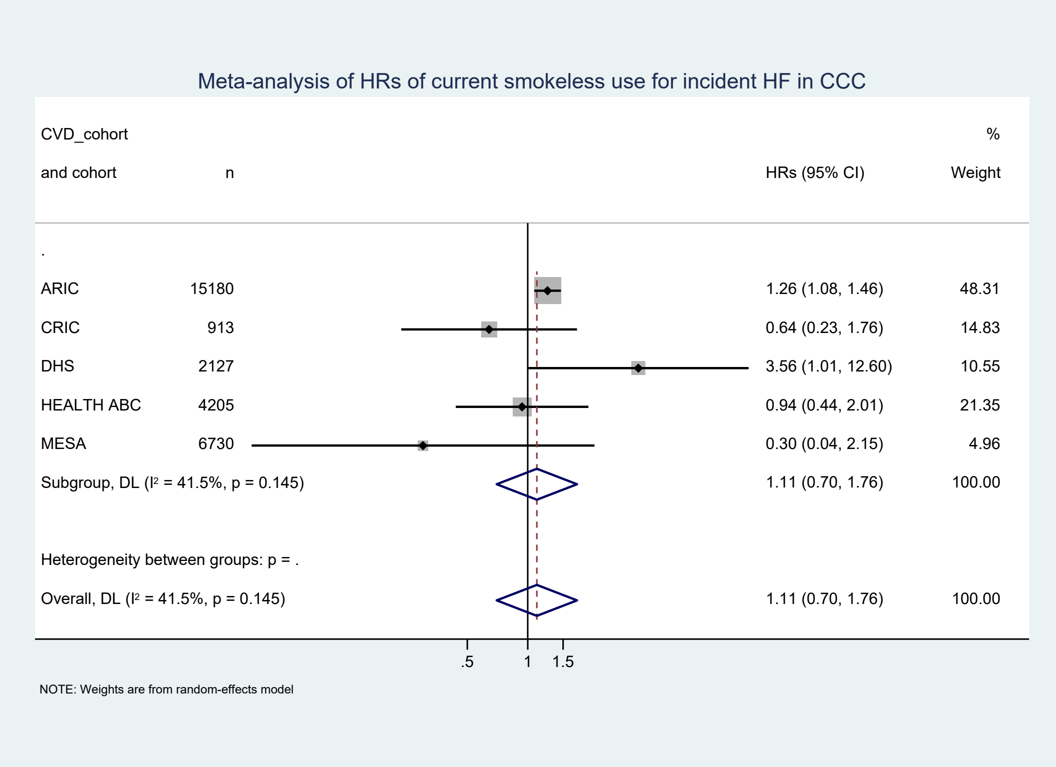

© 2025 Tasdighi E et al. *JAMA Network Open*.

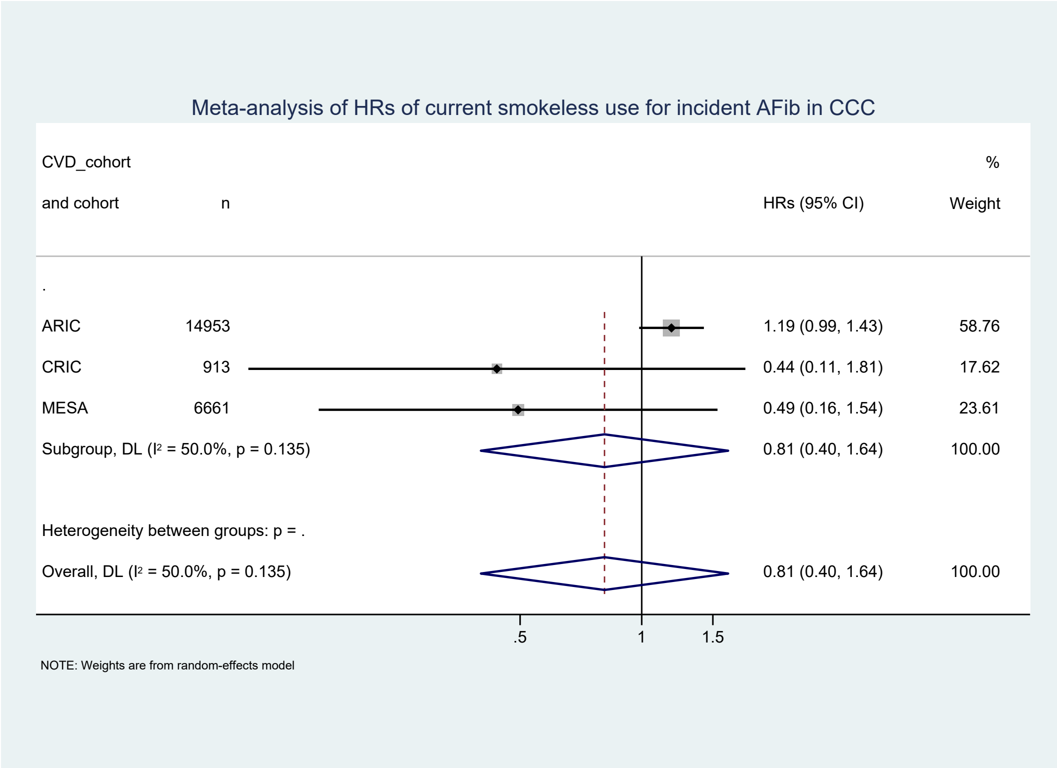

© 2025 Tasdighi E et al. *JAMA Network Open*.

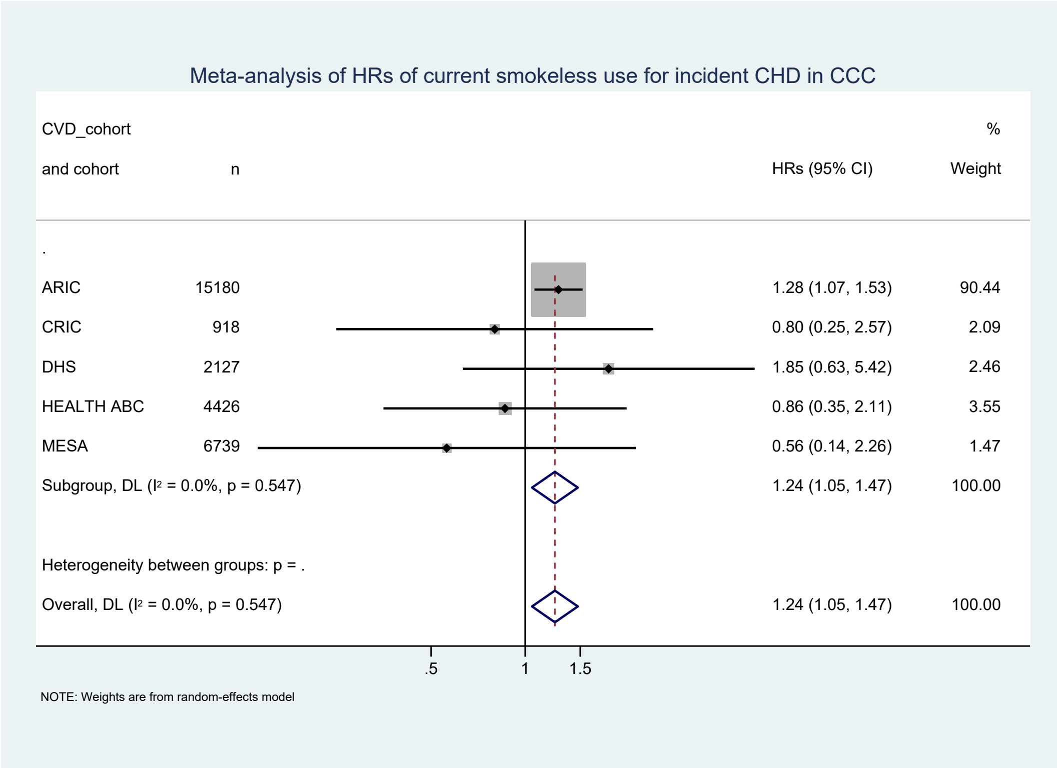

© 2025 Tasdighi E et al. *JAMA Network Open*.

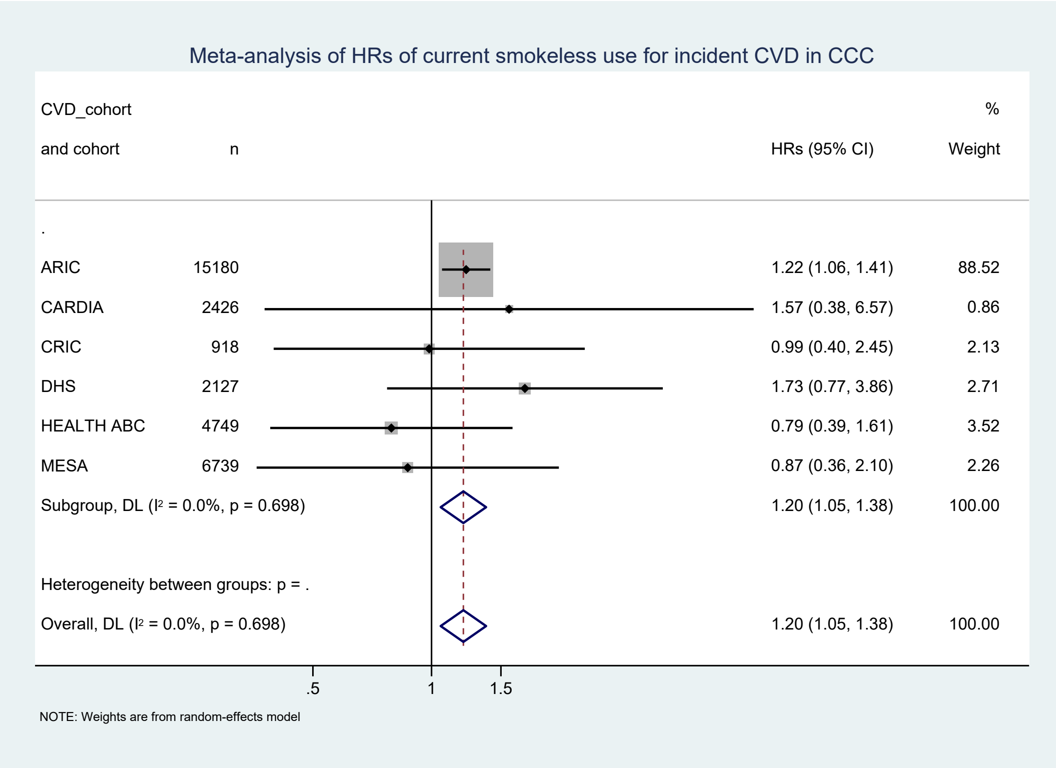

© 2025 Tasdighi E et al. *JAMA Network Open*.

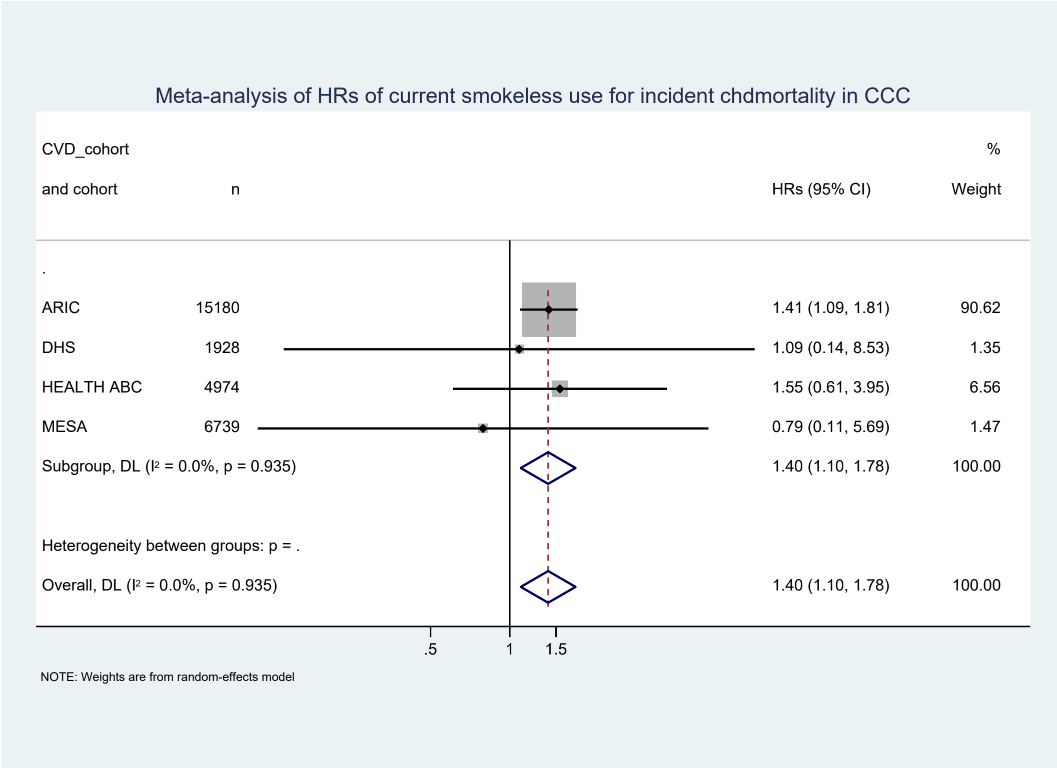

© 2025 Tasdighi E et al. *JAMA Network Open*.

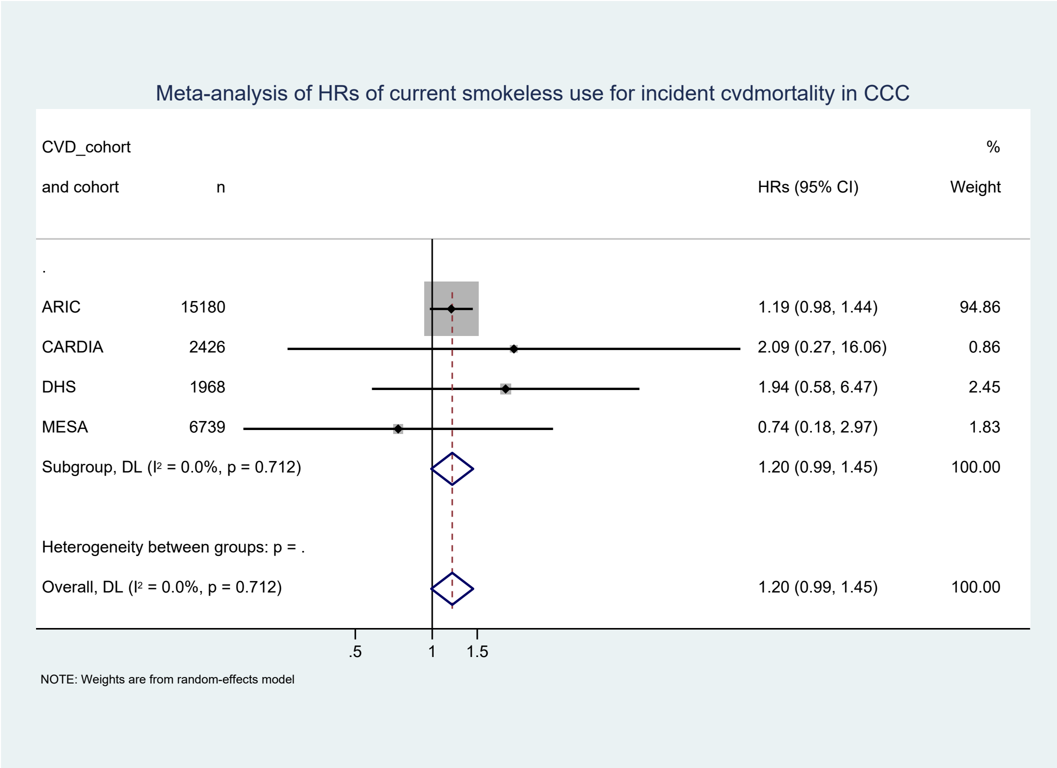

© 2025 Tasdighi E et al. *JAMA Network Open*.

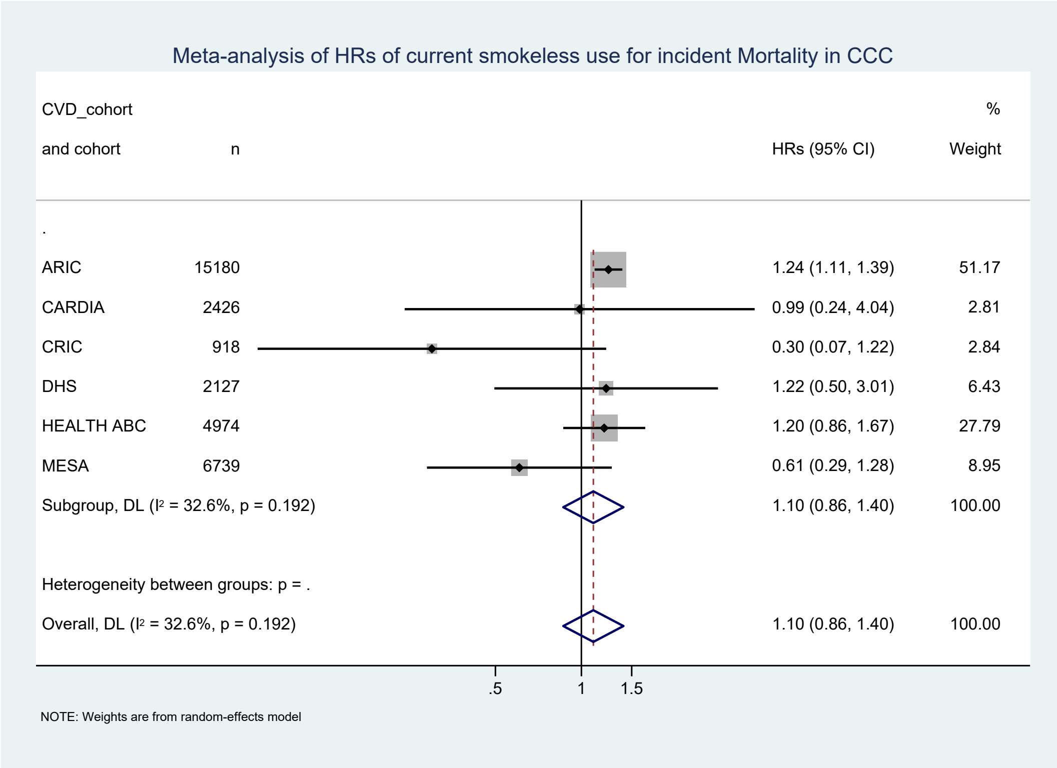

Smoking combustible cigarettes and health outcomes in each cohort

© 2025 Tasdighi E et al. *JAMA Network Open*.

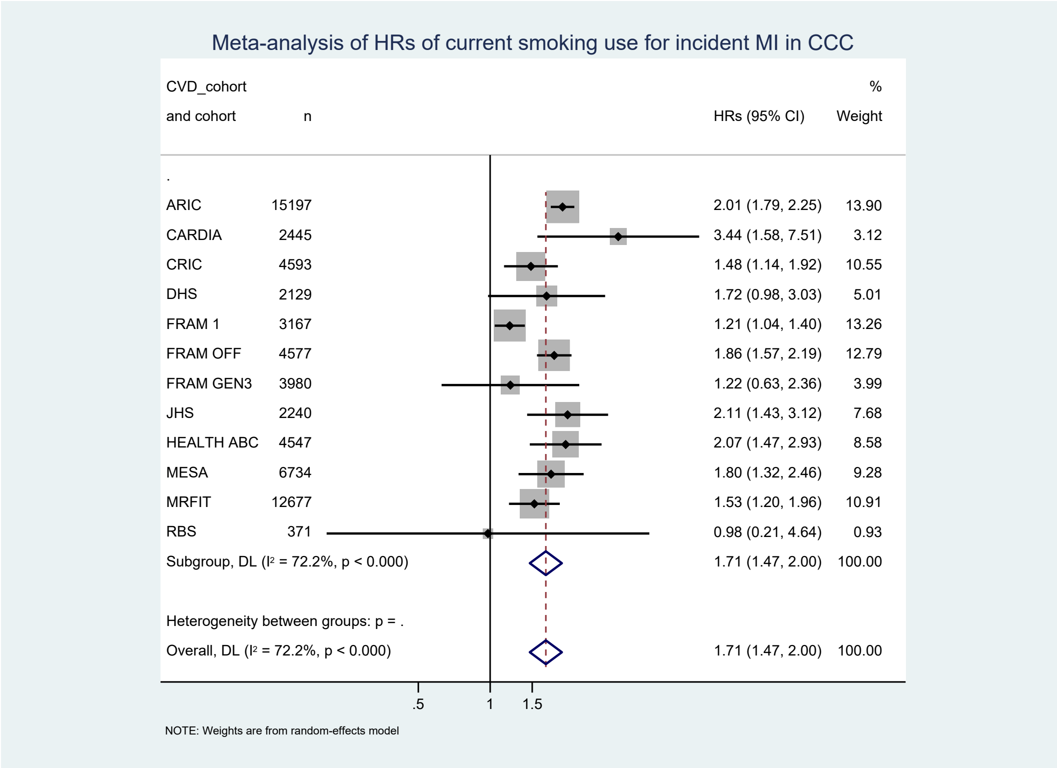

© 2025 Tasdighi E et al. *JAMA Network Open*.

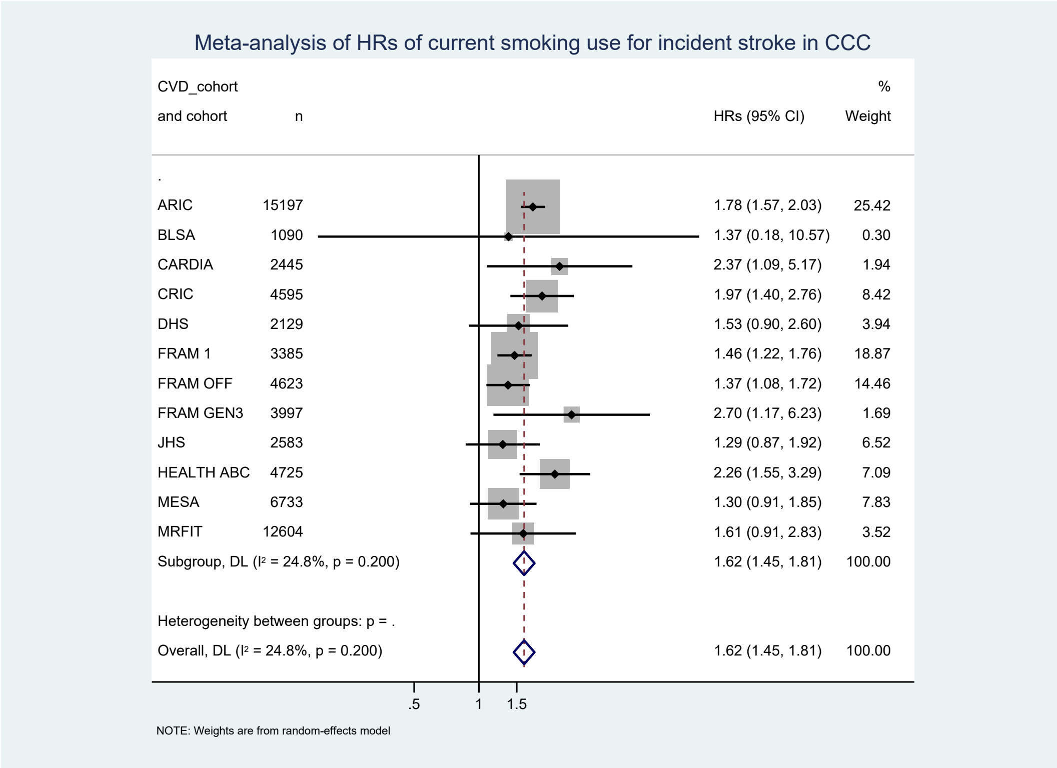

© 2025 Tasdighi E et al. *JAMA Network Open*.

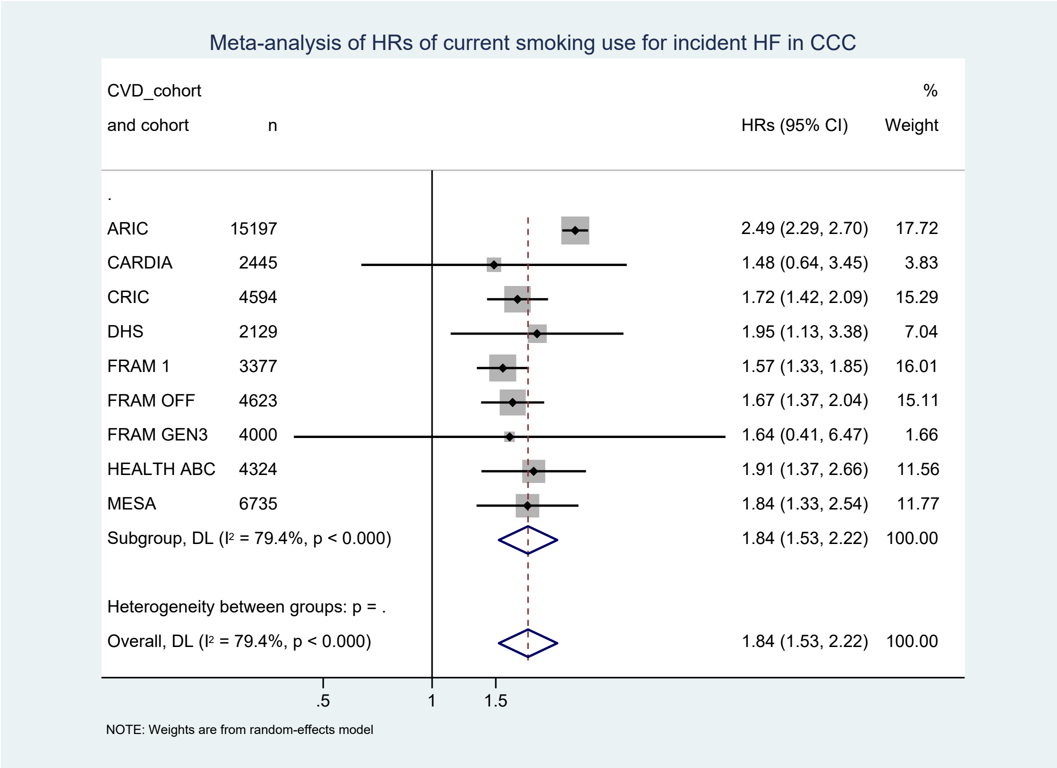

© 2025 Tasdighi E et al. *JAMA Network Open*.

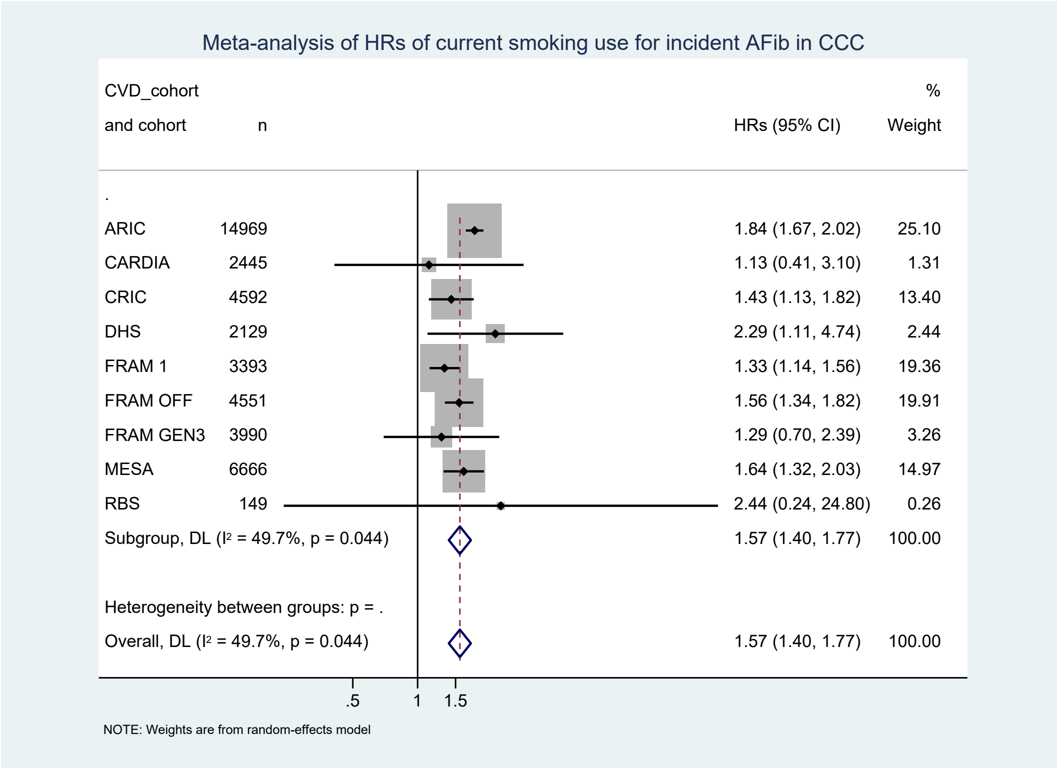

© 2025 Tasdighi E et al. *JAMA Network Open*.

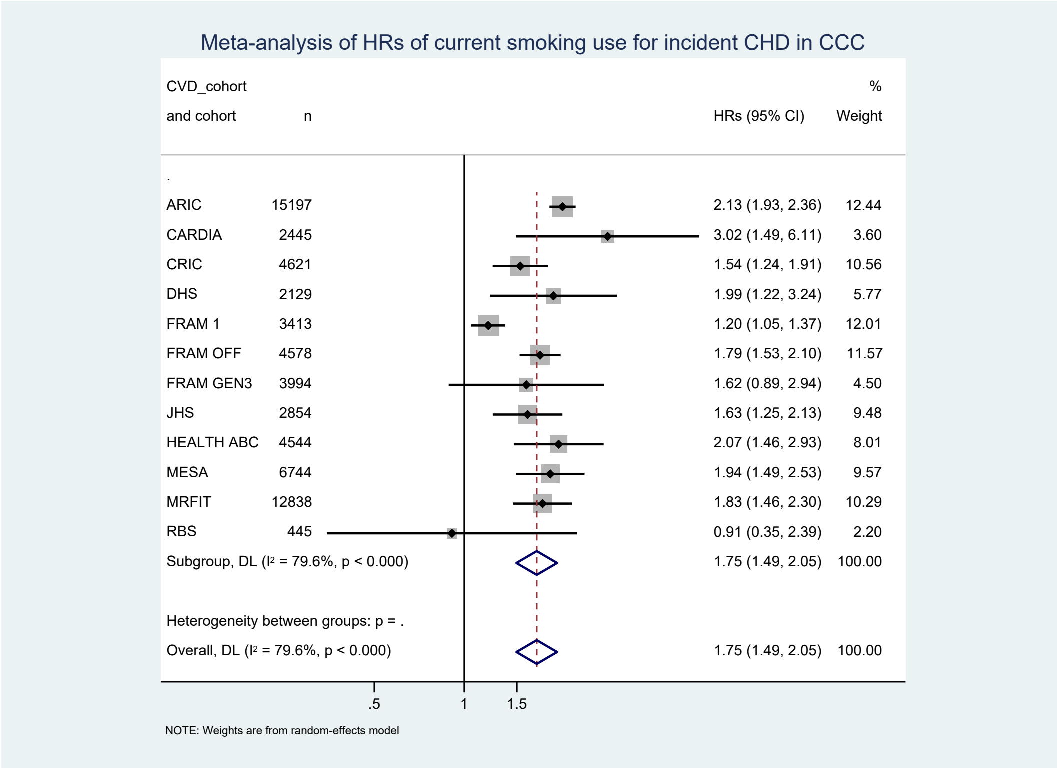

© 2025 Tasdighi E et al. *JAMA Network Open*.

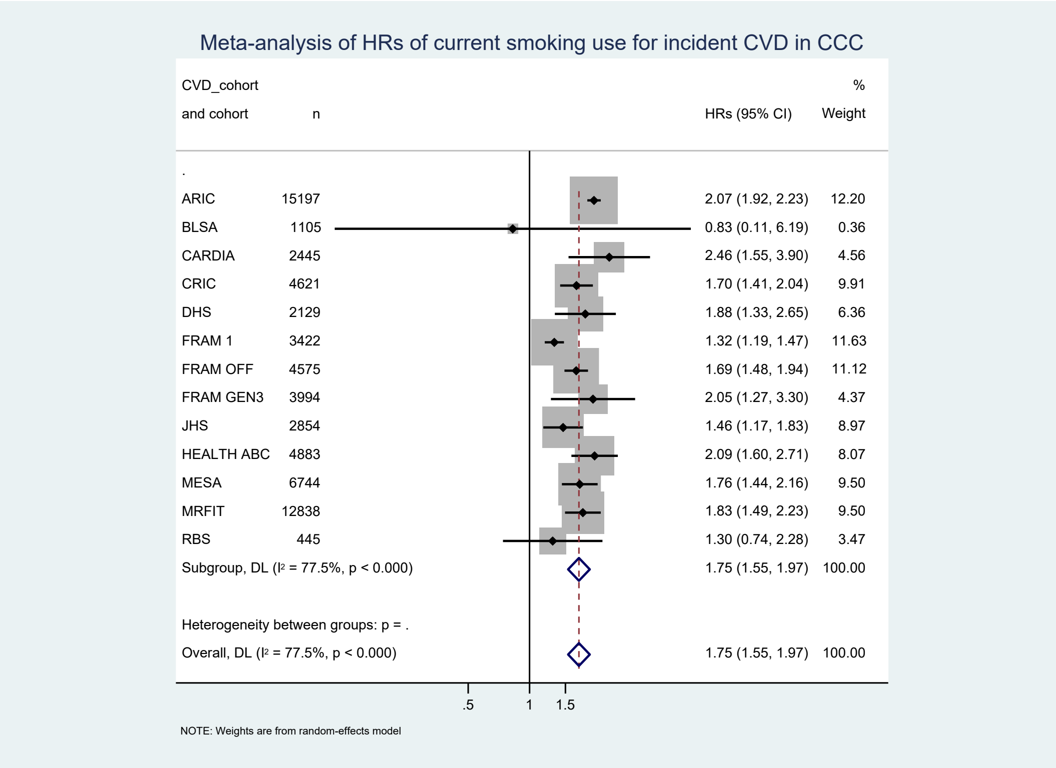

© 2025 Tasdighi E et al. *JAMA Network Open*.

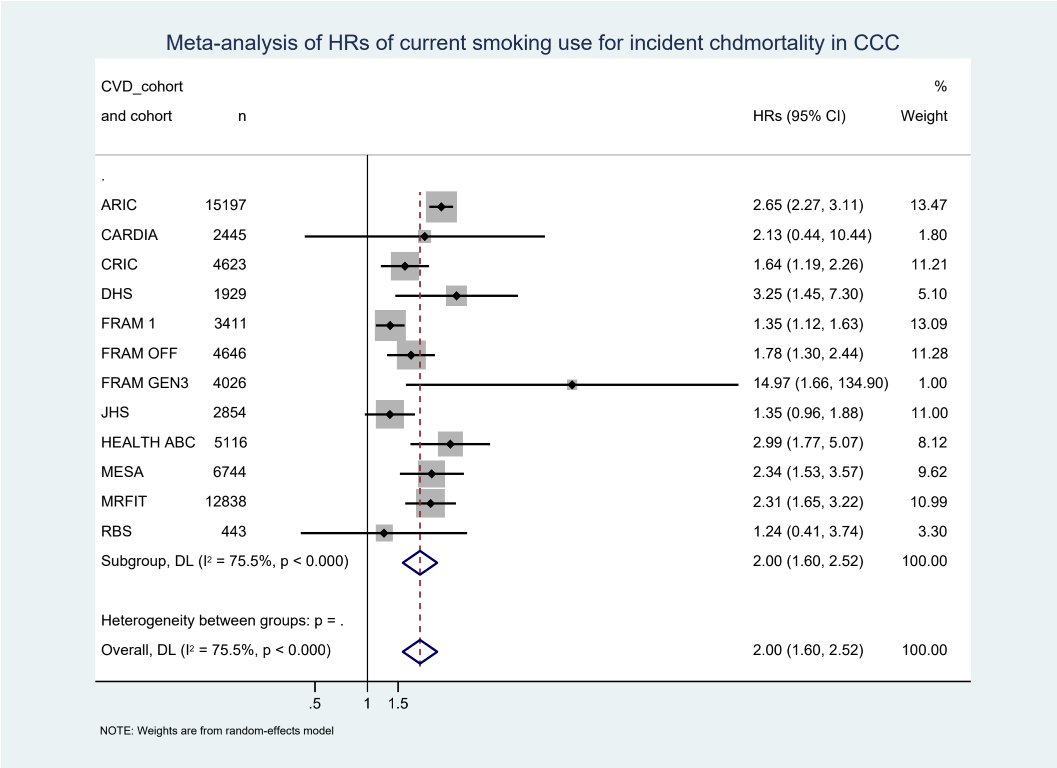

© 2025 Tasdighi E et al. *JAMA Network Open*.

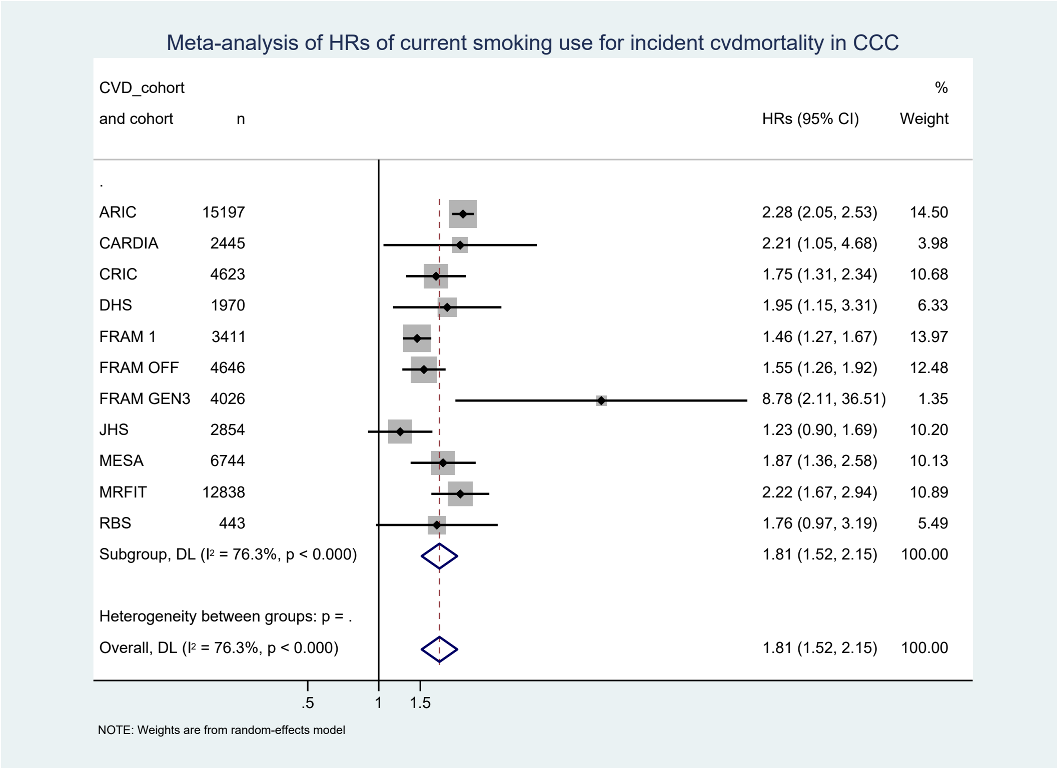

© 2025 Tasdighi E et al. *JAMA Network Open*.

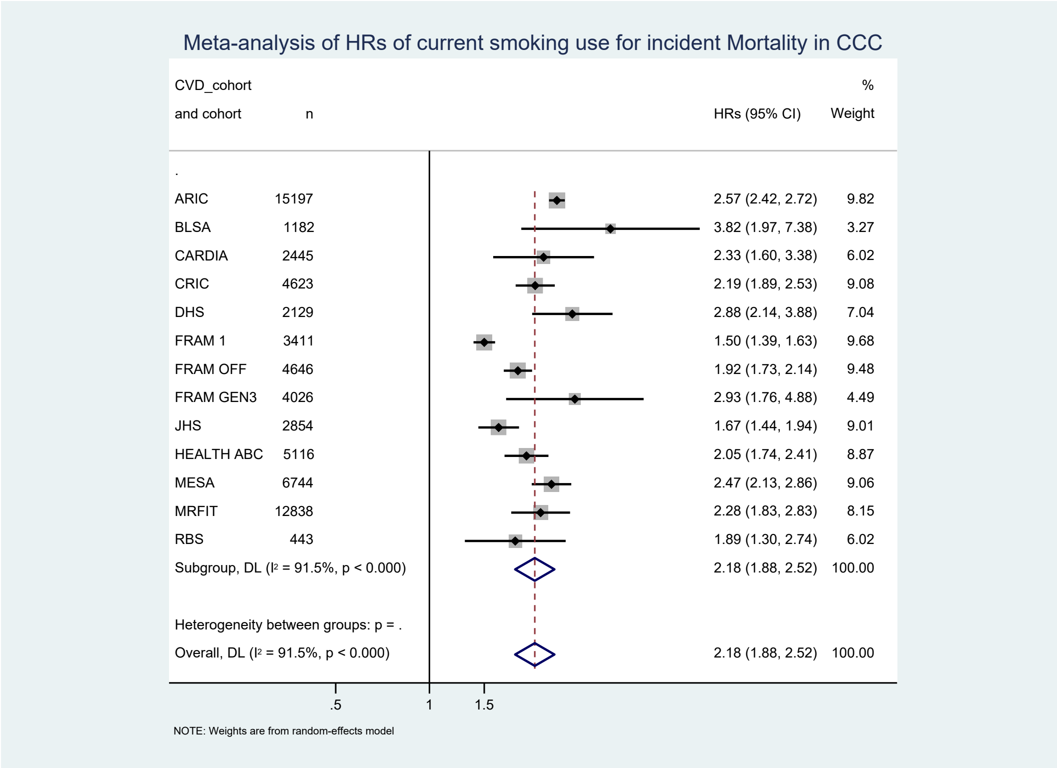

Cigar sole use and health outcomes in each cohort:

© 2025 Tasdighi E et al. *JAMA Network Open*.

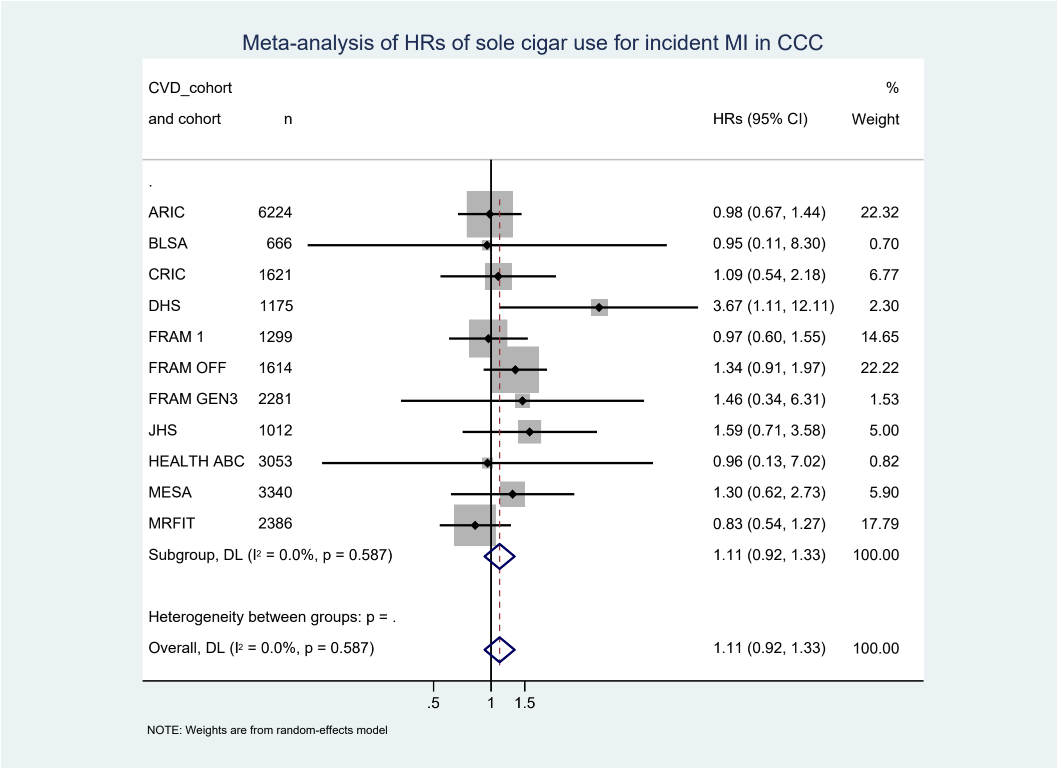

© 2025 Tasdighi E et al. *JAMA Network Open*.

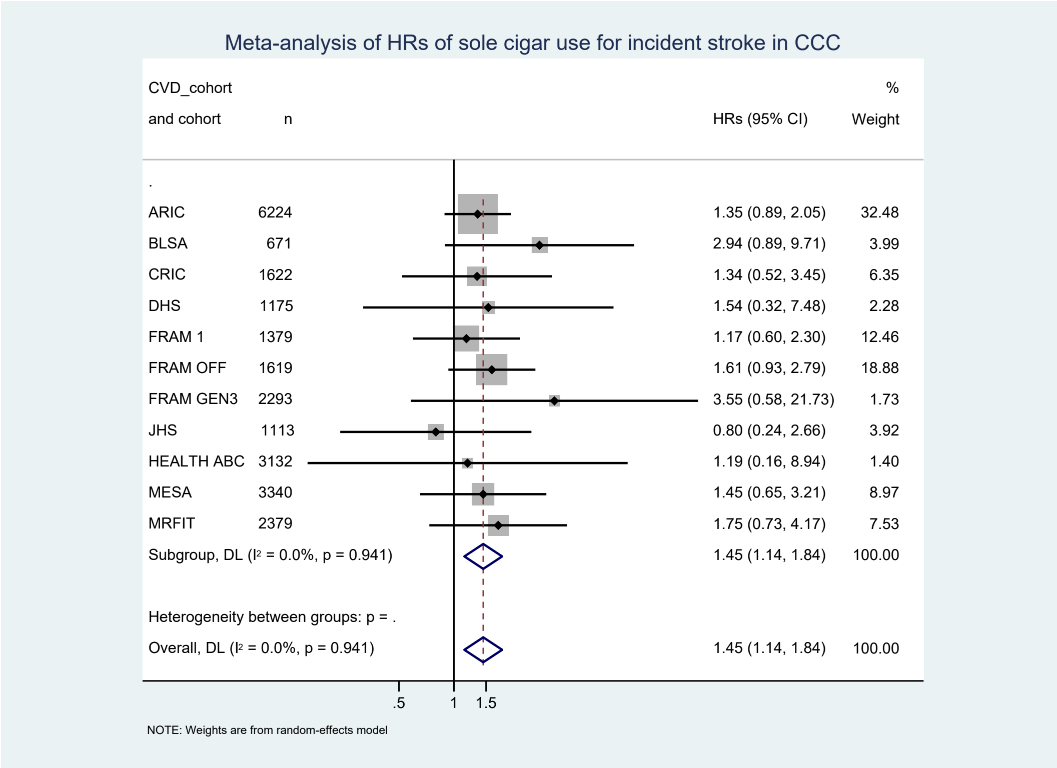

© 2025 Tasdighi E et al. *JAMA Network Open*.

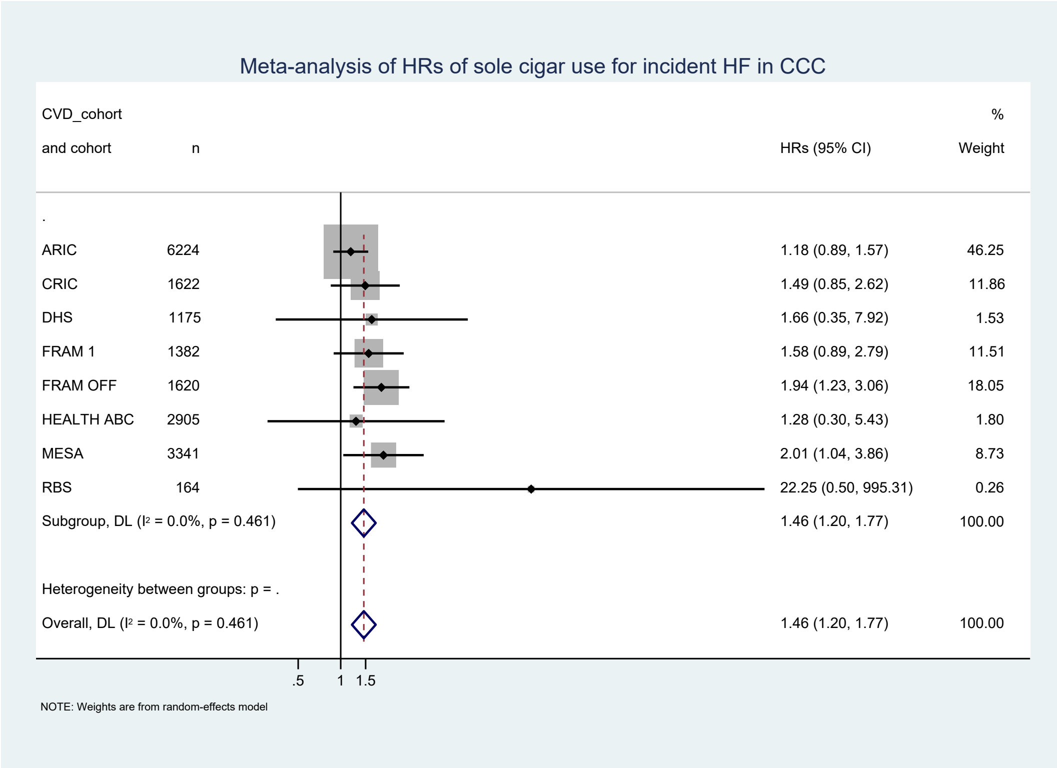

© 2025 Tasdighi E et al. *JAMA Network Open*.

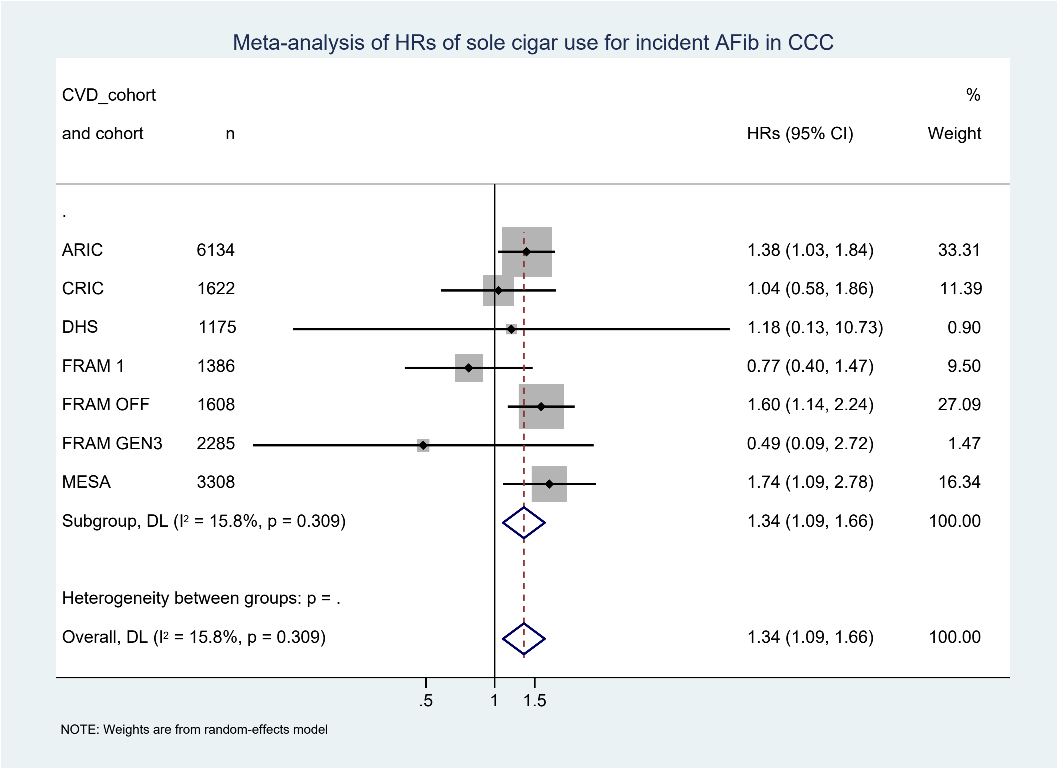

© 2025 Tasdighi E et al. *JAMA Network Open*.

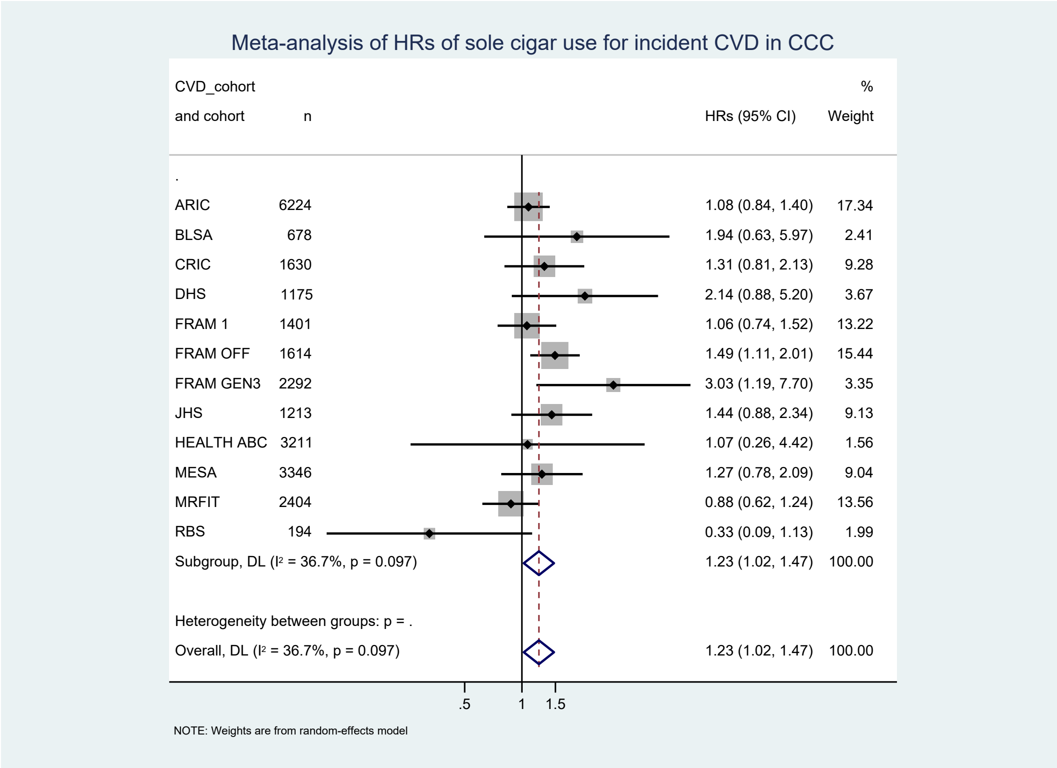

© 2025 Tasdighi E et al. *JAMA Network Open*.

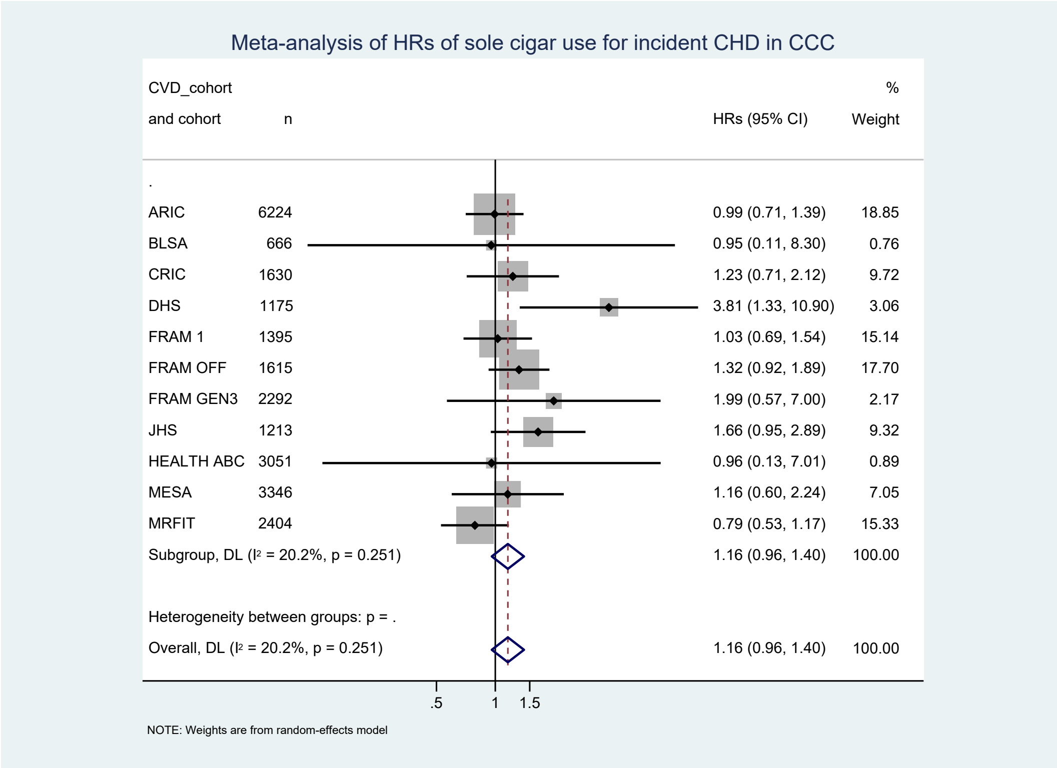

© 2025 Tasdighi E et al. *JAMA Network Open*.

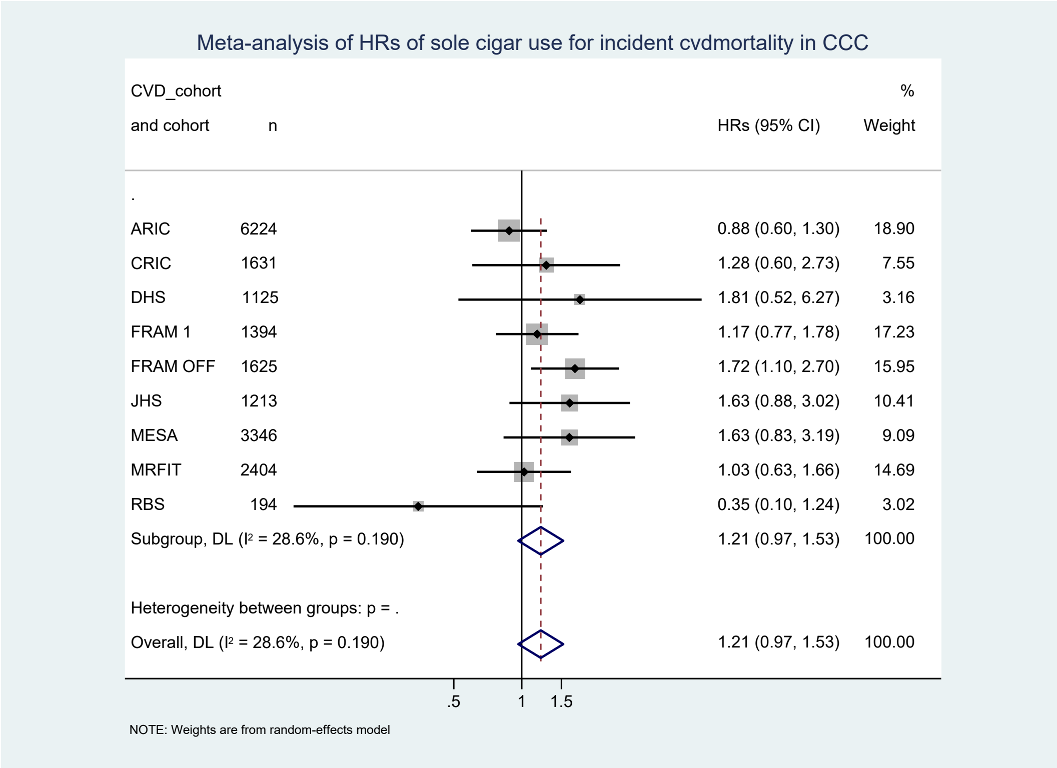

© 2025 Tasdighi E et al. *JAMA Network Open*.

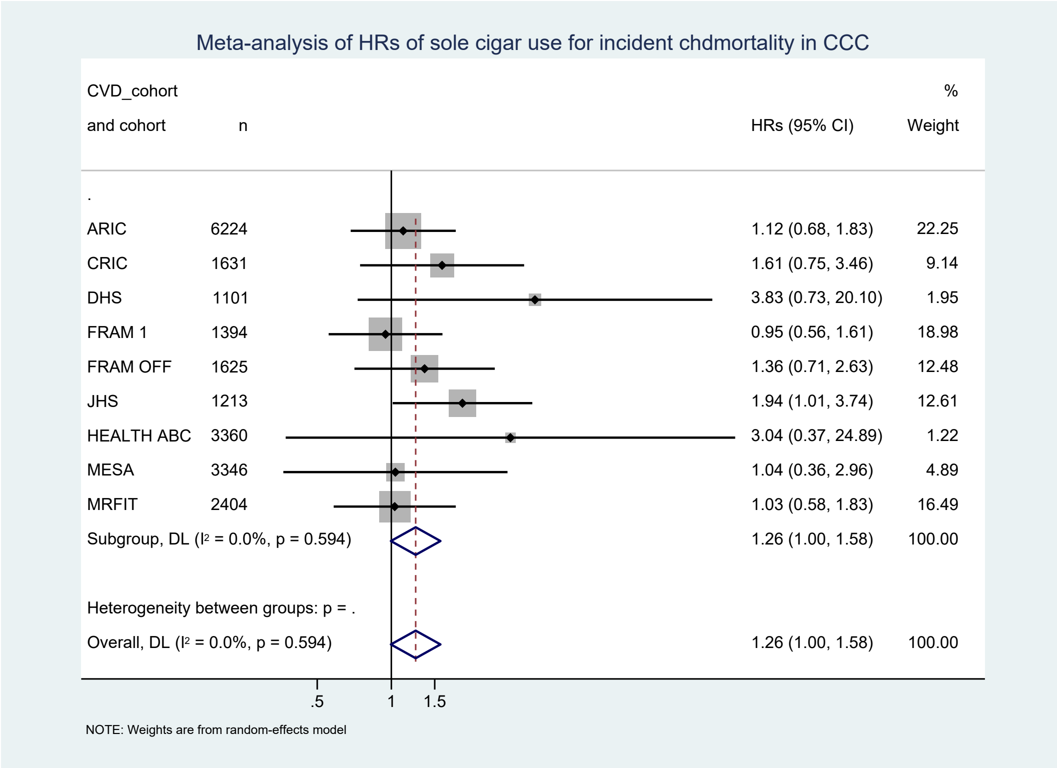

© 2025 Tasdighi E et al. *JAMA Network Open*.

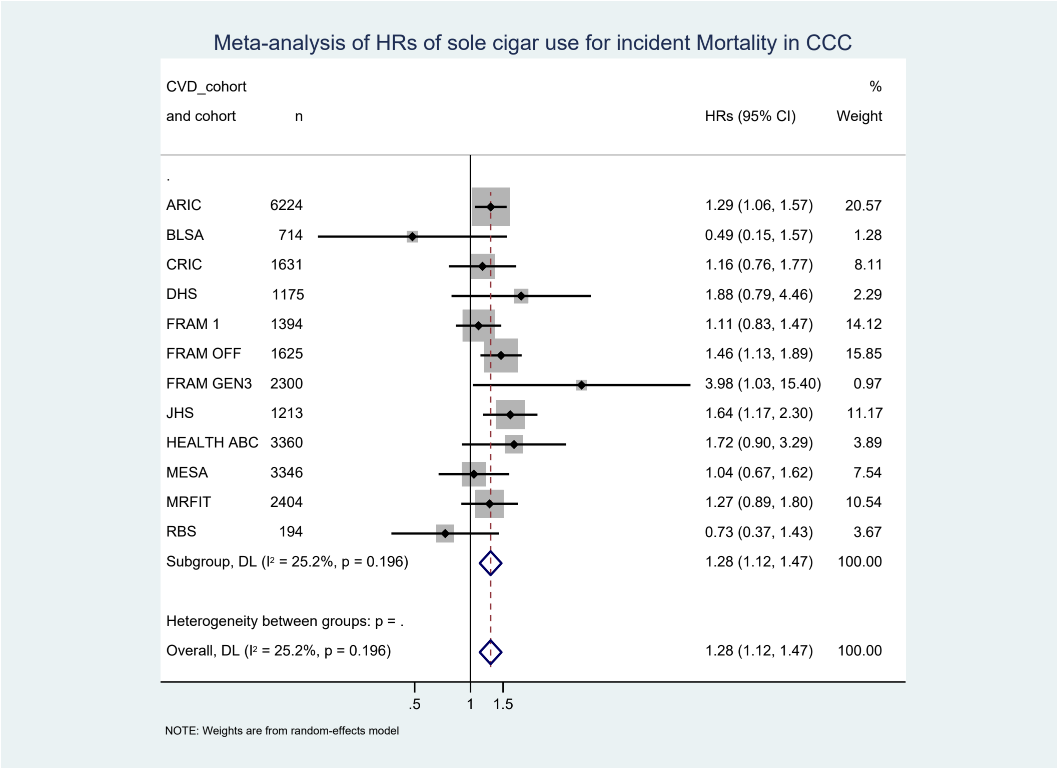

© 2025 Tasdighi E et al. *JAMA Network Open*.

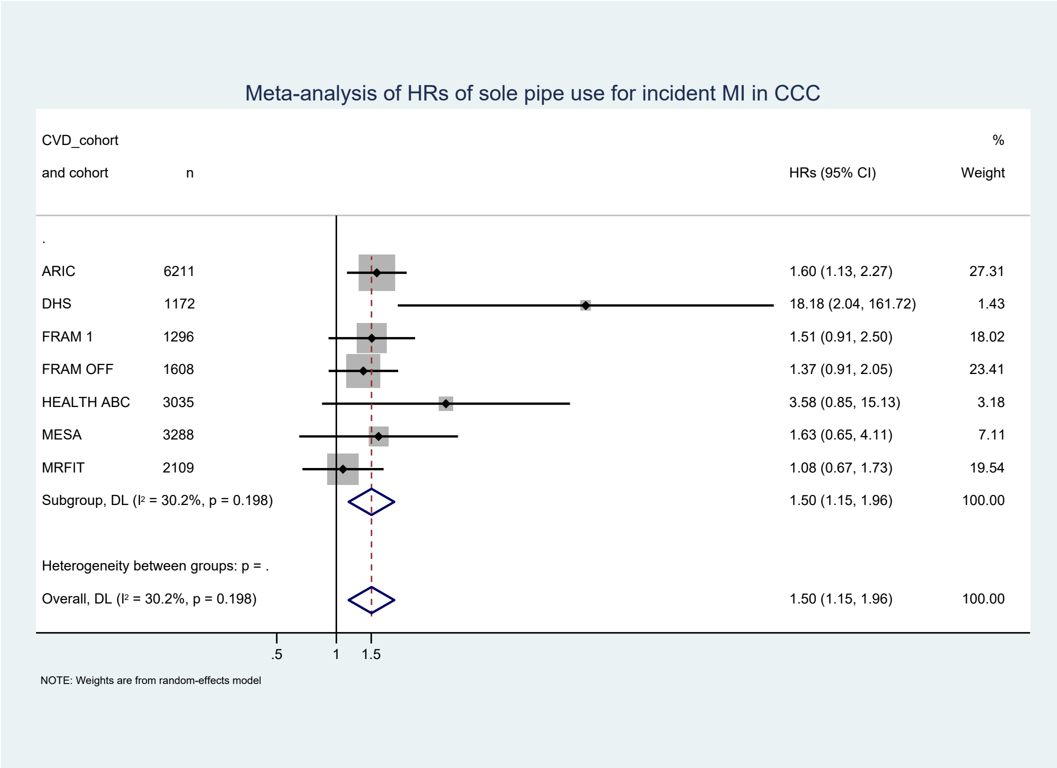

© 2025 Tasdighi E et al. *JAMA Network Open*.

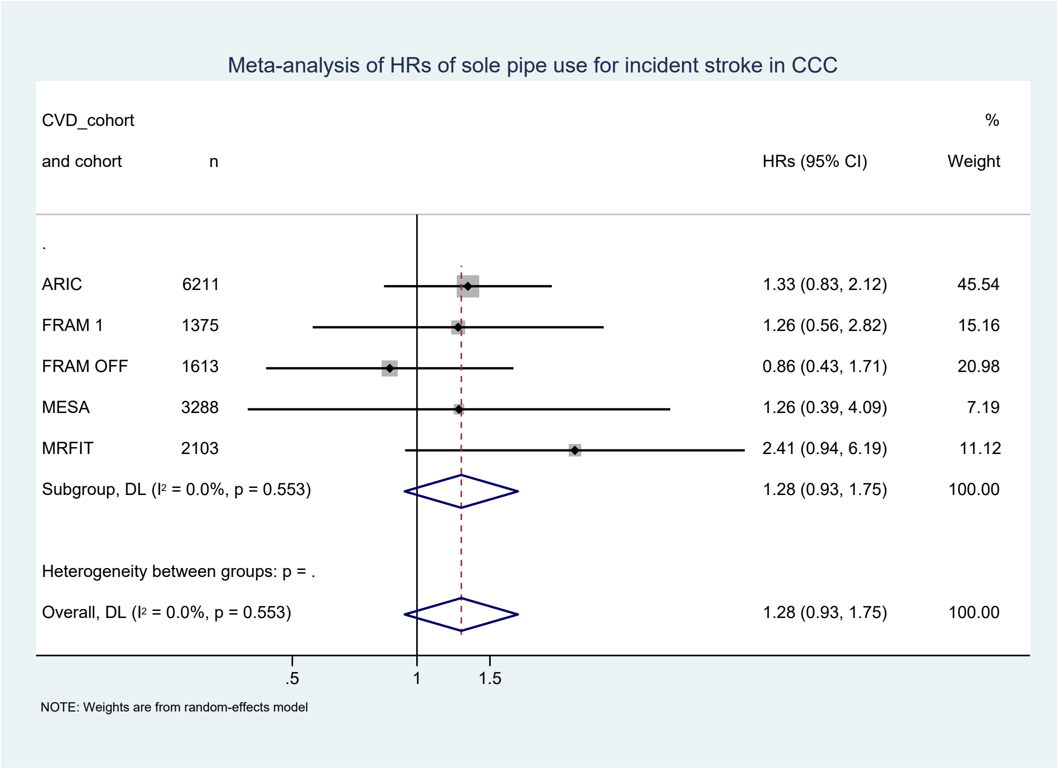

© 2025 Tasdighi E et al. *JAMA Network Open*.

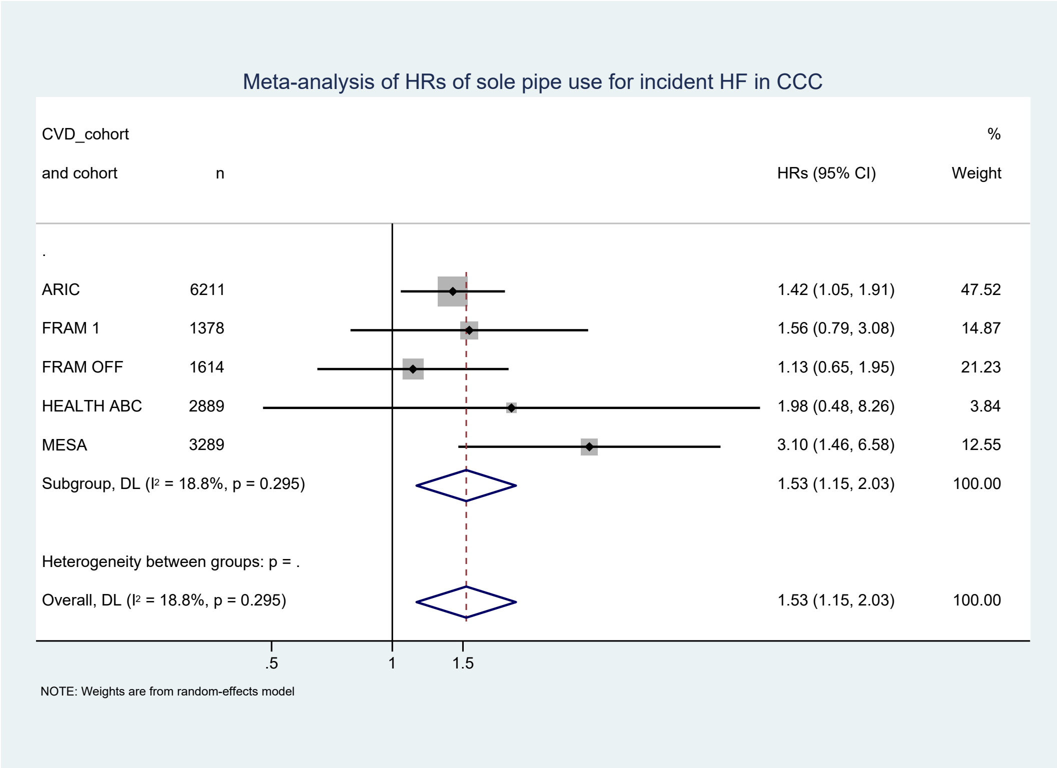

© 2025 Tasdighi E et al. *JAMA Network Open*.

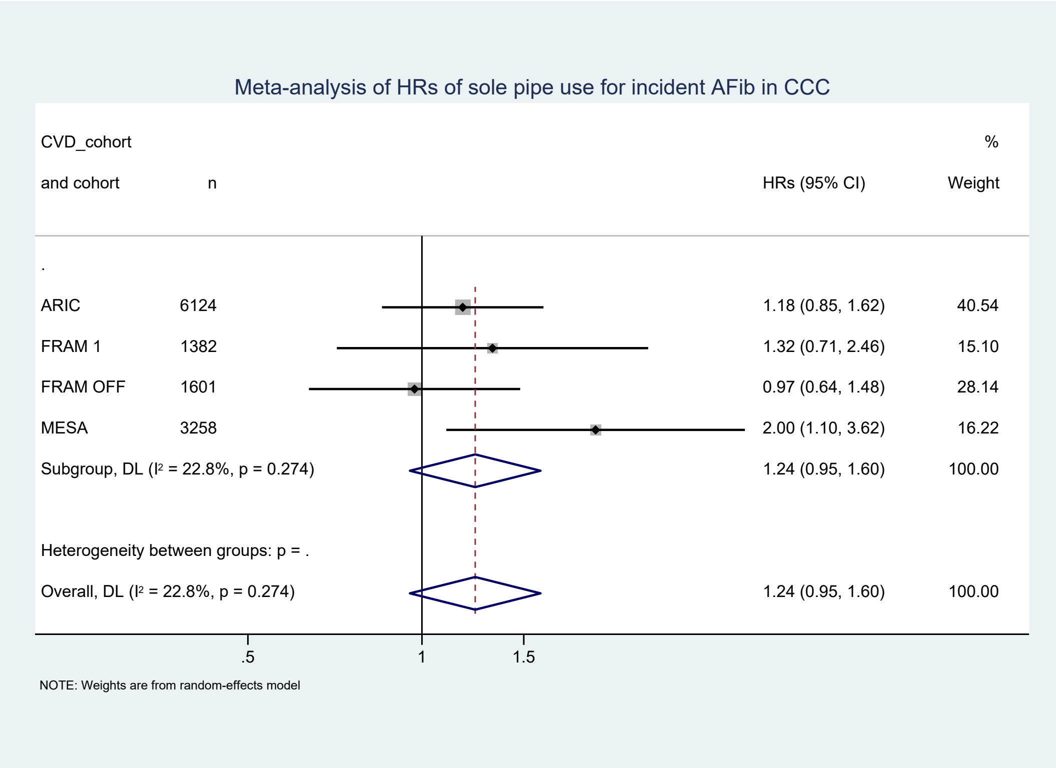

© 2025 Tasdighi E et al. *JAMA Network Open*.

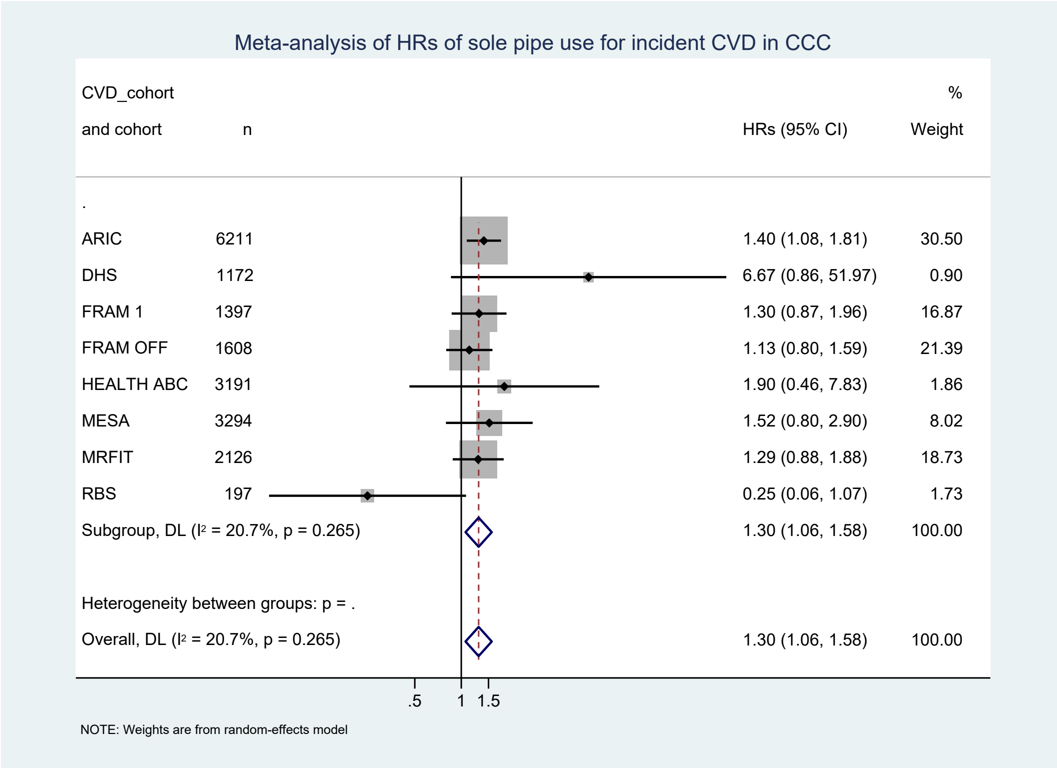

© 2025 Tasdighi E et al. *JAMA Network Open*.

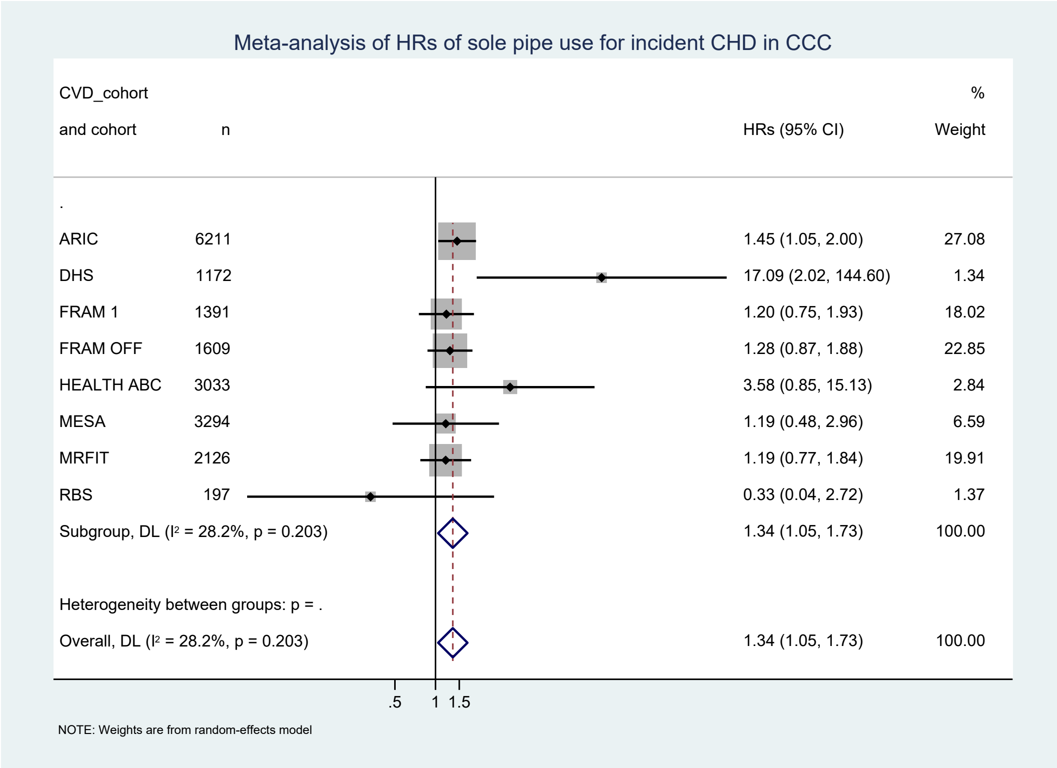

© 2025 Tasdighi E et al. *JAMA Network Open*.

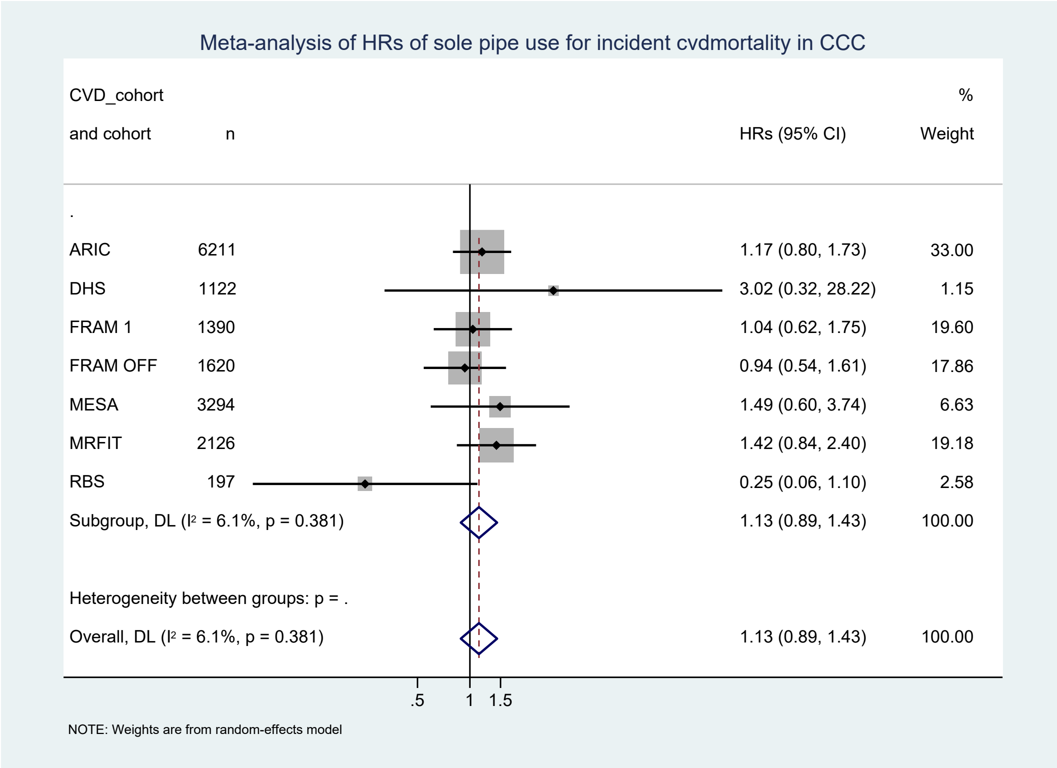

© 2025 Tasdighi E et al. *JAMA Network Open*.

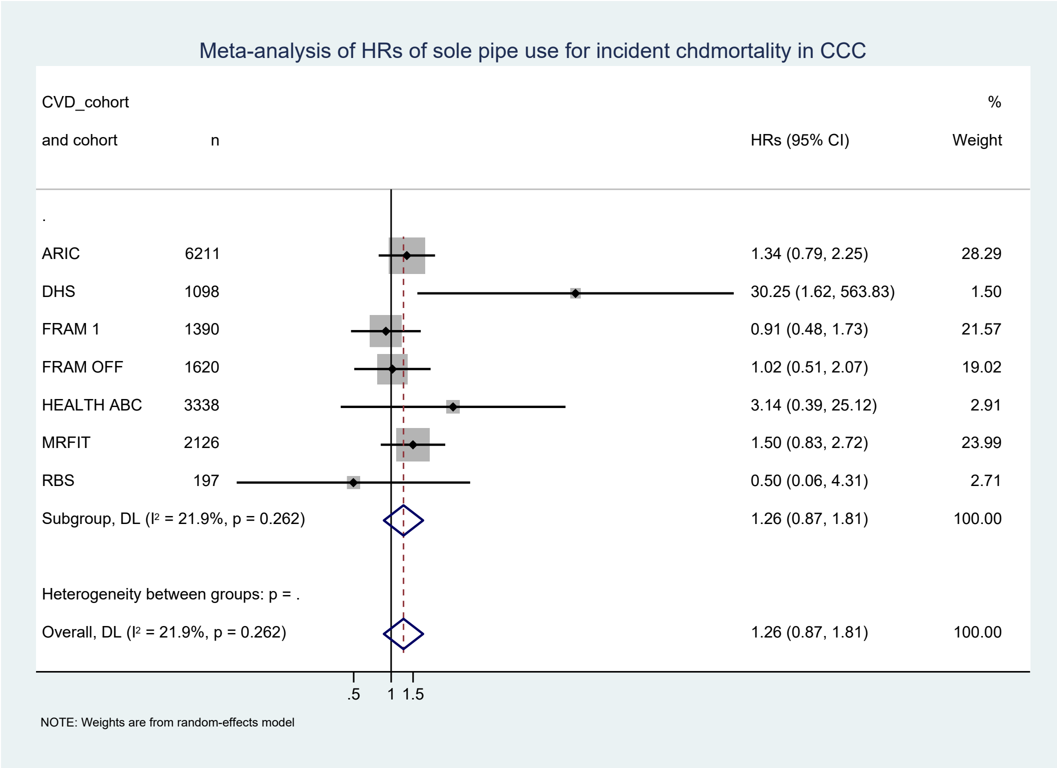

© 2025 Tasdighi E et al. *JAMA Network Open*.

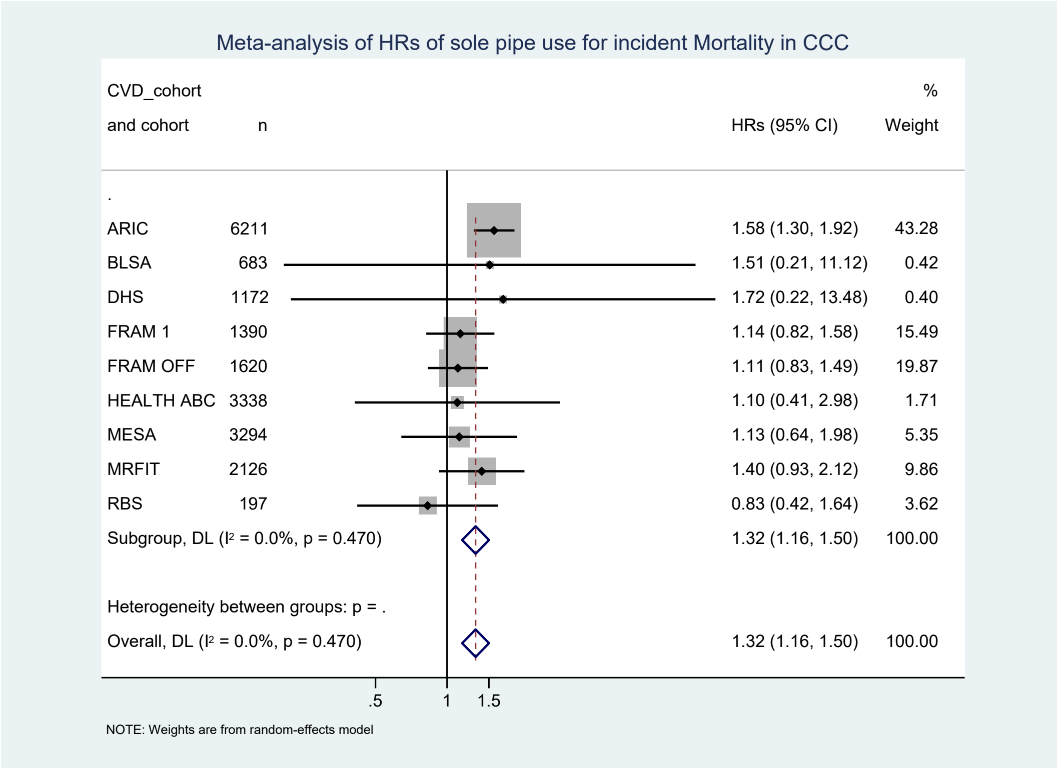

© 2025 Tasdighi E et al. *JAMA Network Open*.

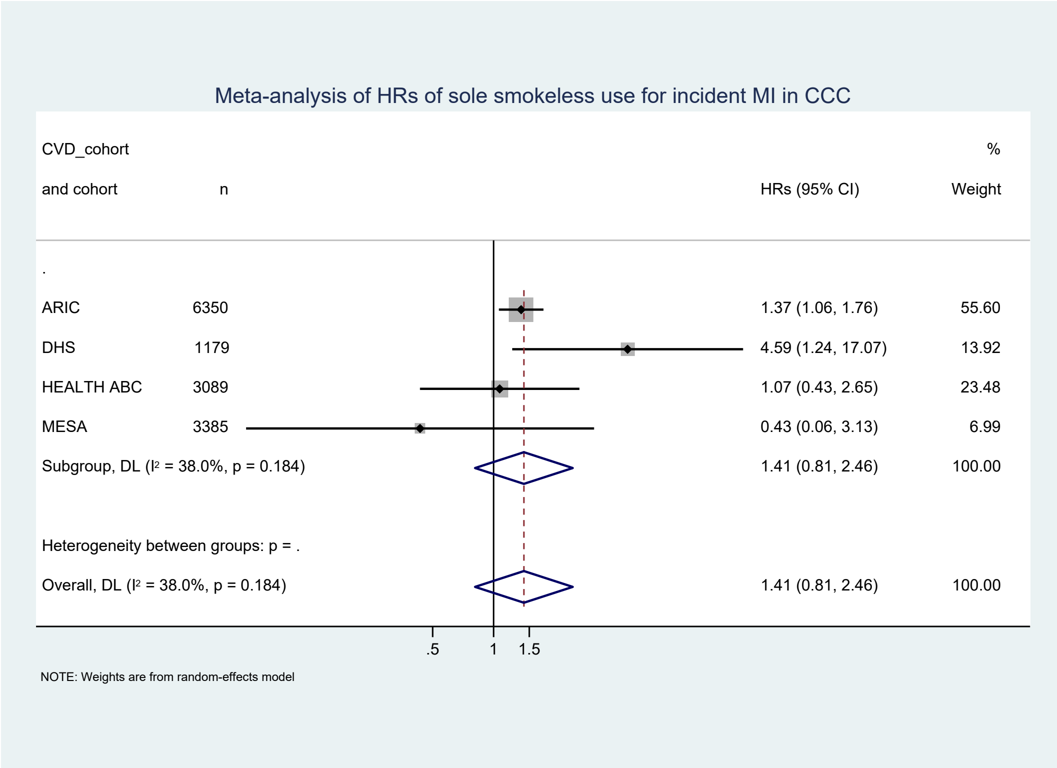

© 2025 Tasdighi E et al. *JAMA Network Open*.

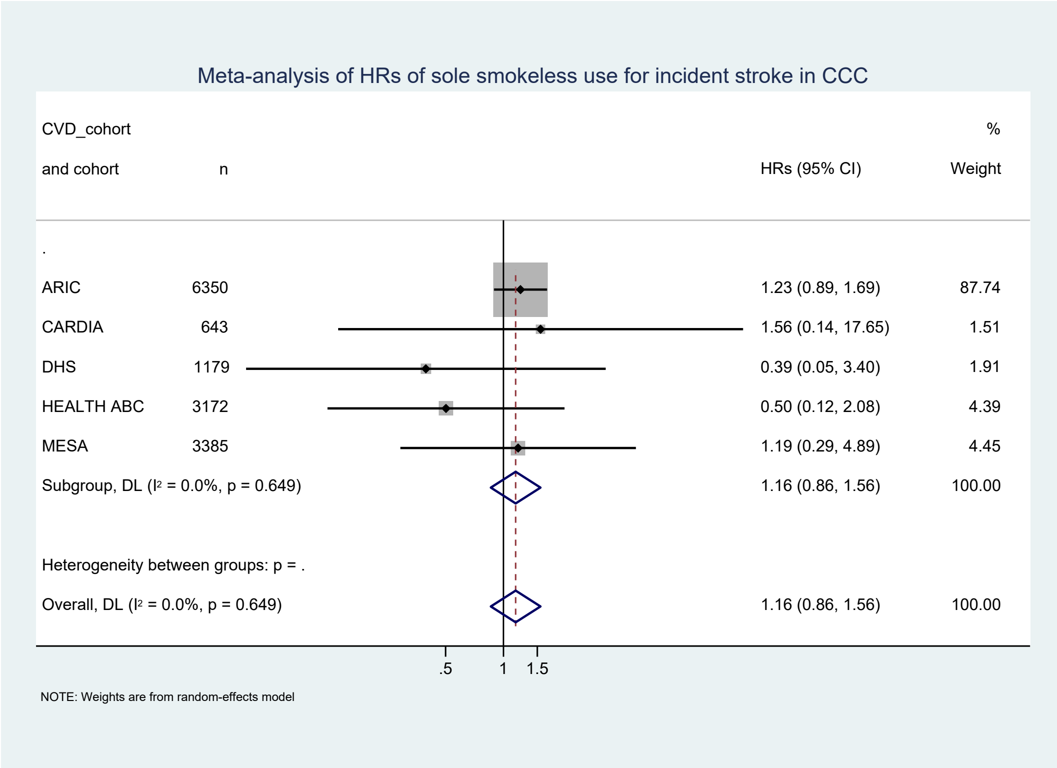

© 2025 Tasdighi E et al. *JAMA Network Open*.

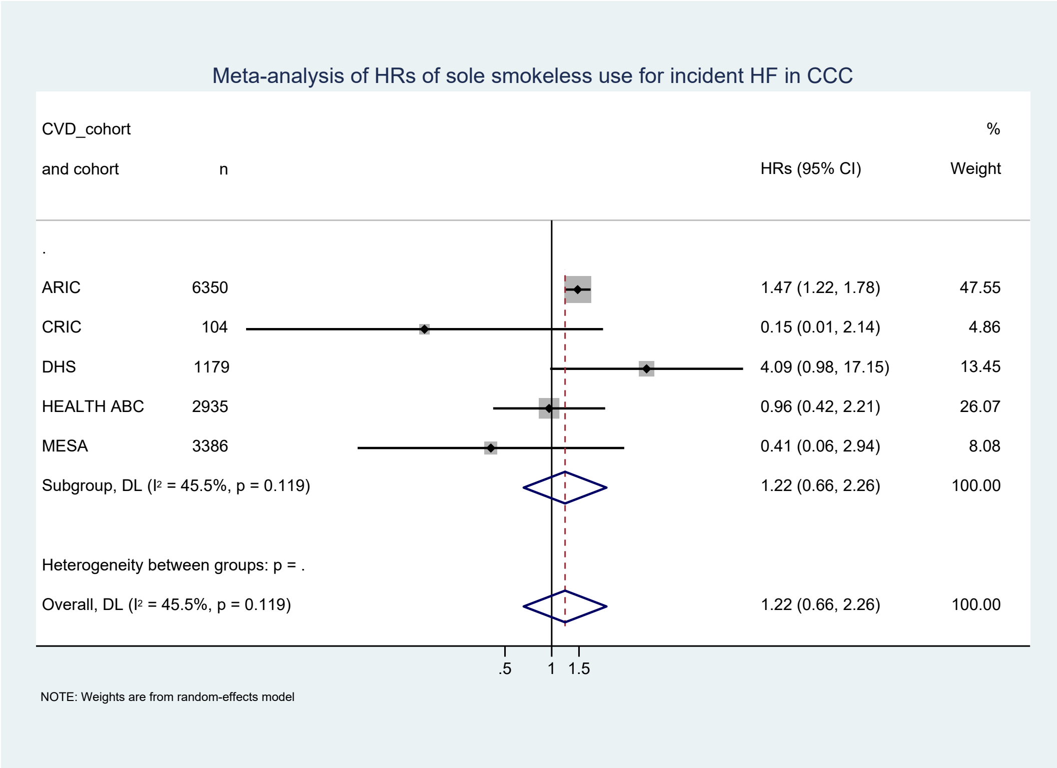

© 2025 Tasdighi E et al. *JAMA Network Open*.

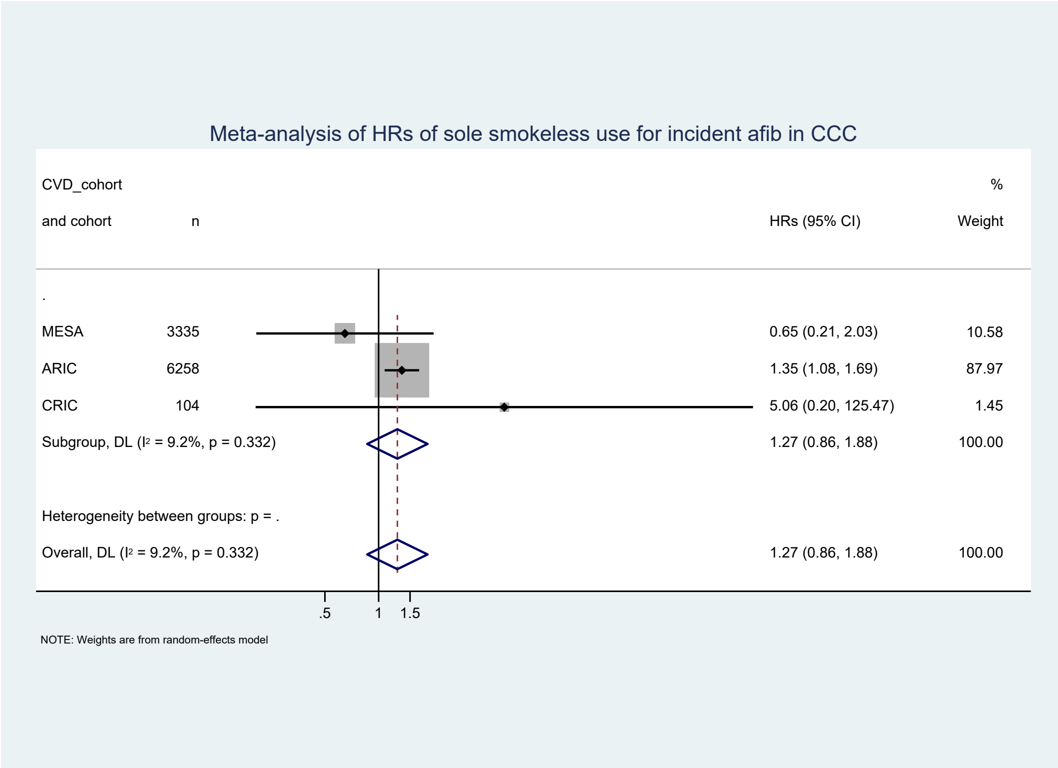

© 2025 Tasdighi E et al. *JAMA Network Open*.

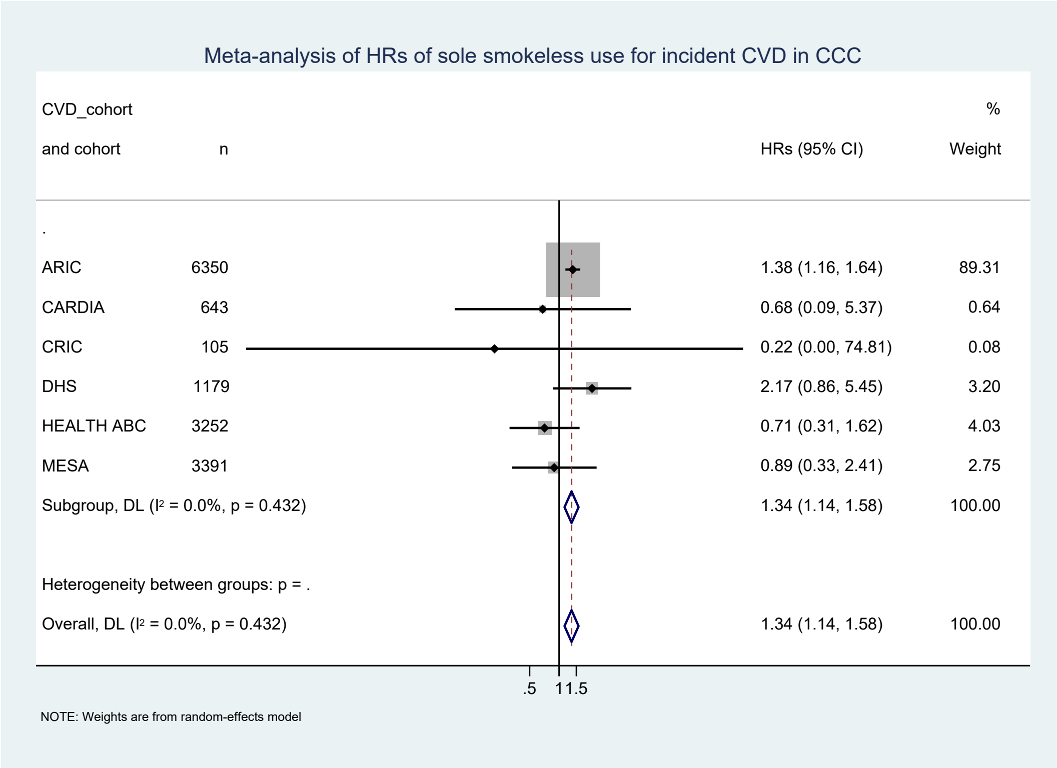

© 2025 Tasdighi E et al. *JAMA Network Open*.

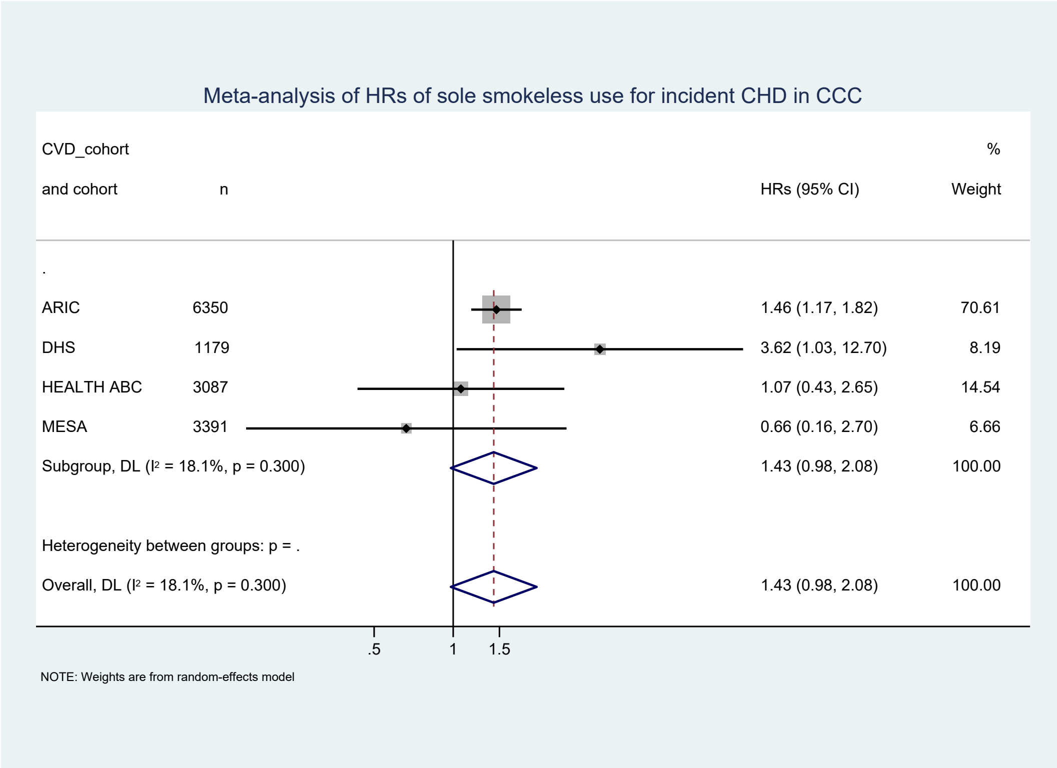

© 2025 Tasdighi E et al. *JAMA Network Open*.

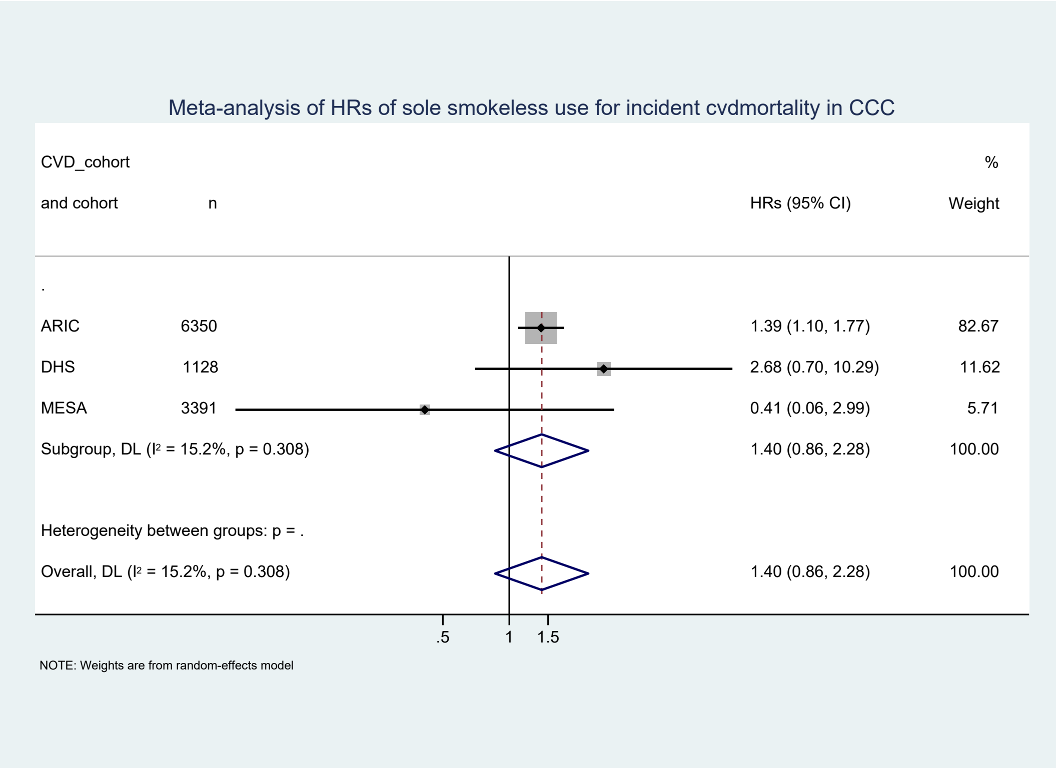

© 2025 Tasdighi E et al. *JAMA Network Open*.

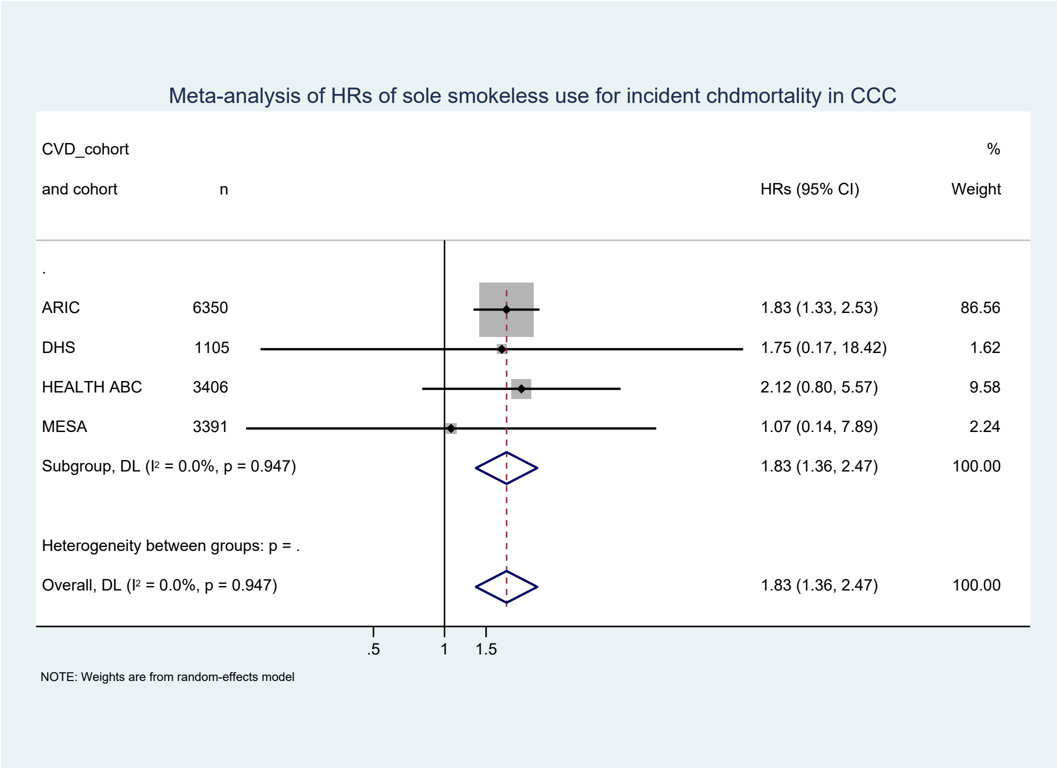

© 2025 Tasdighi E et al. *JAMA Network Open*.

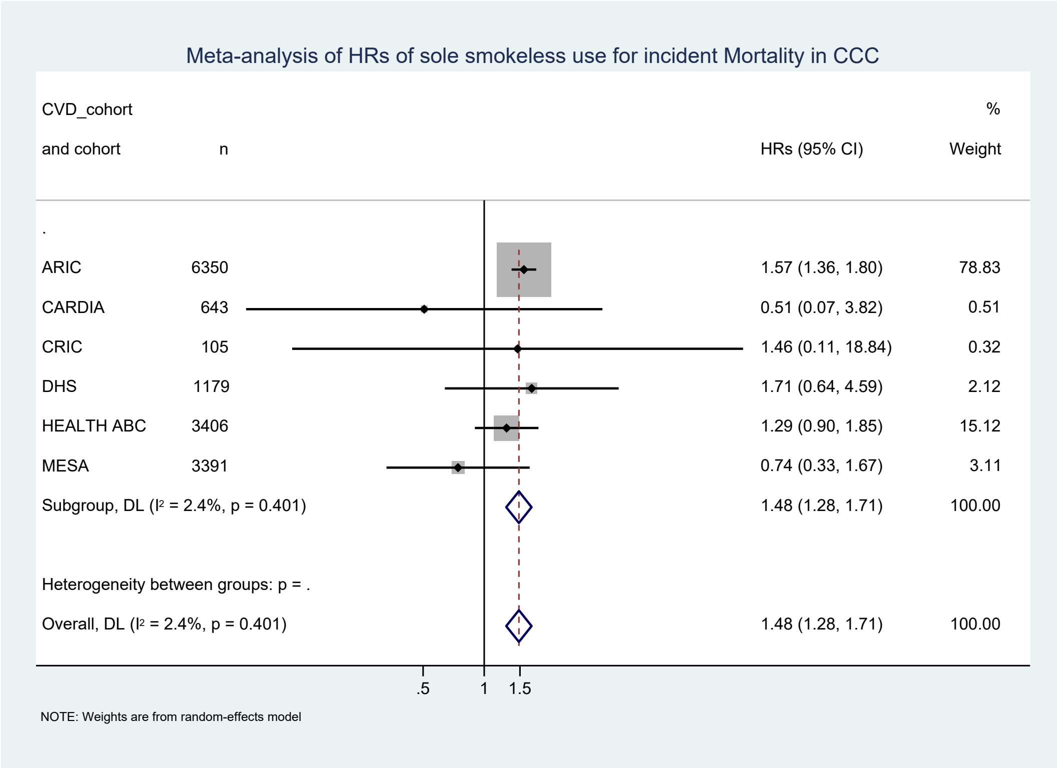

© 2025 Tasdighi E et al. *JAMA Network Open*.

Exclusive tobacco use and health outcomes in each cohort:

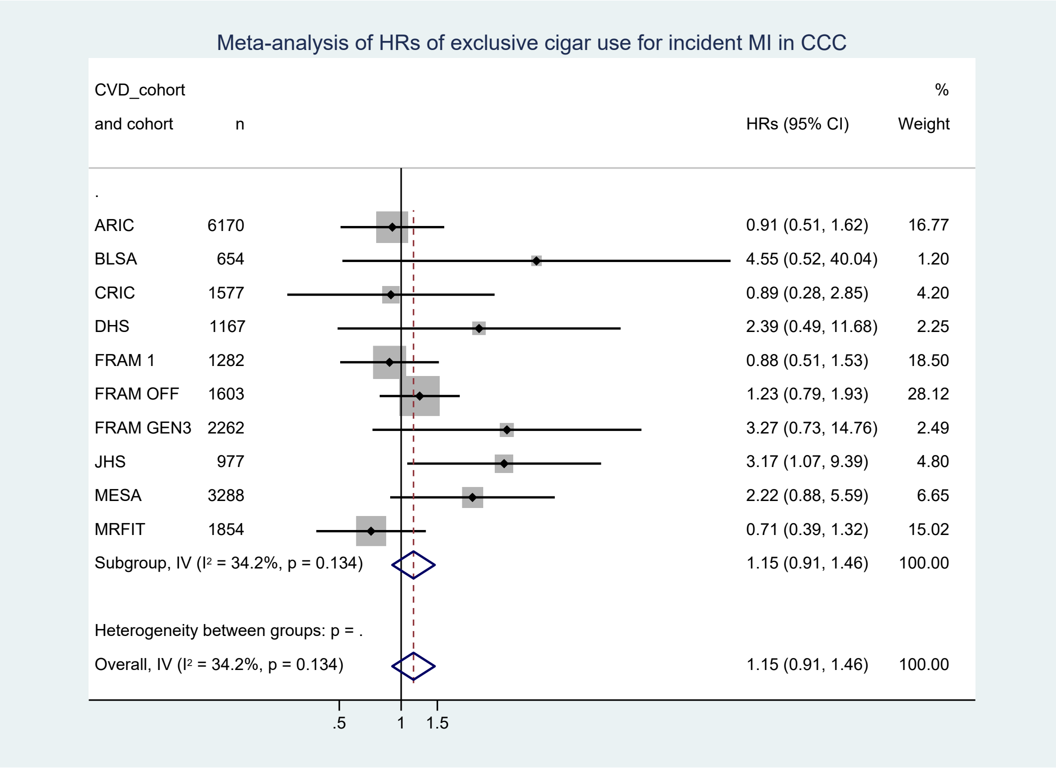

© 2025 Tasdighi E et al. *JAMA Network Open*.

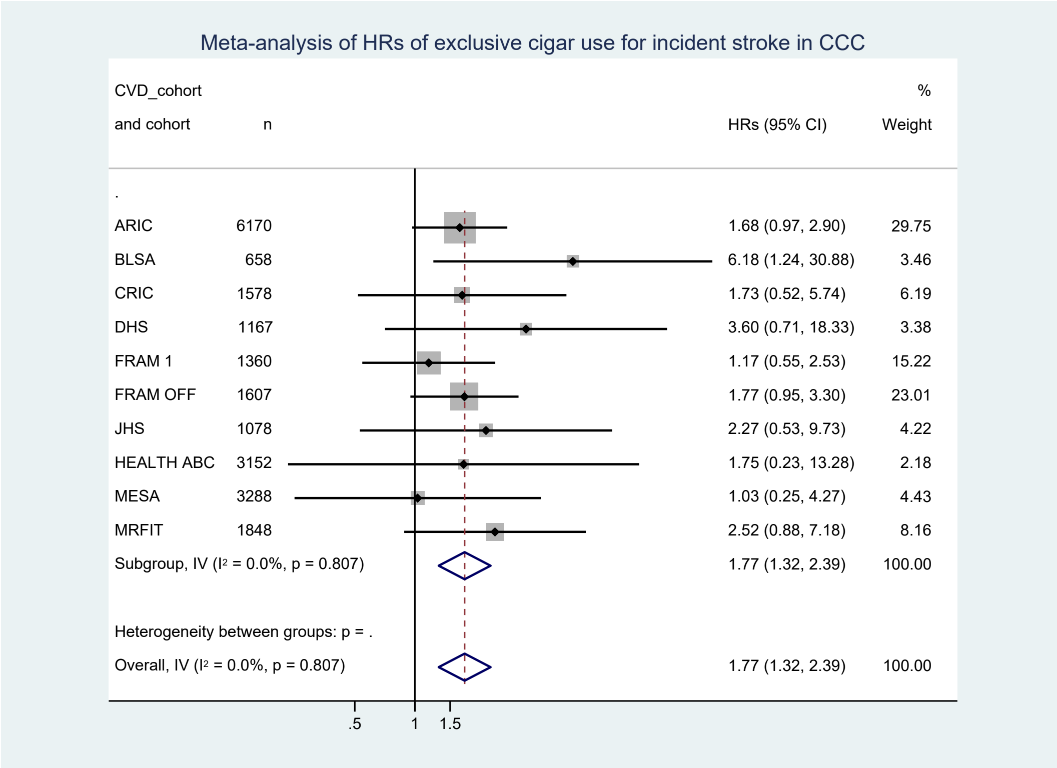

© 2025 Tasdighi E et al. *JAMA Network Open*.

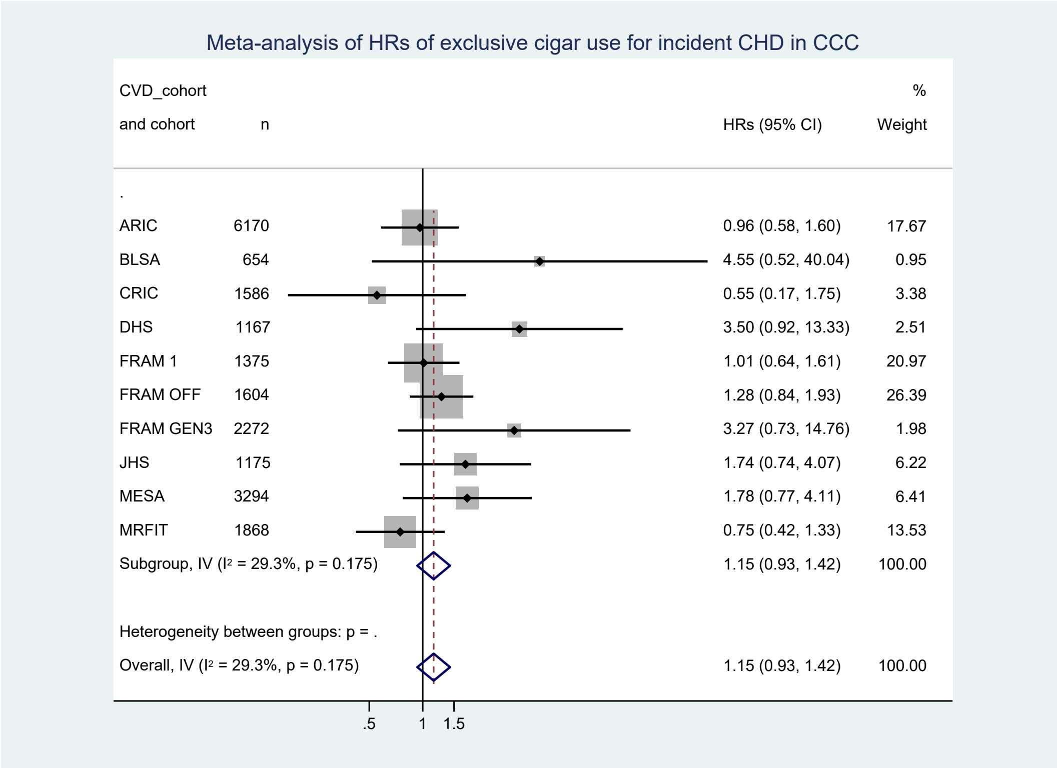

© 2025 Tasdighi E et al. *JAMA Network Open*.

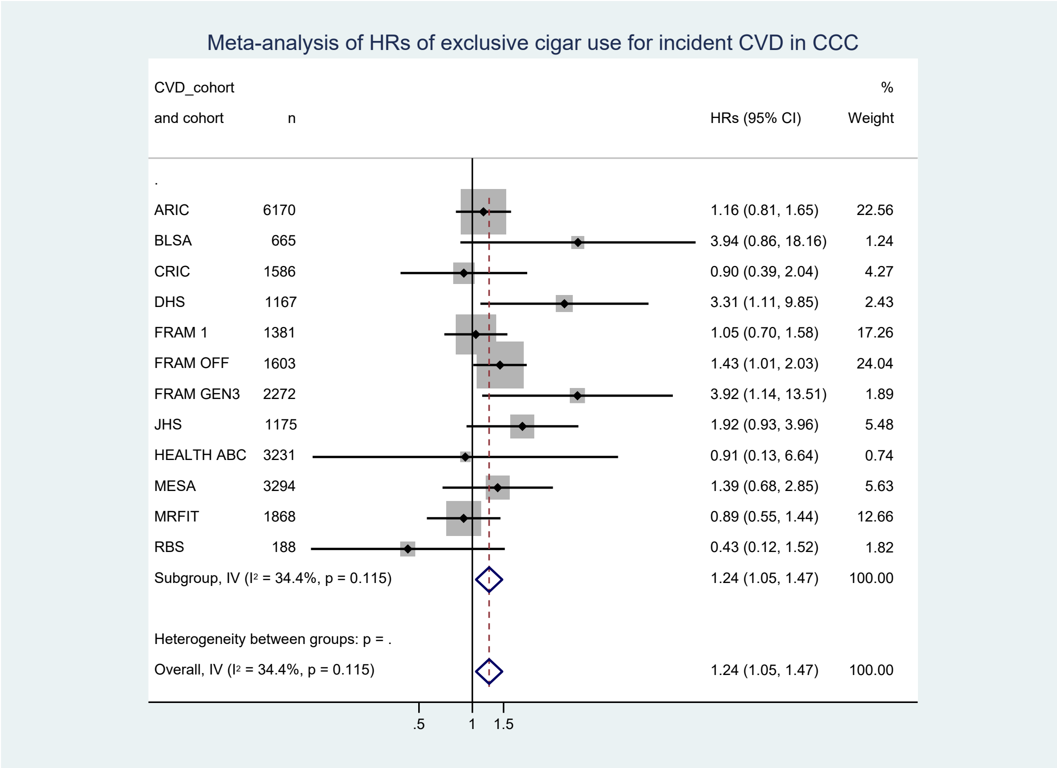

© 2025 Tasdighi E et al. *JAMA Network Open*.

Meta-analysis of HRs of exclusive cigar use for incident HF in CCC

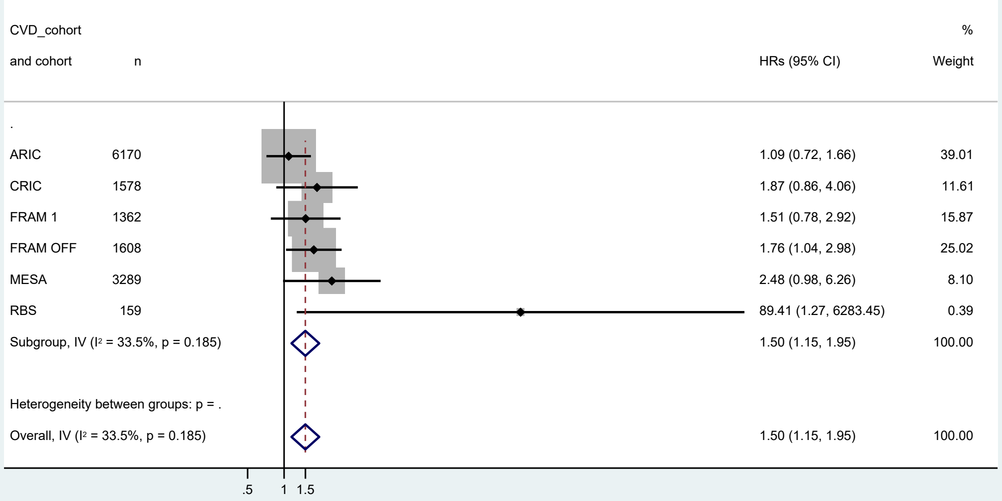

© 2025 Tasdighi E et al. *JAMA Network Open*.

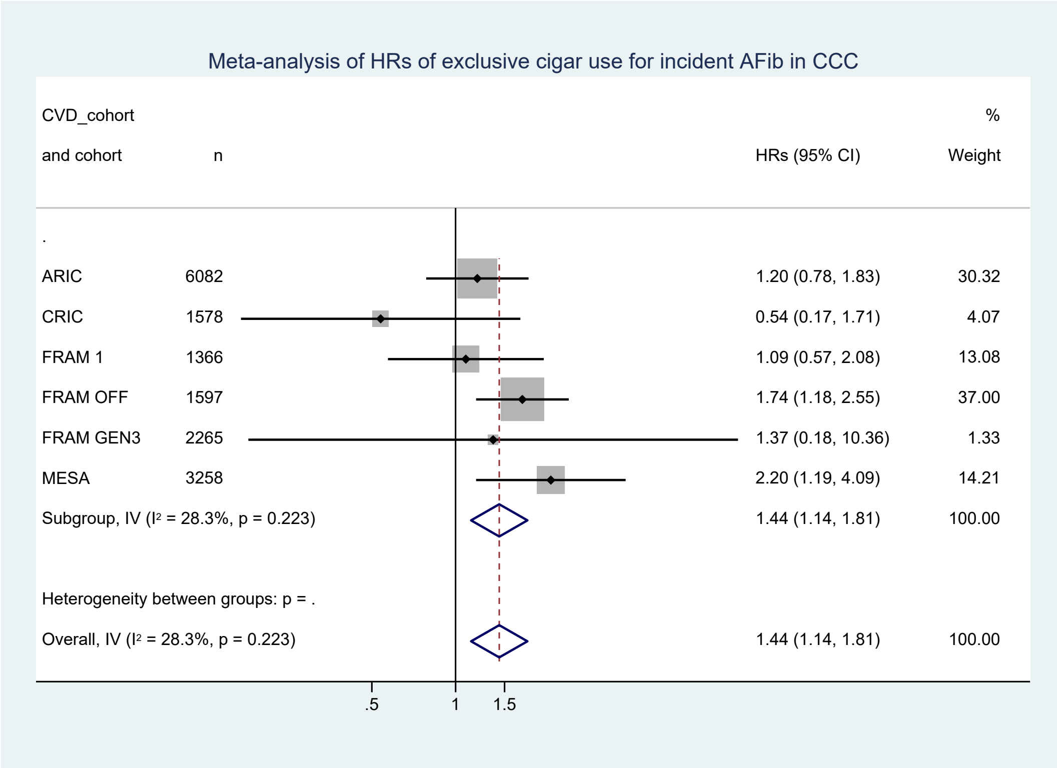

© 2025 Tasdighi E et al. *JAMA Network Open*.

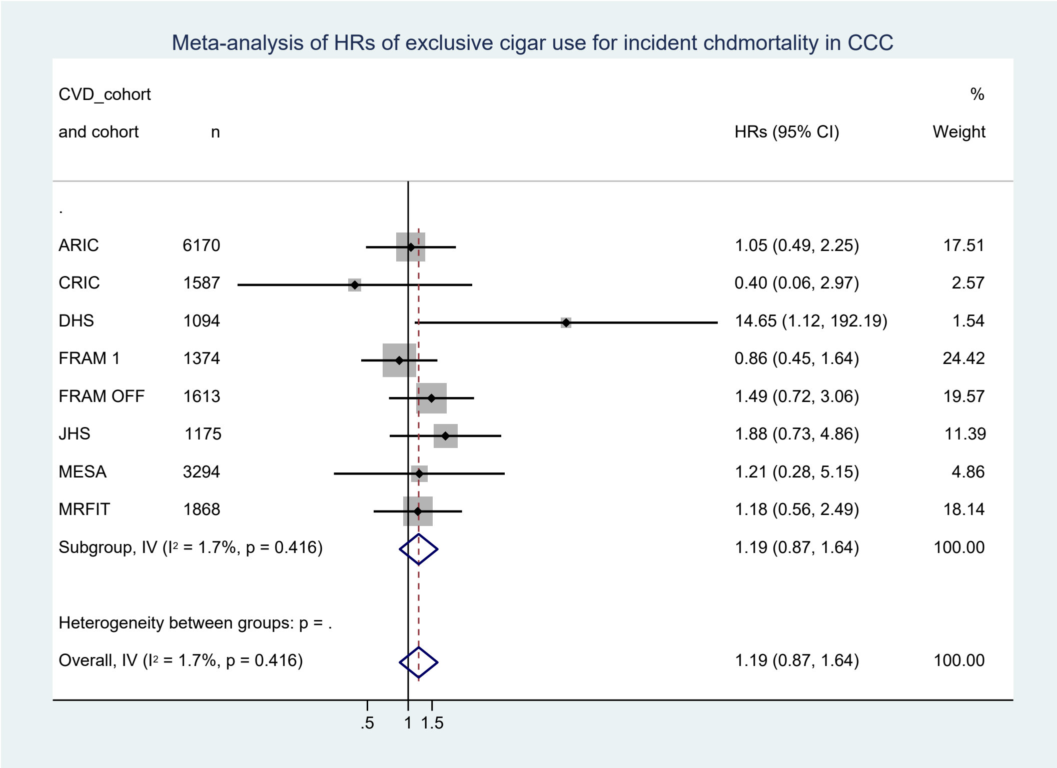

© 2025 Tasdighi E et al. *JAMA Network Open*.

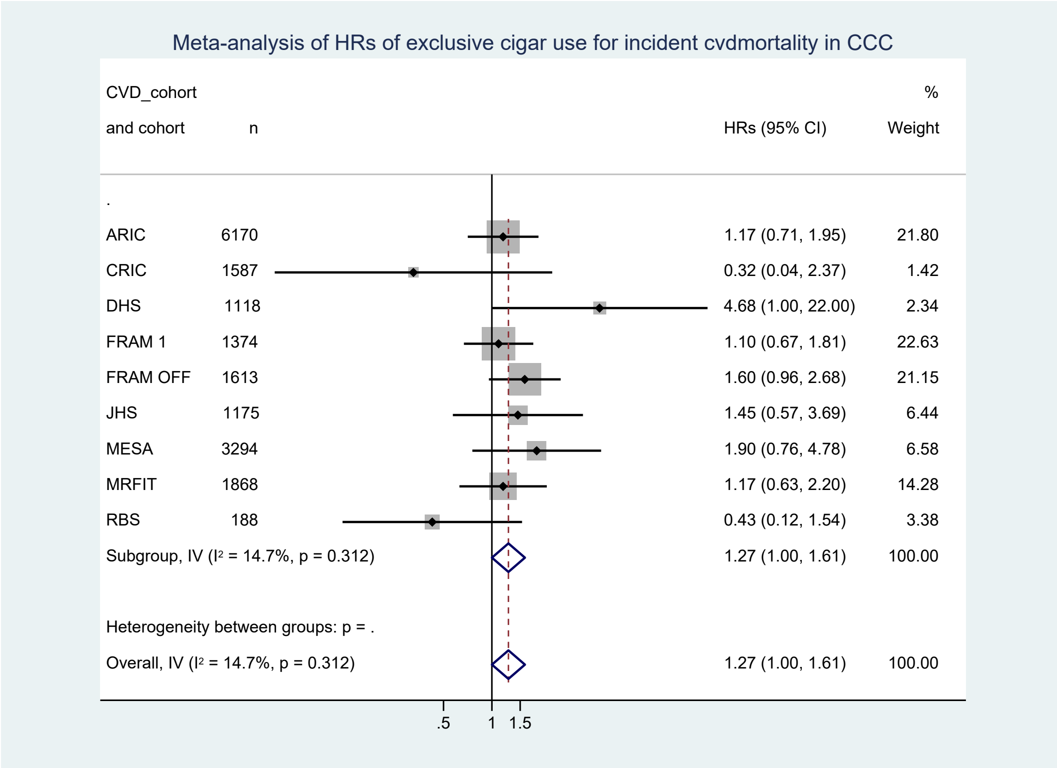

© 2025 Tasdighi E et al. *JAMA Network Open*.

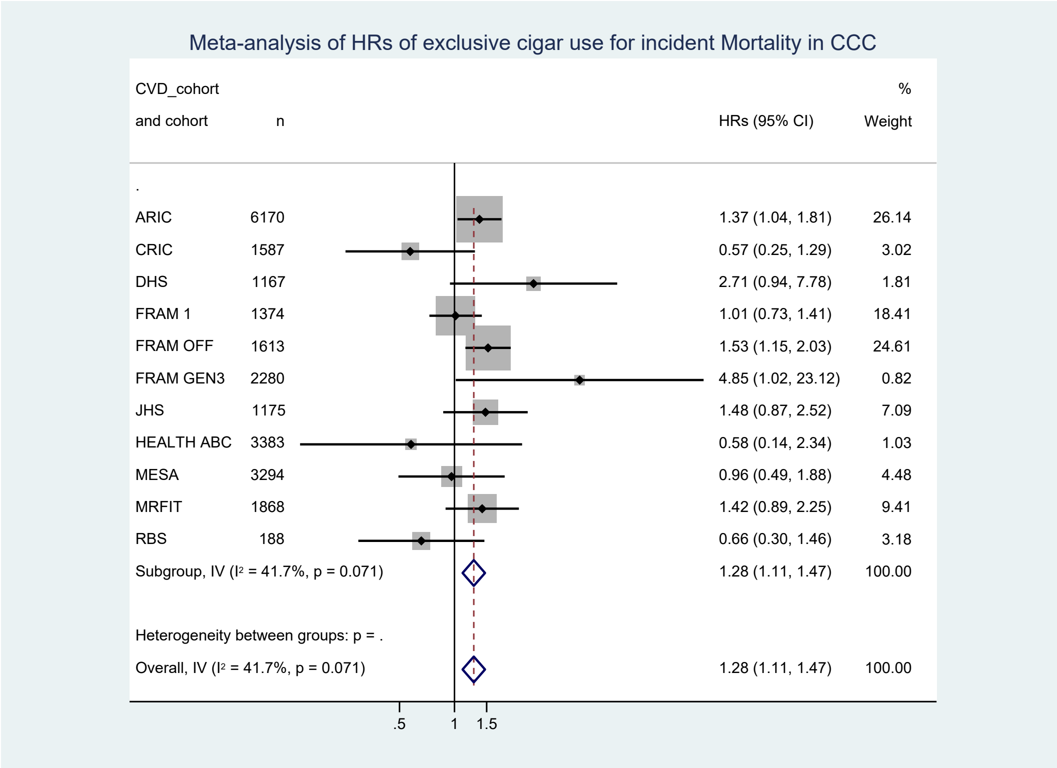

© 2025 Tasdighi E et al. *JAMA Network Open*.

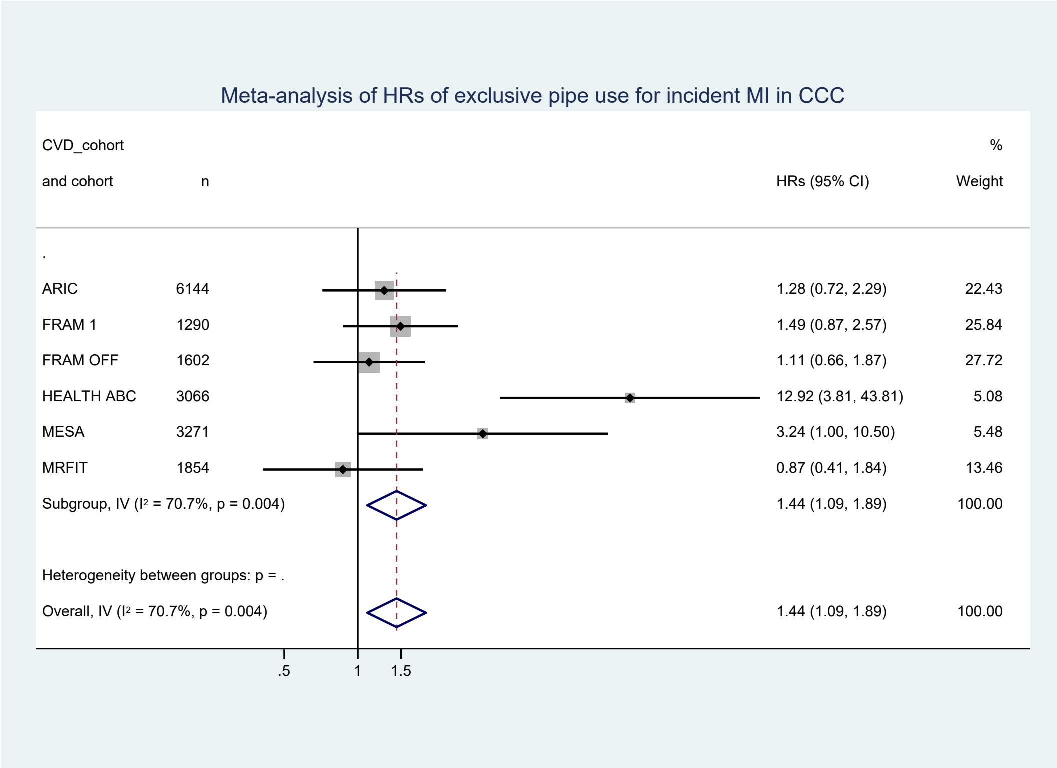

© 2025 Tasdighi E et al. *JAMA Network Open*.

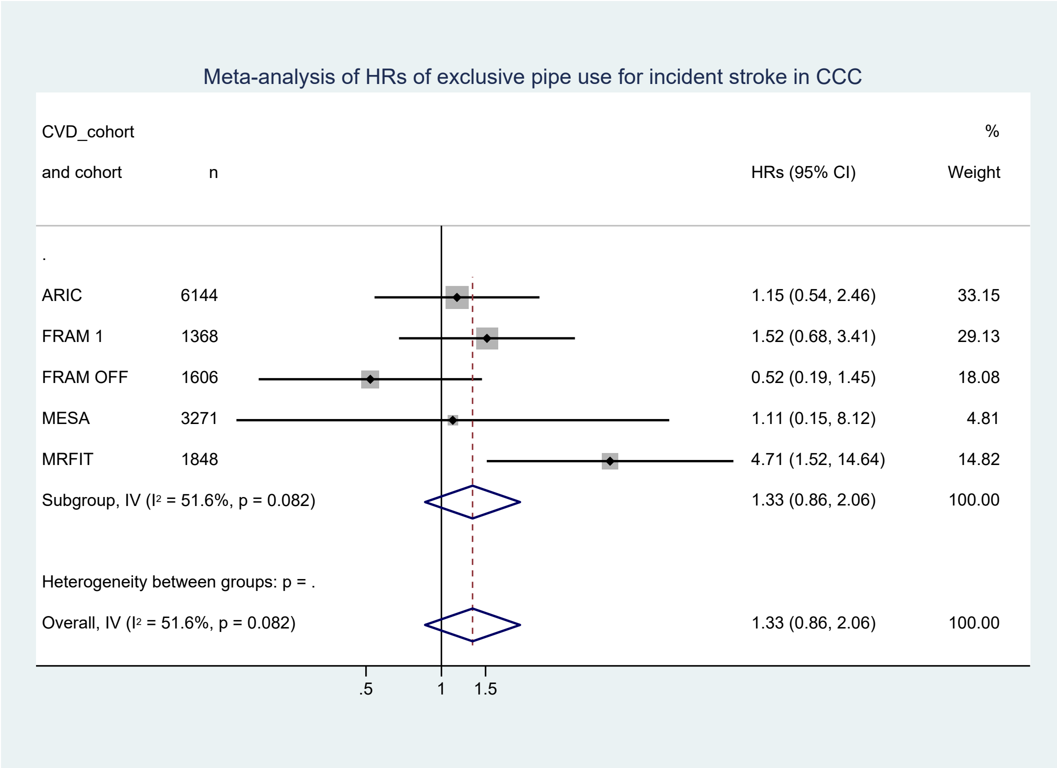

© 2025 Tasdighi E et al. *JAMA Network Open*.

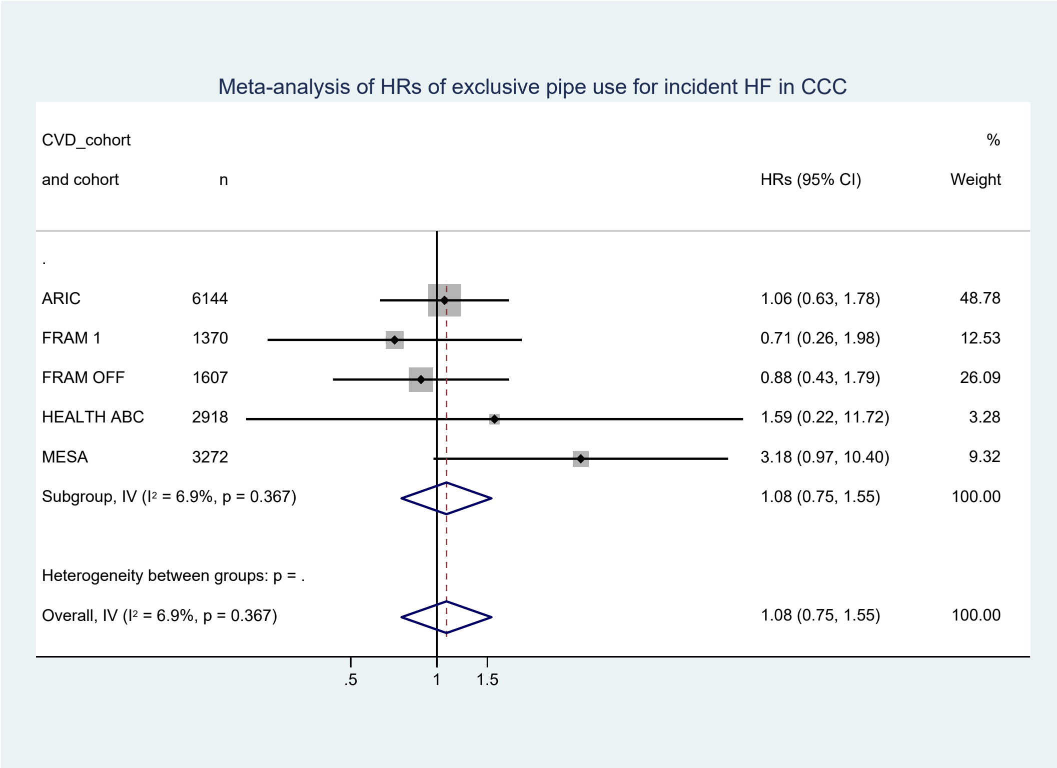

© 2025 Tasdighi E et al. *JAMA Network Open*.

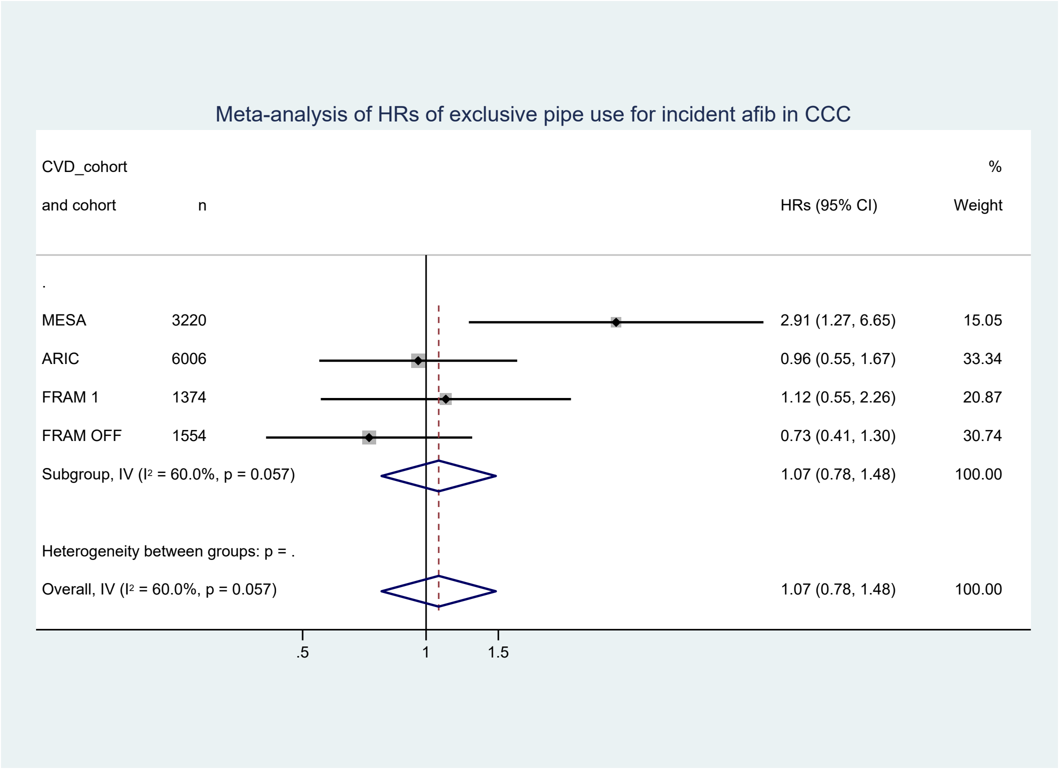

© 2025 Tasdighi E et al. *JAMA Network Open*.

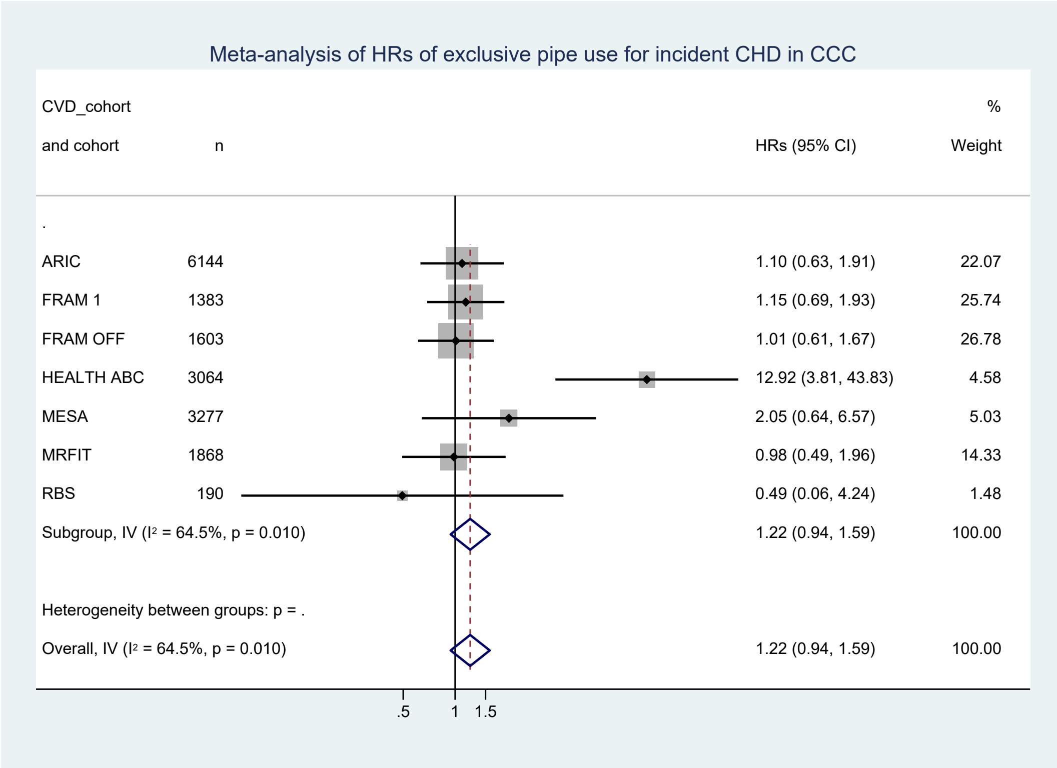

© 2025 Tasdighi E et al. *JAMA Network Open*.

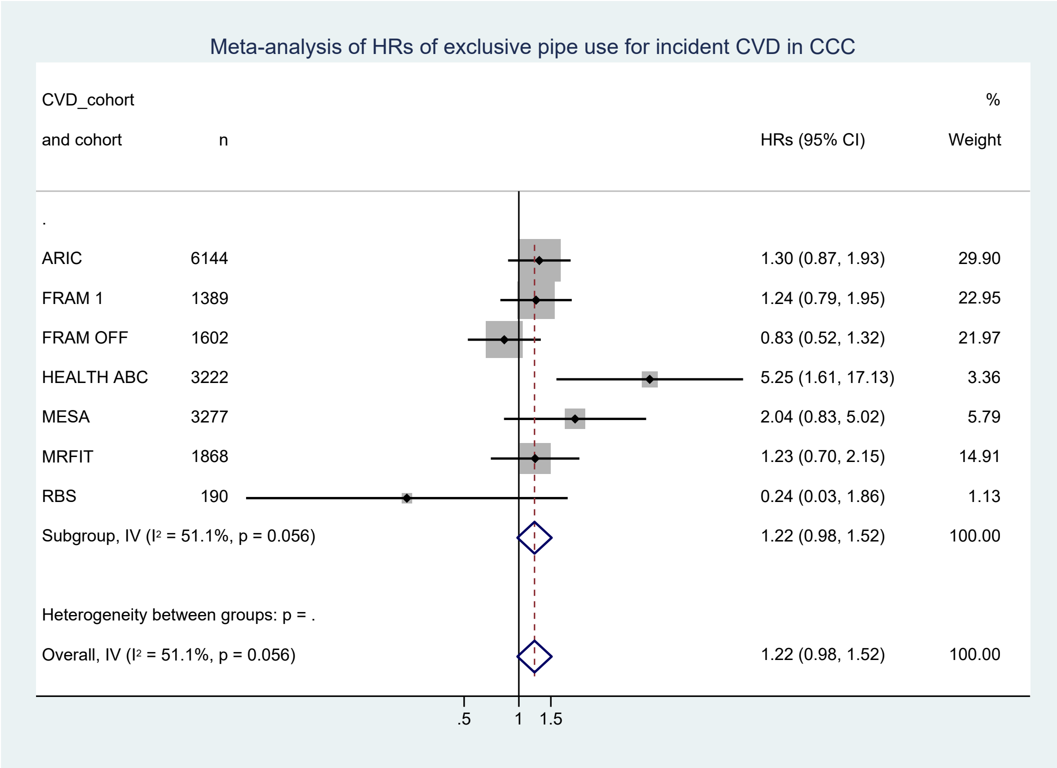

© 2025 Tasdighi E et al. *JAMA Network Open*.

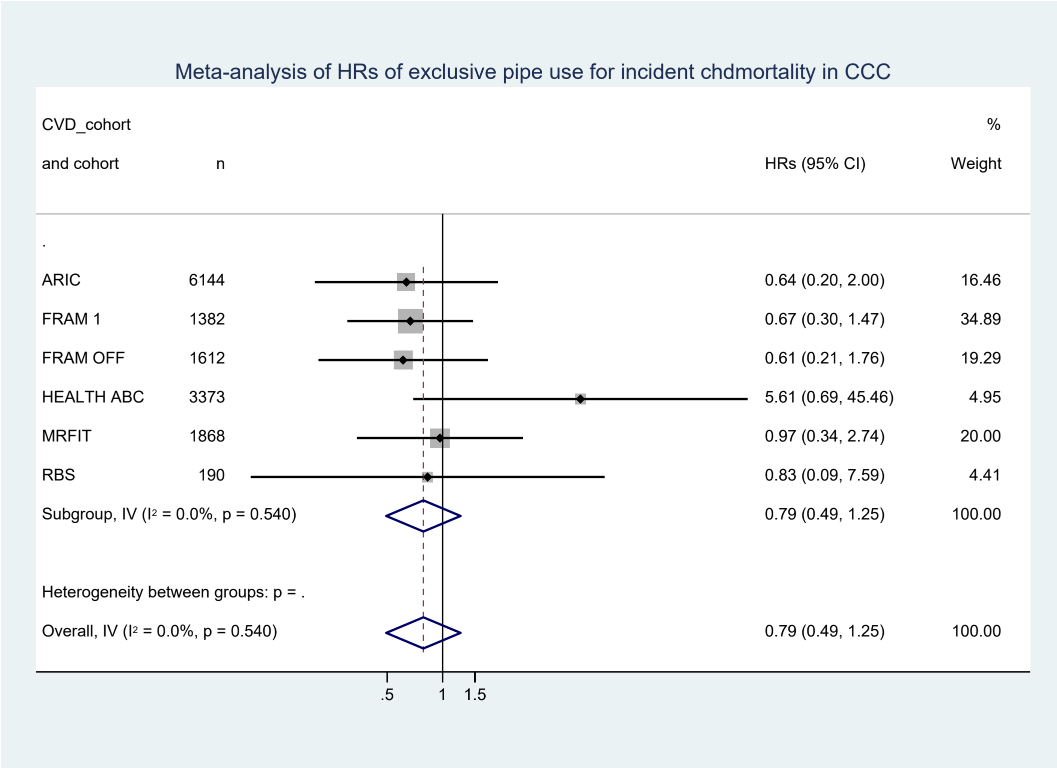

© 2025 Tasdighi E et al. *JAMA Network Open*.

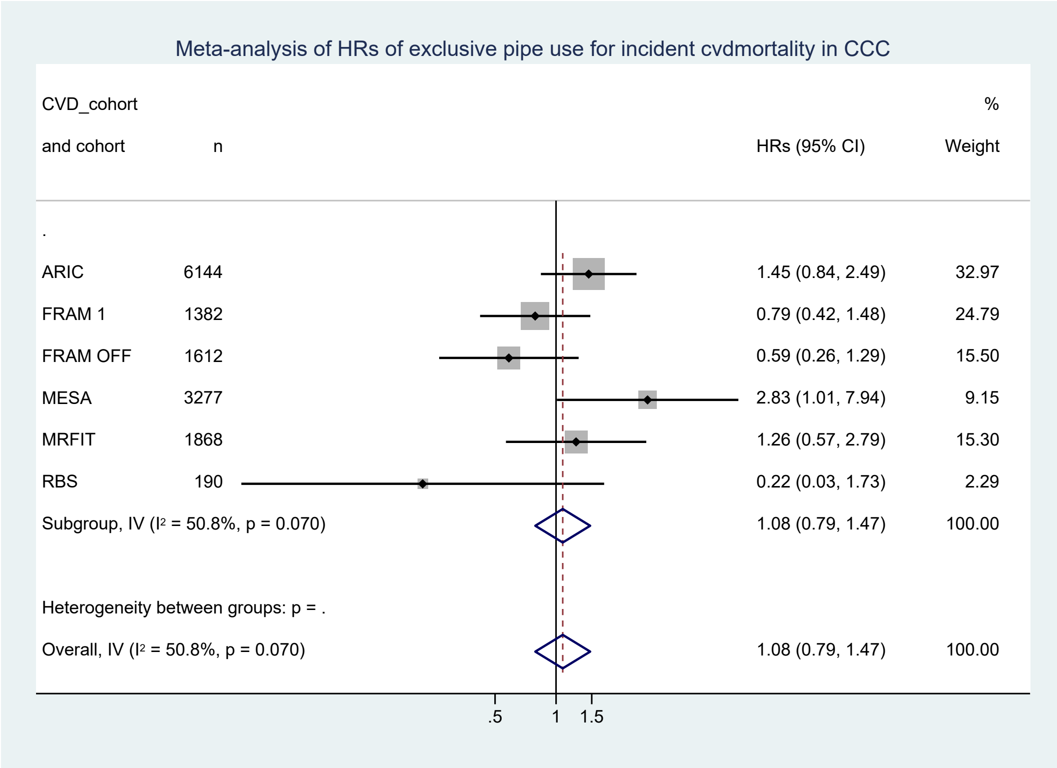

© 2025 Tasdighi E et al. *JAMA Network Open*.

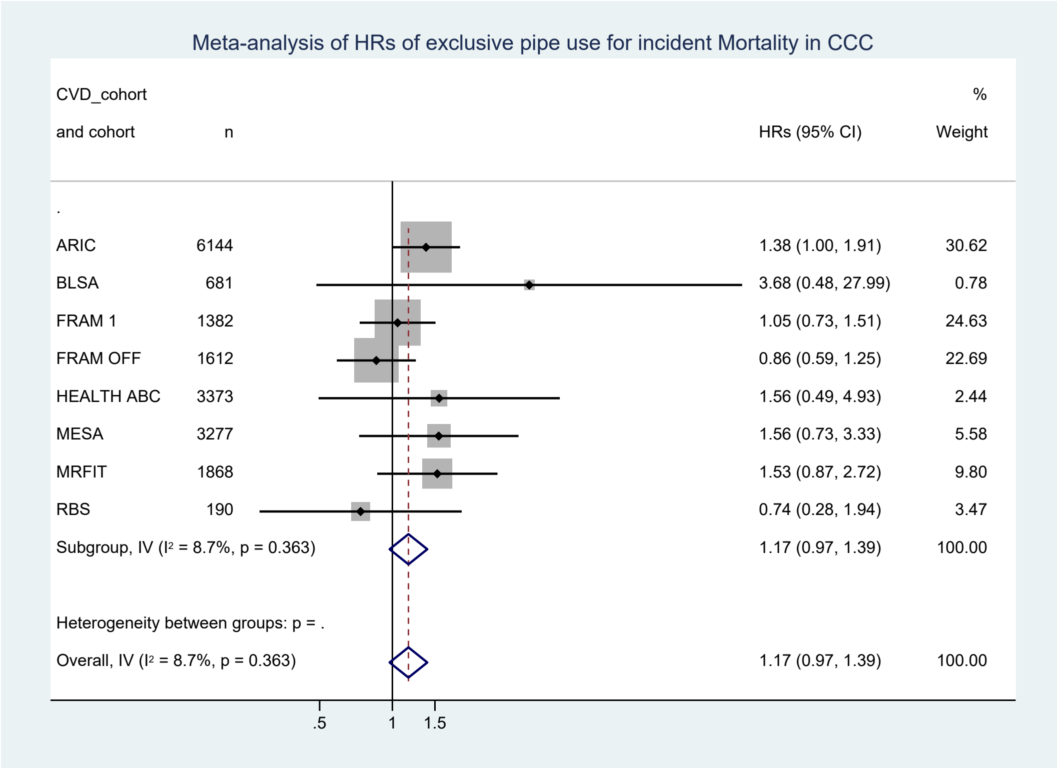

© 2025 Tasdighi E et al. *JAMA Network Open*.

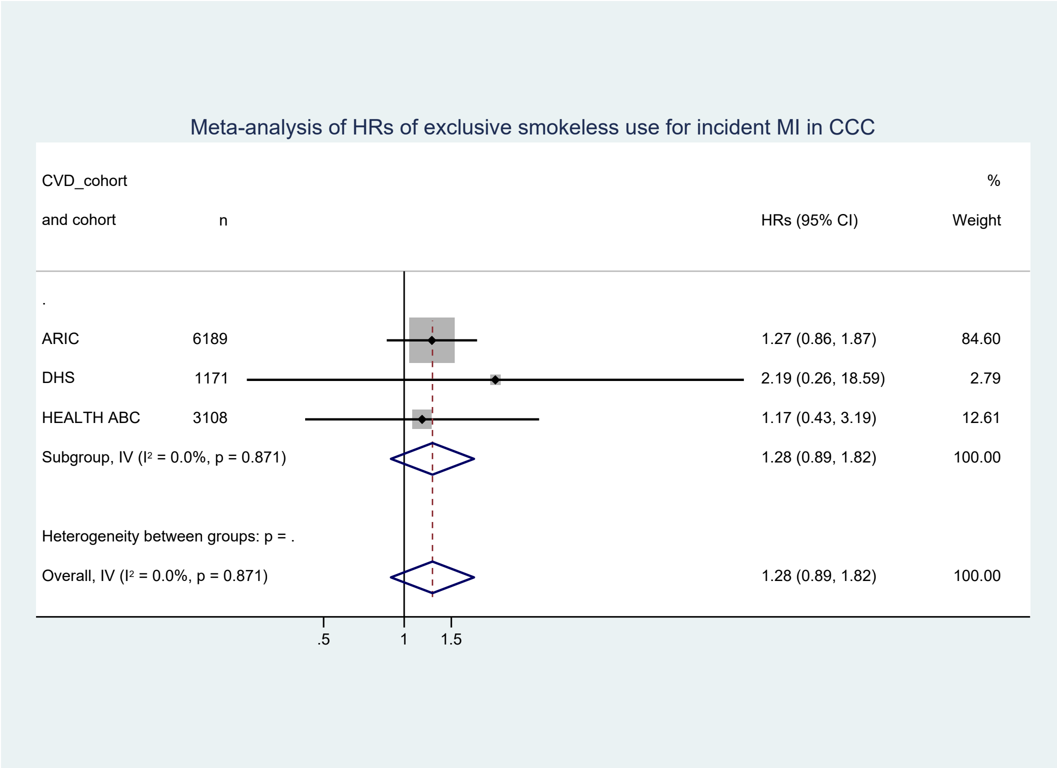

© 2025 Tasdighi E et al. *JAMA Network Open*.

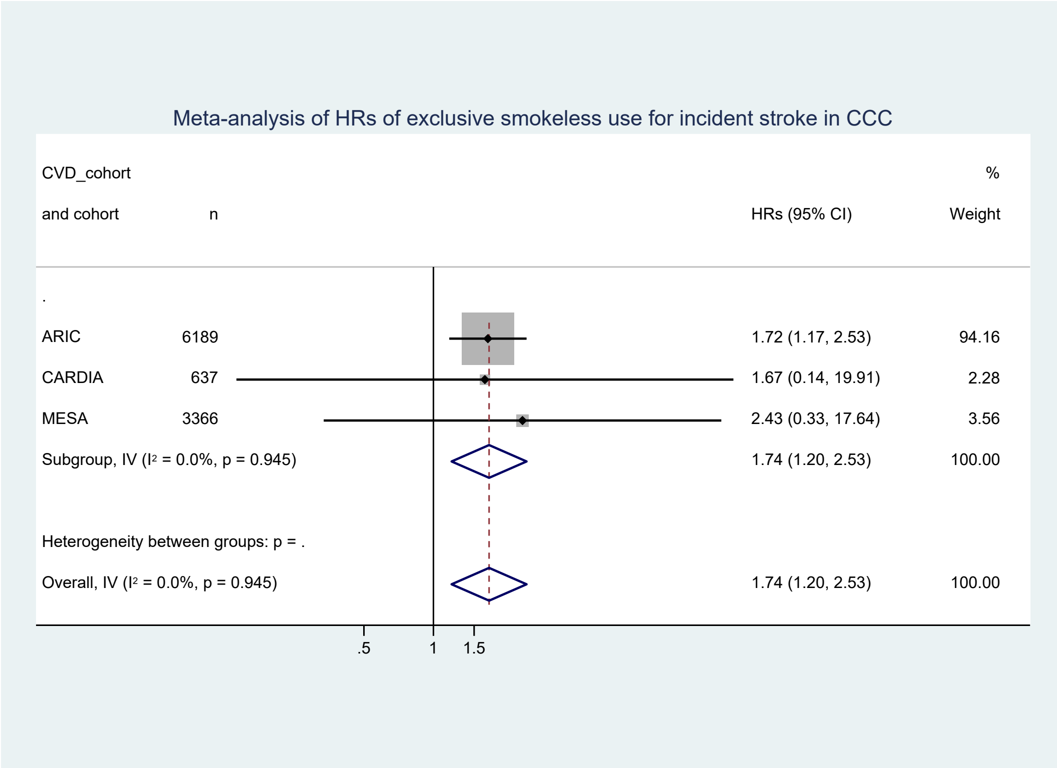

© 2025 Tasdighi E et al. *JAMA Network Open*.

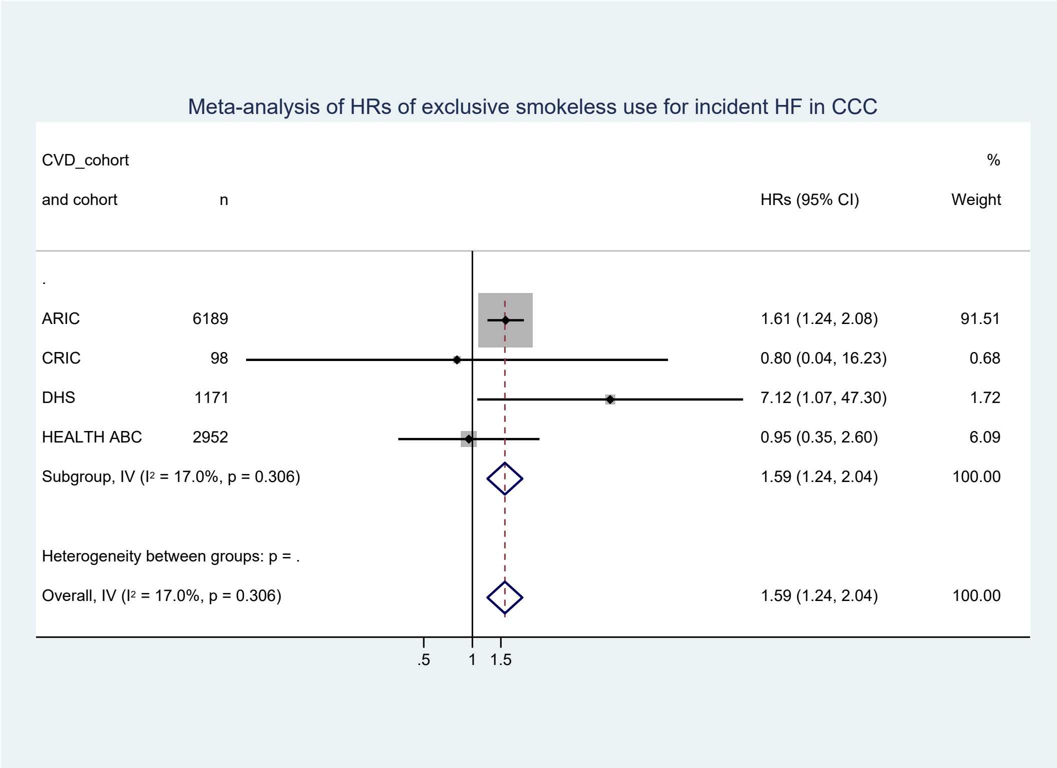

© 2025 Tasdighi E et al. *JAMA Network Open*.

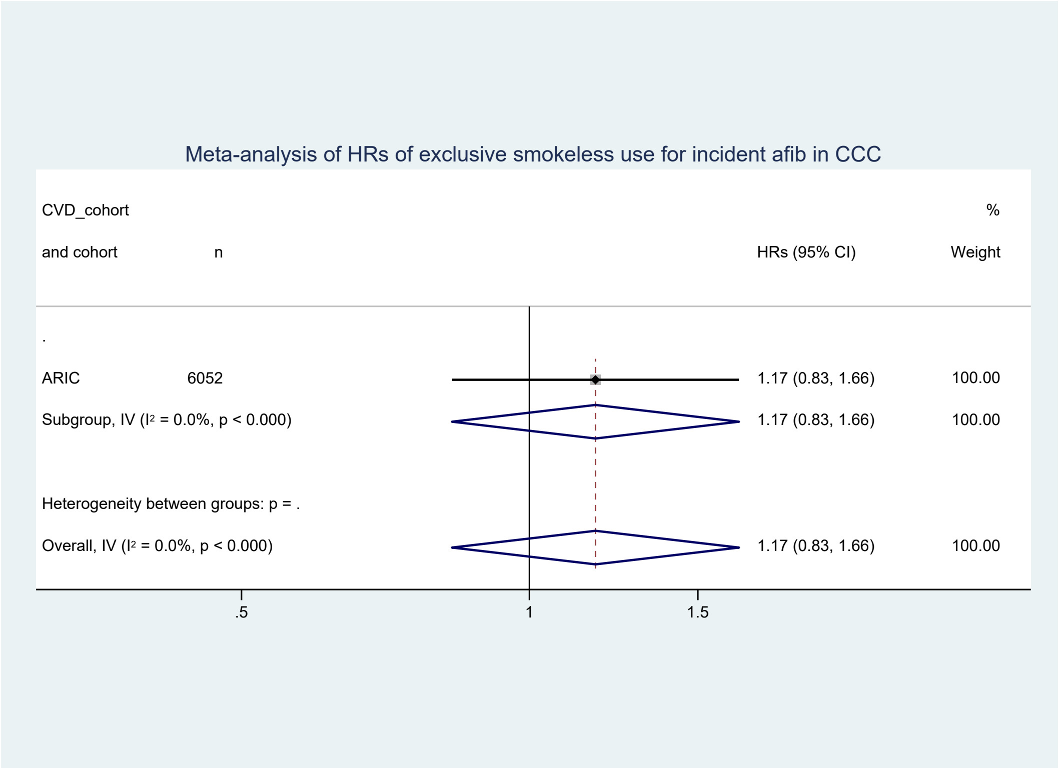

© 2025 Tasdighi E et al. *JAMA Network Open*.

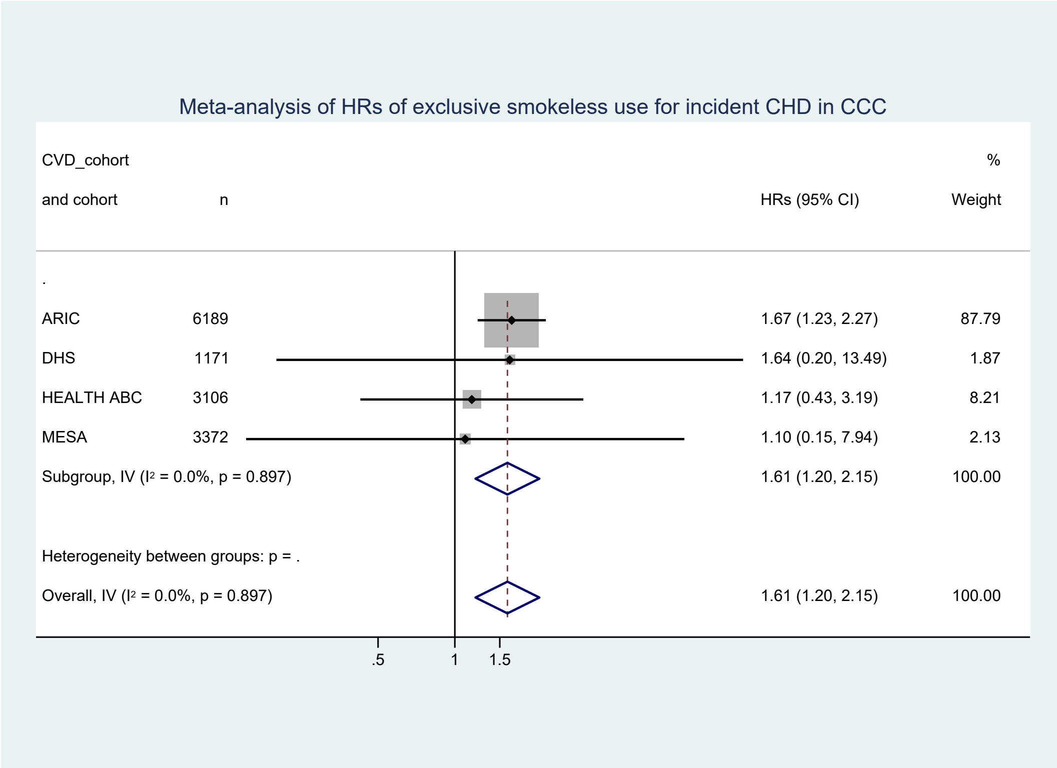

© 2025 Tasdighi E et al. *JAMA Network Open*.

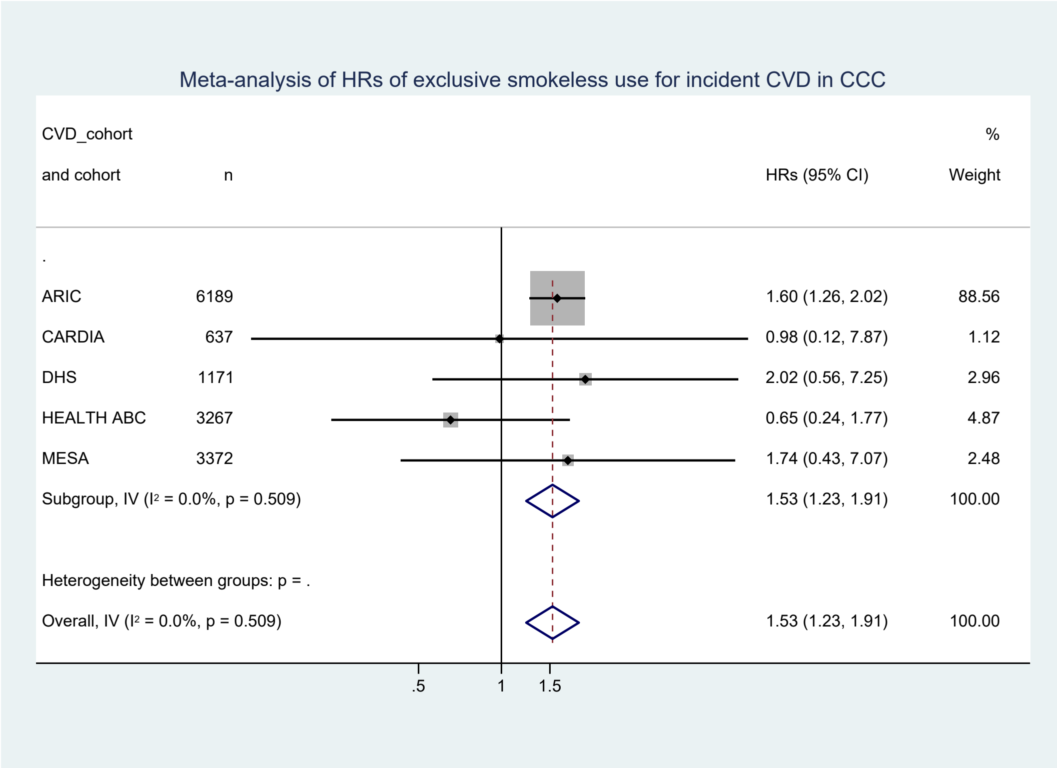

© 2025 Tasdighi E et al. *JAMA Network Open*.

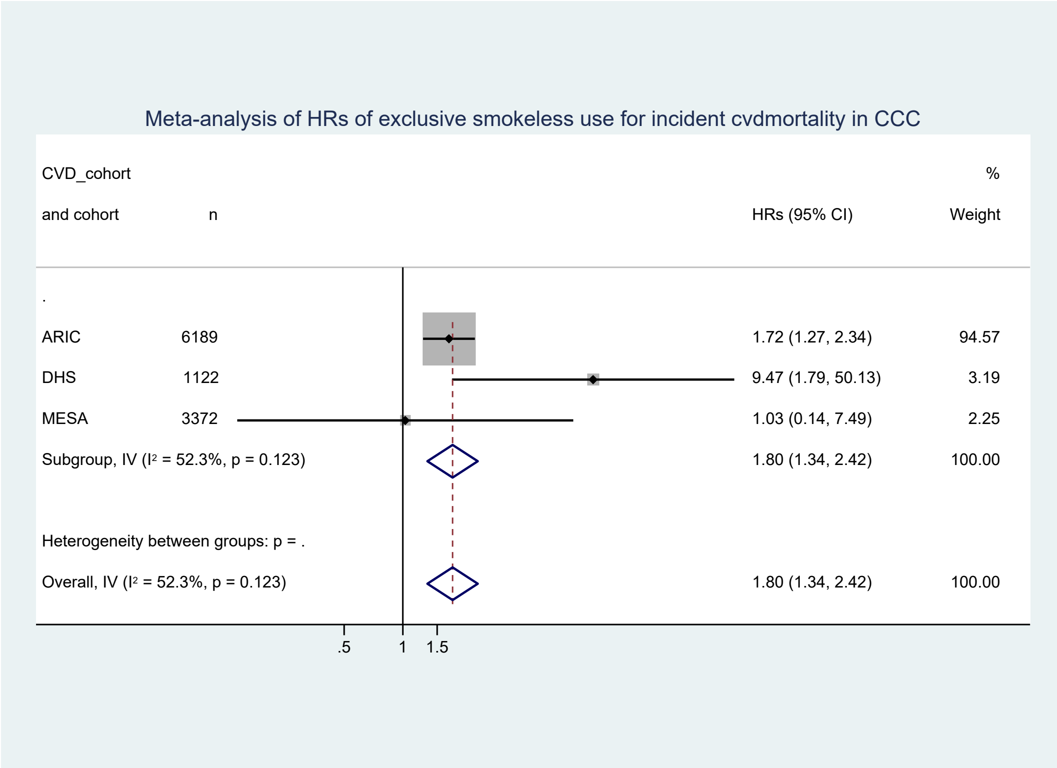

© 2025 Tasdighi E et al. *JAMA Network Open*.

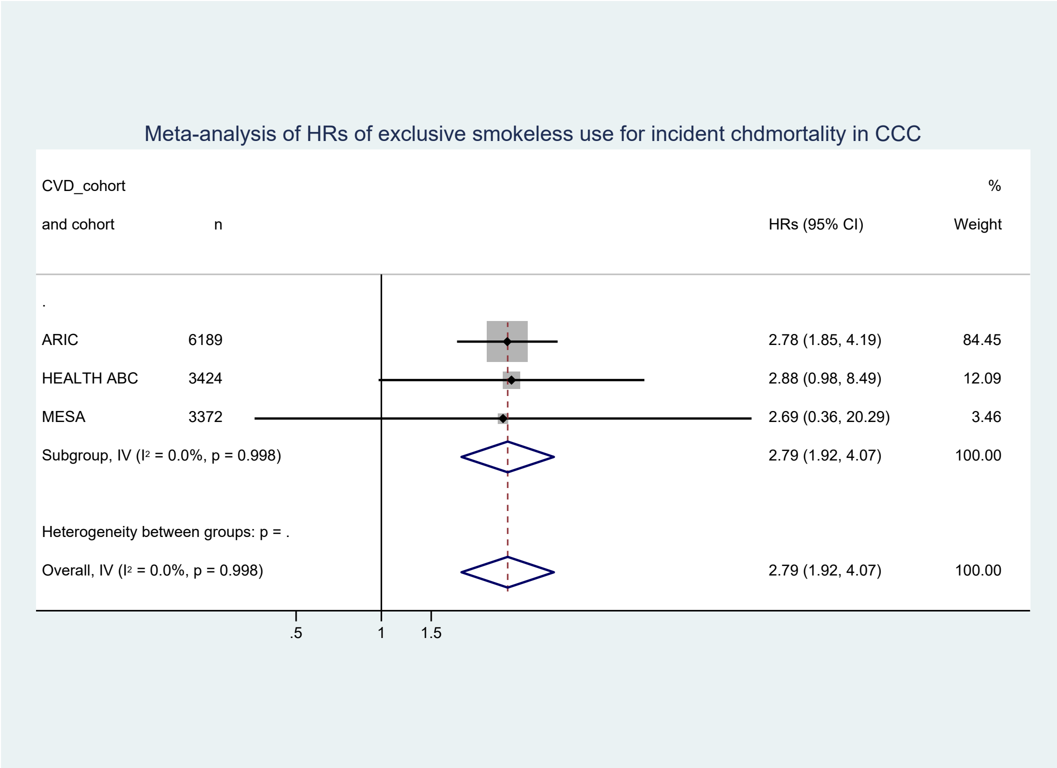

© 2025 Tasdighi E et al. *JAMA Network Open*.

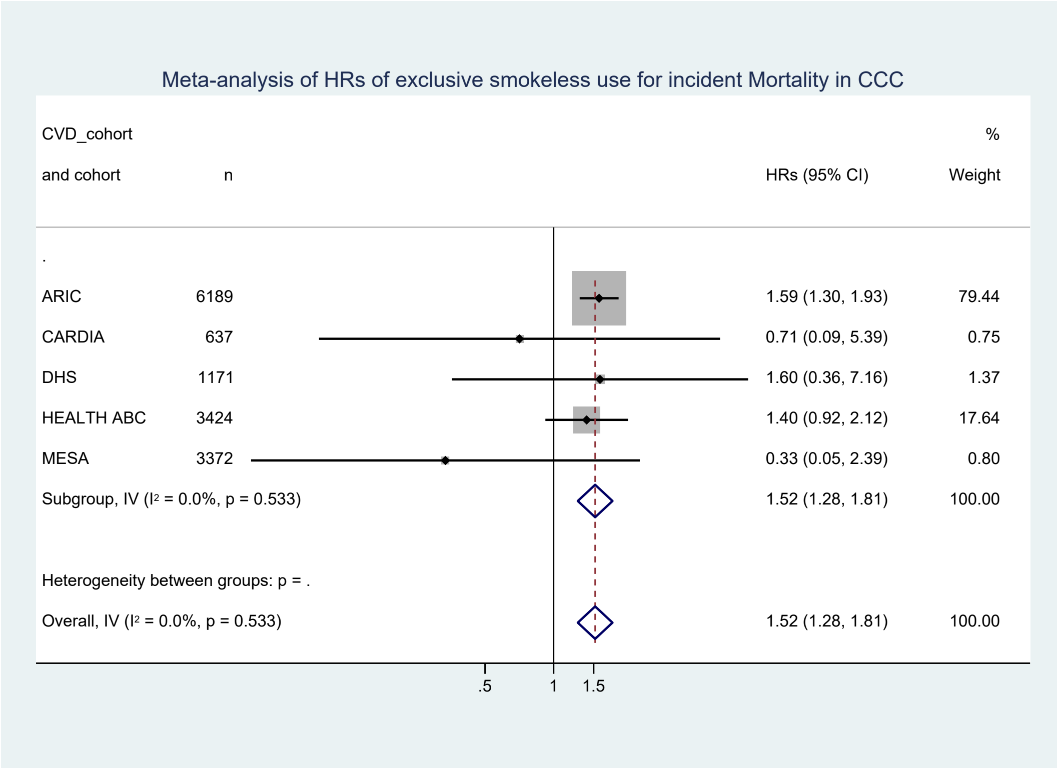

© 2025 Tasdighi E et al. *JAMA Network Open*.

Table e11 Supplementary. Association between current use of traditional and non-cigarette tobacco products and health outcomes compared with non-use of the given tobacco product based on non-imputed data in survival analysis models

	Cigarette	Cigar	Pipe	Smokeless
**MI**				
Model 2	**1.79 (1.69-1.90)**	1.11 (0.94-1.32)	1.20 (0.98-1.48)	**1.20 (1.03-1.39)**
**Stroke**				
Model 2	**1.65 (1.53-1.77)**	**1.25 (1.01-1.55)**	1.09 (0.82-1.46)	1.09 (0.90-1.32)
**CHD**				
Model 2	**1.81 (1.72-1.91)**	1.11 (0.96-1.29)	1.09 (0.89-1.32)	**1.19 (1.04-1.36)**
**CVD**				
Model 2	**1.83 (1.76-1.91)**	**1.15 (1.02-1.30)**	1.10 (0.94-1.29)	**1.19 (1.08-1.32)**
**Heart failure**				
Model 2	**2.05 (1.94-2.17)**	**1.29 (1.10-1.51)**	**1.23 (1.01-1.49)**	**1.20 (1.06-1.36)**
**Atrial fibrillation**				
Model 2	**1.60 (1.51-1.71)**	**1.32 (1.13-1.53)**	1.00 (0.82-1.21)	1.13 (0.96-1.33)
**CHD Mortality**				
Model 2	**2.00 (1.85-2.17)**	1.20 (0.96-1.50)	0.99 (0.71-1.38)	**1.31 (1.08-1.59)**
**CVD Mortality**				
Model 2	**1.93 (1.82-2.05)**	1.18 (0.99-1.40)	1.00 (0.79-1.28)	**1.23 (1.07-1.41)**
**All-cause mortality**				
Model 2	**2.19 (2.13-2.26)**	**1.13 (1.03-1.24)**	1.08 (0.96-1.22)	**1.21 (1.11-1.30)**

*Reference group consists of individuals who have never smoked the specific tobacco product under consideration.
Model 1 adjusted for age, sex, race and ethnicity, former/cigarette smoking status, education status, and history of coronary heart disease cohort.
Model 2 adjusted for age, sex, race and ethnicity, former/cigarette smoking status, education status, history of coronary heart disease, cohort, body mass index, hypertension, diabetes, antihypertensive and lipid-lowering
*Both model 1 and model 2 were not adjusted for former/cigarette smoking status for cigarette tobacco analysis
MI: myocardial infarction; CHD: coronary heart disease; CVD: cardiovascular disease

© 2025 Tasdighi E et al. *JAMA Network Open*.

Table e12 supplementary. Association between Sole and Exclusive use of non-cigarette tobacco products and health outcomes compared with non-use of the given tobacco product including the frailty term in survival analysis models

	Cigar use status		Pipe use status		Smokeless use status	
	Sole[1]	Exclusive[2]	Sole	Exclusive	Sole	Exclusive
**MI**						
Model 2	1.11 (0.95-1.30)	1.11 (0.90-1.37)	**1.37 (1.15-1.65)**	**1.28 (1.00-1.68)**	**1.41 (1.16-1.72)**	1.28 (0.97-1.69)
**Stroke**						
Model 2	**1.34 (1.10-1.62)**	**1.53 (1.19-1.97)**	1.15 (0.92-1.45)	1.08 (0.70-1.65)	1.19 (0.88-1.62)	1.19 (0.86-1.64)
**CHD**						
Model 2	**1.16 (1.02-1.33)**	1.14 (0.95-1.37)	**1.36 (1.16-1.59)**	1.10 (0.85-1.43)	**1.25 (1.03-1.50)**	**1.38 (1.09-1.74)**
**CVD**						
Model 2	**1.19 (1.07-1.32)**	**1.26 (1.09-1.46)**	**1.32 (1.16-1.49)**	1.11 (0.89-1.38)	**1.22 (1.04-1.42)**	**1.37 (1.15-1.63)**
**Heart failure**						
Model 2	**1.33 (1.13-1.57)**	**1.36 (1.09-1.69)**	**1.42 (1.22-1.66)**	0.97 (0.67-1.38)	**1.39 (1.11-1.75)**	**1.69 (1.39-2.07)**
**Atrial fibrillation**						
Model 2	**1.23 (1.04-1.45)**	**1.32 (1.07-1.64)**	**1.30 (1.07-1.57)**	1.02 (0.75-1.40)	1.23 (0.99-1.52)	1.09 (0.79-1.48)
**CHD Mortality**						
Model 2	**1.30 (1.06-1.59)**	1.20 (0.91-1.59)	**1.47 (1.16-1.87)**	0.75 (0.48-1.19)	1.13 (0.85-1.50)	**1.69 (1.22-2.34)**
**CVD Mortality**						
Model 2	**1.29 (1.11-1.49)**	**1.39 (1.14-1.69)**	**1.37 (1.16-1.62)**	1.01 (0.74-1.38)	1.11 (0.88-1.38)	**1.54 (1.23-1.93)**
**All-cause mortality**						
Model 2	**1.31 (1.20-1.43)**	**1.24 (1.10-1.40)**	**1.46 (1.33-1.60)**	1.12 (0.93-1.34)	**1.29 (1.13-1.47)**	**1.40 (1.22-1.60)**

The reference group includes only participants with never cigarette use and no reported non-cigarette products for both sole and exclusive analysis.
1 Sole use is defined as current non-cigarette tobacco use without current cigarette use.
2 Exclusive use is defined as current non-cigarette tobacco without any history of cigarette use.
Model 1 adjusted for age, sex, race and ethnicity, former/cigarette smoking status, education status, and history of coronary heart disease cohort.
Model 2 adjusted for age, sex, race and ethnicity, former/cigarette smoking status, education status, history of coronary heart disease, cohort, body mass index, hypertension, diabetes, antihypertensive and lipid-lowering
MI: myocardial infarction; CHD: coronary heart disease; CVD: cardiovascular disease

© 2025 Tasdighi E et al. *JAMA Network Open*.
