# Supplementary material for: Cigar, Pipe, and Smokeless Tobacco Use and Cardiovascular Outcomes From Cross Cohort Collaboration
Source: JAMA Netw Open. 2025 Jan 13;8(1):e2453987. doi: 10.1001/jamanetworkopen.2024.53987 (PMC11731180; doi:10.1001/jamanetworkopen.2024.53987)
Supplement: Supplement 2. — Data Sharing Statement [file jamanetwopen-e2453987-s002.pdf]

Data Sharing Statement

Tasdighi. Cigar, Pipe, and Smokeless Tobacco Use and Cardiovascular Outcomes From Cross Cohort Collaboration. *JAMA Netw Open*. Published online January 13, 2025. doi:10.1001/jamanetworkopen.2024.53987

Data
Data available: No

Additional Information
Explanation for why data not available: Since we collected and harmonized data from each cohort we are not at the liberty to share their data.
